# Candidate Genomic Features Associated with Persistence in *Enterococcus* spp.

**DOI:** 10.3390/microorganisms14040921

**Published:** 2026-04-19

**Authors:** Catarina Geraldes, Carolina Silva, Filipa Vale, Eva Cunha, Catarina Araújo, Mónica Nunes, Ricardo Dias, Luís Tavares, Joana Fernandes Guerreiro, Manuela Oliveira

**Affiliations:** 1 CIISA—Centre for Interdisciplinary Research in Animal Health, Faculty of Veterinary Medicine, University of Lisbon, 1300-477 Lisbon, Portugalevacunha@fmv.ulisboa.pt (E.C.); claraujo@fmv.ulisboa.pt (C.A.); ltavares@fmv.ulisboa.pt (L.T.); jguerreiro@fmv.ulisboa.pt (J.F.G.); 2 AL4AnimalS—Associate Laboratory for Animal and Veterinary Science, Faculty of Veterinary Medicine, University of Lisbon, 1300-477 Lisbon, Portugal; 3 BioISI—Biosystems & Integrative Sciences Institute, Faculty of Sciences, University of Lisbon, 1749-016 Lisbon, Portugal; afvale@ciencias.ulisboa.pt (F.V.); rpdias@ciencias.ulisboa.pt (R.D.); 4 cE3c—Centre for Ecology, Evolution and Environmental Changes & CHANGE—Global Change and Sustainability Institute, Faculty of Sciences, University of Lisbon, 1300-477 Lisbon, Portugal; msnunes@ciencias.ulisboa.pt

**Keywords:** *Enterococcus* spp., persistence, ciprofloxacin, healthcare associated infections, toxin-antitoxin systems, cold shock proteins, DNA repair

## Abstract

Bacterial persistence has been extensively studied as a possible explanation for strain survival under stress; however, in *Enterococcus* spp., this ability is still an understudied phenomenon. In this study, 40 *Enterococcus* spp. isolates of human clinical (n = 10), veterinary commensal (n = 10), veterinary clinical (n = 10) and veterinary environmental (hospital surfaces) (n = 10) origins, were exposed to a high concentration of ciprofloxacin. Time–kill curves were established, after which antimicrobial susceptibility profiles were reassessed. Subsequently, the only presumptive persister was selected for Whole-Genome Sequencing, together with one isolate showing no evidence of persister formation. Comparative genomic analyses were conducted to identify genetic variations between exposed and non-exposed isolates and to explore potential genetic determinants associated with persistence. Observed genetic features present in the persister isolate included toxin–antitoxin systems, a cold-shock protein and the tyrosine-type recombinase/integrase XerC, which may represent putative candidates for further investigation. Interestingly, the majority of toxin–antitoxin system-associated genes were found in plasmids. This study represents an important step towards a better understanding of persistence development in *Enterococcus* spp.; however, validation using other methodologies such as RNA-sequencing is an important next step.

## 1. Introduction

*Enterococcus* spp. are opportunistic pathogens known for their resilience and ability to adapt to the adverse conditions present in the hospital environments [1,2]. In these last few years, they have been identified as being responsible for a significant percentage of healthcare-associated infections (HAIs) [3]. Their resilience and infection capacity are reinforced by their intrinsic and extrinsic resistance to many antimicrobials [2].

A crucial aspect of antimicrobial resistance is that, beyond its conventional definition, it encompasses an unusual mechanism involving the development of a heterogenous cell population capable of surviving antibiotic therapy, known as persistence [4,5]. Persisters are defined as a sub-population of cells that arises due to a triggered transition to a non-replicative, low-metabolic state, thereby reducing the availability of antimicrobial targets [4,5]. This implies that persistence is usually associated with survival to multiple antibiotics, even if they present distinct mechanisms of action [6]. In some bacterial species, spontaneous persistence, i.e., the development of a persister state in the absence of an external trigger, has also been described [4].

Development of persistence has been associated with the activation of certain metabolic pathways such as the stringent response, the toxin–antitoxin (TA) pathway, the SOS response, and the oxidative stress response [4,7,8,9]. In one study, persister development in *Enterococcus* spp. has been potentially associated with the production of stress proteins, such as AhpC, Dps and MsrA, responsible for bacterial protection against oxidative damage and other stressors [8].

Once the external stressor is removed and following a recovery period, these cells can revert to their original metabolic state, usually resulting in a population with unaltered antibiotic susceptibility when compared to the original population [6,8]. Although a link between persistence and resistance development has been reported [10], persister formation is more frequently associated with the absence of genetic mutations [8]. Additionally, resistance development is also conventionally associated with cell replication in the presence of antibiotics, contrarily to what is seen in persistence [4].

This dissimilarity is especially seen in vitro, through time–kill assays, where persister development is generally characterized by a biphasic killing curve [6,7,8]. This pattern corresponds to a rapid decline of the original population of bacterial cells, followed by a plateau associated with persister survival, without subsequent bacterial multiplication [4,5]. Persisters have also been frequently associated with biofilm formation [6,7,11].

In this study, *Enterococcus* spp. isolates from four different origins were subjected to a protocol previously described for persister formation in this genus [8]. Isolates exhibiting contrasting phenotypic responses were subsequently analyzed through Whole-Genome Sequencing (WGS) to confirm the absence of genomic alterations following antibiotic exposure, and to explore potential genetic features that may distinguish isolates with and without the ability to form persister cells in the described conditions. This approach aims to identify putative candidate genes that could be prioritized for future functional validation.

## 2. Materials and Methods

### 2.1. Isolate Characterization

To study the development of persistence in *Enterococcus* spp., four different groups were used in this study, each consisting of 10 isolates, for a total of 40 bacteria. The first group was composed of 10 *Enterococcus faecalis*, collected from diabetic foot ulcers in human patients [12]; the second group included 10 *Enterococcus faecium*, collected from the surfaces of the veterinary Biological Isolation and Containment Unit of the Veterinary Teaching Hospital of the Faculty of Veterinary Medicine of the University of Lisbon (FMV-ULisbon) [13,14]; the third group comprised 5 *E. faecium*, 4 *E. faecalis* and 1 *Enterococcus* sp., obtained at the Laboratory of Microbiology and Immunology of FMV-ULisbon from urine samples of dogs with urinary tract infections [15]; and the fourth group was composed by 5 *E. faecalis*, 3 *E. faecium* and 2 *E. hirae*, collected from the oral cavity of dogs [16]. The inclusion of bacteria from different origins allowed for the testing of a more variable genetic background. It also allowed for the inclusion of different possible sources of relevant bacteria within the hospital environment.

All isolates had been previously identified at species level through PCR [12,14,15,16].

### 2.2. Development and Characterization of a Persister Isolates

#### 2.2.1. Determination of the Minimal Inhibitory Concentration (MIC) of Ciprofloxacin

The Minimal Inhibitory Concentrations (MICs) of ciprofloxacin were determined for all isolates using the broth microdilution method, based on the Clinical and Laboratory Standards Institute (CLSI) guidelines and cut-offs [17,18,19]. *E. faecalis* ATCC 29212 was included as a quality control strain in all assays [17,18].

First, ciprofloxacin (Sigma-Aldrich, St. Louis, MO, USA) was suspended in 0.1 N of hydrochloric acid (HCl) (VWR^®^ International, Leuven, Belgium) to obtain a stock solution of 2 mg/mL. This suspension was stored at −20 °C until further use. In the beginning of each assay, a working solution of this antibiotic was serially diluted, using two-fold dilutions, in Mueller-Hinton Cation Adjusted (MHCA) broth (Becton Dickinson, Franklin Lakes, NJ, USA) in round-bottom 96-well plates (VWR^®^ International, Leuven, Belgium), with concentrations ranging from 3.9 × 10^−4^ to 1024 μg/mL. Isolates were suspended in 0.9% NaCl (Merck KGaA^®^, Darmstadt, Germany) to obtain a final concentration of approximately 1–2 × 10^8^ CFU/mL. Subsequently, 50 μL of each suspension was added to 10 mL of MHCA broth and inoculated into the wells of the microplate, producing a final bacterial concentration of 2.5–5 × 10^5^ CFU/mL per well. This concentration was confirmed through colony counting after serial dilution, plating and incubation at 36 °C (±1 °C) for 24 h [19].

Results were recorded after the incubation of the plates for 16 to 20 h at 36 °C (±1 °C). The MIC was defined as the lowest antibiotic concentration that visually inhibited bacterial multiplication [20]. Positive (bacterial suspension in MHCA broth) and negative (MHCA broth and antibiotic) controls were also included. Assays were performed in triplicate on three independent days to ensure the reproducibility of the results.

#### 2.2.2. Bacterial Growth Determination

According to Pont et al. [8], persistence induction should be performed during the mid-exponential phase, since bacteria are more susceptible to antimicrobials at this stage. To determine the approximate time required for each isolate to reach the mid-exponential phase and the optical density (OD) of the corresponding bacterial suspensions, bacterial growth curves were established for all 40 isolates under study.

The initial steps of bacterial preparation followed the protocol previously established by Pont et al. [8]. Freshly grown bacterial suspensions on Brain Heart Infusion (BHI) agar (VWR^®^ International, Leuven, Belgium) were used to inoculate 5 mL of Mueller–Hinto (MH) broth (Oxoid Limited^®^, Hampshire, UK), which was then incubated at 36 °C (±1 °C) for 16–18 h. Afterwards, 100 μL of these cultures were centrifuged and the resulting pellet was washed twice in PBS (VWR^®^ International, Leuven, Belgium), resuspended in the same volume of fresh MH broth, and transferred into 9.9 mL of uninoculated MH broth. Then, 100 μL of this freshly inoculated broth were added to three wells of a 96-well plate (VWR^®^ International, Leuven, Belgium) and optical density readings at 600 nm were recorded hourly for 24 h using a microplate reader (FLUOstar Omega OPTIMA, BMGLABTECH, Ortenberg, Germany).

Three negative control wells, composed of uninoculated MHCA broth, were also included, and their OD values were subtracted from those of each test well at each time point.

All assays were performed in triplicate on three independent days to guarantee the results’ reproducibility.

#### 2.2.3. Persistence Induction

Persistence induction was performed following a previously described protocol [8]. Bacterial preparation for persistence induction was carried out as described in the previous section. After overnight incubation and washing, 10 mL bacterial suspensions in MH broth were further incubated until an OD of 0.3 was reached. Subsequently, 1 mL of ciprofloxacin was added to achieve a final concentration of 10× the MIC. Due to the maximum solubility of ciprofloxacin, four isolates (ED4, ED5, ED9, EN41) were treated with the antibiotic at 2.2× the MIC value, one (EN3) with 4.5× the MIC and two (EN31, EN32) with 8.9× the MIC, all corresponding to a concentration of 2500 μg/mL.

At 0, 24 and 48 h, 100 μL aliquots were taken from each suspension, serially diluted in 0.9% NaCl in duplicate, and 20 μL were spotted onto Tryptic Soy Agar (TSA) (VWR^®^ International, Leuven, Belgium). After a 48 h incubation at 36 °C (±1 °C), colonies within each drop were counted, and the bacterial concentrations (CFU/mL) at each time point were determined using the mean count value between duplicates. This allowed for the construction of the killing curves that allowed for the observation of ciprofloxacin’s bactericidal action and possible biphasic behaviors.

Growth (bacteria without antibiotic) and negative controls (MH broth with antibiotic) were also included in the assays.

#### 2.2.4. Persistence Confirmation

After exposure to ciprofloxacin for 48 h, three colonies from each replicate, collected from the TSA plates used for CFU determination, were inoculated in fresh BHI agar and incubated for 24 h at 36 °C (±1 °C). Subsequently, susceptibility testing was repeated using the recovered cells. Ciprofloxacin MIC values were determined using broth microdilution, while susceptibility to thirteen previously tested antibiotics [21] was reassessed using the disk diffusion method, following CLSI guidelines [17,18,19,22]. The antibiotics tested were as follows: ampicillin (10 μg), amoxicillin and clavulanic acid (30 μg), ciprofloxacin (5 μg), levofloxacin (5 μg), tetracycline (30 μg), doxycycline (30 μg), erythromycin (15 μg), chloramphenicol (30 μg), linezolid (30 μg), vancomycin (30 μg), teicoplanin (30 μg) and high-level gentamicin (120 μg) and streptomycin (300 μg) (Oxoid, Hampshire, UK). This new susceptibility testing aimed to confirm that the observed tolerance to ciprofloxacin was not due to the acquisition of new resistance determinants, but possibly to cells entering a low-metabolic, non-replicative state, as is typical for persisters [4,5]. Additionally, an aliquot from the 48 h ciprofloxacin-treated suspensions was used to establish new growth curves, following the protocol previously described, to assess if the growth of the resulting cells was similar to the one of the original isolates. New growth curves were also performed for the recuperated isolates selected for WGS, after a passage in TSA, for additional confirmation that both isolates displayed growth curves comparable to that of their respective original isolate.

#### 2.2.5. Whole-Genome Sequencing (WGS)

To better understand the mechanisms associated with persistence development, WGS of non-exposed isolates was performed, including one with the capacity of persister development (EH4), and one without this ability (EH1), allowing comparison of the genetic composition of both isolates. Additionally, the three ciprofloxacin-exposed replicates of each isolate were also analyzed through WGS, to determine the occurrence of any genetic mutations due to antibiotic exposure. As such, after 48 h of exposure to ciprofloxacin, suspensions from both exposed and non-exposed cells, submitted to the same growth conditions, were used for DNA extraction, purification and WGS.

Initial DNA extraction and purification were performed using the GES protocol [23]. DNA purity was assessed with the NanoDrop ND-1000 spectrophotometer, while quantification was done using the Qubit fluorometer (v1.01). Following this step, end-repair and dA-tailing were accomplished (New England BioLabs, Ipswich, MA, USA), followed by further purification using Agencourt AMPure XP Beads (Beckman Coulter, Amersham, UK).

Libraries were prepared from 200 fmol input DNA using the Sequencing Nave Barcoding Kit 24 V14 (SQK-NBD114.24, Oxford Nanopore Technologies, Oxford, UK), according to the manufacturer’s instructions. Sequencing was carried out on FLO-PRO114M flow cells using the PromethION 2 platform, with MinKNOW (v22.12.4).

For all genomes, read quality was assessed with NanoPlot (v1.42.0) [24] and contamination screening was performed with Kraken2 (v2.1.6) [25] against the NCBI database (2024 release).

For non-exposed isolates, de novo assembly was performed using the Hybracter pipeline (v0.7.3) [26], with a quality assessment using QUAST (v5.2.0) [27] and Kraken2 (v2.1.6) [25] (Supplementary Materials File S1). Contigs obtained from the assemblies were subjected to BLASTn (v2.17.0) analysis for taxonomic identification. For EH4.2, multiple contigs were found to exhibit homology to the chromosome of *Enterococcus faecalis*. Genome comparisons were visualized using BRIG (v0.95) [28], which indicated that the chromosomal sequence could be reconstructed by combining these contigs. This suggested that the initial assembly of EH4.2 was fragmented. Therefore, these contigs were concatenated and treated as a single chromosomal sequence in all subsequent analyses.

Regarding the exposed isolates, filtered reads were aligned to the corresponding ancestral reference genome (EH1 and EH4) using Minimap2 (v2.30) [29], and sorted and indexed with Samtools (v1.20) [30]. Single-nucleotide variants (SNVs) and small indels were identified using Longshot (v0.4.5) [31] and normalized and indexed with Bcftools (v1.20) [30], assuming haploid ploidy.

For further genome comparisons, genome annotation was performed using Prokka (v1.14.6) [32], the pangenome was determined with Roary (v3.13.0) [33] and compared using Geneious Prime^®^ 2026.0.1. Each protein sequence identified as being present in one genome but absent in the other was first analyzed using BLASTn (v2.17.0) [34] using the genome lacking the protein as the subject sequence to confirm its absence. Subsequently, BLASTx (v2.17.0) [34] was used for protein identification or, in the case of hypothetical proteins, to attempt identification through similarity.

Species assignment was confirmed using FastANI (v1.34) [35], based on comparisons against both isolates under study and all *Enterococcus* reference genomes available at NCBI (accessed on 1 July 2025). Multilocus sequence typing (MLST) was performed with PubMLST (https://pubmlst.org/, accessed on 1 July 2025) [36], using the *E. faecalis* scheme.

## 3. Results

### 3.1. Induction and Phenotypic Confirmation of Bacterial Persisters

**Figure 1:**
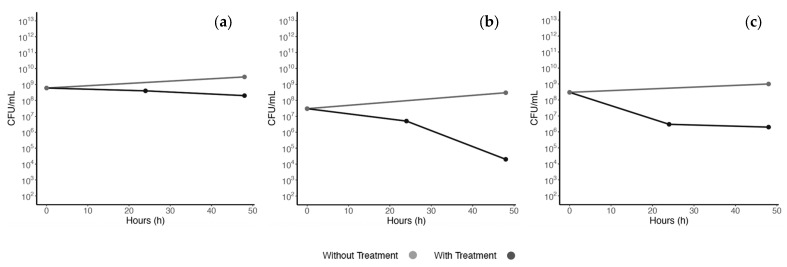
Time–kill assay graphs illustrating three distinct bacterial responses to ciprofloxacin, and respective growth controls: (**a**) an isolate showing no significant decrease in bacterial concentration; (**b**) an isolate exhibiting a progressive decrease in concentration, most pronounced at 48 h (EH1); (**c**) an isolate showing a biphasic killing curve, with an initial abrupt reduction in bacterial concentration (>2 orders of magnitude), followed by a plateau, characteristic of persister cells (EH4).

During the 48 h of exposure to ciprofloxacin, the bacteria presented three different time–kill curve patterns, as represented in Figure 1.

Most isolates (n = 33) showed a limited bactericidal response, with reductions of ≤1 order of magnitude after 48 h, which was not considered biologically meaningful. Regarding the remaining seven bacteria, only one human isolate, identified as *E. faecalis* (EH4), displayed a rapid decline in bacterial concentration (≥2 orders of magnitude) in the first 24 h, followed by a plateau in cell death over the subsequent 24 h. EH4 also did not present any changes to its resistance profile, including in the MIC value for ciprofloxacin. Based on these observations, cells from each replicate of this isolate were collected after the 48 h of contact with ciprofloxacin and sent for WGS (EH4.1, EH4.2 and EH4.3). Additionally, a control (EH4), grown under the same conditions but without antibiotic exposure, was also sent for WGS analysis, serving as a negative control.

Susceptibility testing confirmed that both the presumed persister and control maintained the same resistance profile, including identical MIC values for ciprofloxacin.

Growth curves were performed for the presumptive persister and, for comparison purposes, for one non-persister (EH1), which was also sent for WGS. This isolate was chosen as it belonged to the same group as EH4 and was also submitted to 10× MIC of ciprofloxacin. Its growth curve also presented a significant reduction in bacterial concentration after 48 h (Figure 1b), excluding any possibility of persister formation under these specific experimental conditions, contrasting to what was seen for isolate EH4 (Figure 1c). This reduction also indicates susceptibility at 10× the MIC, which was observed in this isolate but not in most others, and is crucial for the detection of persister formation.

**Figure 2:**
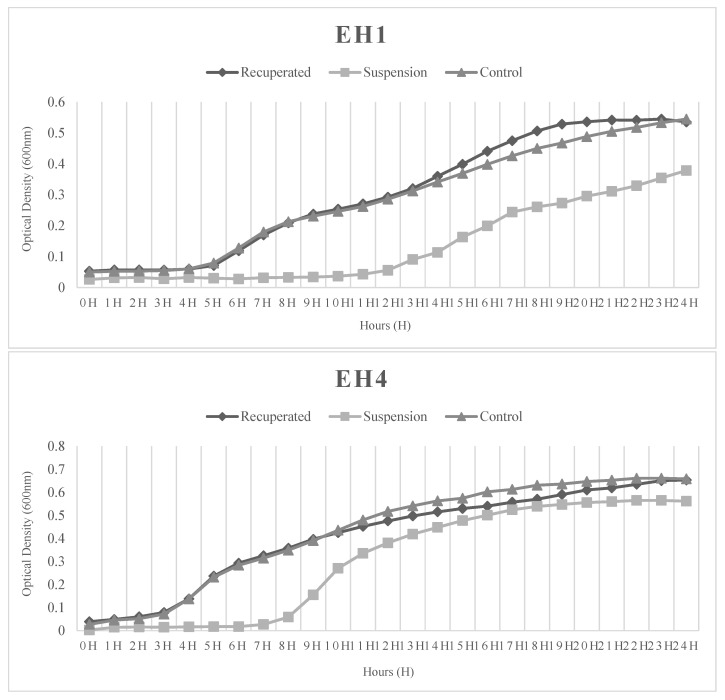
Growth curves for one non-persister (EH1) and one persister (EH4) isolate. Recuperated: Isolate after persistence induction and one single passage through TSA; suspension: ciprofloxacin-treated isolate after persistence induction obtained directly from the test suspension; control: original isolate.

As shown in Figure 2, both the EH1 and EH4 recuperated isolates presented the same growth curve as the original isolates (control). As demonstrated by the suspension growth curve, obtained after 48 h of antibiotic exposure, both isolates resumed growth following a prolonged lag phase. However, after this phase, EH4 recovered a growth capacity comparable to that of the control isolate, whereas EH1 did not.

### 3.2. Genomic Characterization of a Bacterial Persister by Whole-Genome Sequencing

De novo assembly produced one similar contig common to all strains, which was confirmed to be on the chromosome when aligned to a reference genome (NCBI Reference Sequence NZ_KB944666.1). However, in all non-persister strains (EH1, EH1.1, EH1.2 and EH1.3) and in one persister replicate (EH4.2), this alignment revealed a chromosomal gap that corresponded to an additional contig in theses samples. Additionally, EH4.2 also presented a total of seven contigs, whereas the other replicates (EH4.1 and EH4.3) and the original isolate (EH4) each contained three contigs. This discrepancy was interpreted as a misassembly, and the sequences were subsequently merged, resulting in an equal number of genetic elements. In addition to the chromosome, EH1 strains harbored one plasmid (GenBank CP175054.1), while EH4 strains presented two plasmids (GenBank LR962420.1 and CP143581.1). Moreover, part of the CP175054 plasmid was not recovered in the EH1 assemblies, which may be due to assembly-related limitations rather than genuine plasmid loss.

SNVs comparisons between the control (EH4) and the corresponding ciprofloxacin-exposed replicates (EH4.1, EH4.2, EH4.3) revealed a single insertion at CP143581:61648 (C > CT), located in the position 50328 of the reference plasmid CP143581, situated within the adhesin *asa1* gene. In the reference plasmid, this locus consists of a CAT{7} motif. The non-exposed isolate (EH4) carried a C-T{7} sequence, and the exposed isolates carried a CTT{7} sequence.

FastANI analysis confirmed that the assembled genomes shared 98.8–98.9% identity with the *E. faecalis* reference genome used. Additionally, this analysis showed a 99.8% similarity between EH1 and EH4 (in both query-reference directions) (Table 1). All isolates were assigned to sequence type ST-6.

Comparing the persistent and non-persistent isolates, Roary initially identified a total of 260 proteins present in EH1 but absent in EH4, and 295 proteins in the reciprocal comparison. This list can be found in Table S1. Manual curation using Blast allowed the correction of misidentified comparisons, reducing the number of different proteins to approximately 232 and 255, respectively. Additional identifications were also possible using the same program, some based on homology with other microorganisms. Nevertheless, in EH1, approximately 62 proteins were still annotated as hypothetical or as having a domain of unknown function (DUF), while in EH4 this number was approximately 122 proteins. A summary of the main differences regarding EH1 and EH2 can be found in Table 2.

EH4 contained a significant number of putative phage-associated proteins (n = 33), which were absent in EH1. Conversely, EH1 seemed to harbor a greater number of genes associated with antibiotic resistance, including two copies of *erm(B)*, an additional copy of *aadKI*, and the *aphA*, *sat4*, and *cat* genes. In contrast, EH4 carried more genes potentially associated with virulence and biofilm formation, such as a collagen-like protein gene and two additional copies of *asa1*. Both isolates were found to harbor genes that presented homology to the *gyrA*, *gyrB*, *parC*, and *parE* genes, which are associated with ciprofloxacin resistance. Moreover, within the final protein list, several proteins potentially involved in persister development and survival were identified as of interest. These genes included multiple TA systems and a cold-shock protein (Csp), as presented in Table 3. Additionally, some differences regarding the tyrosine-type recombinase/integrase systems were also found. EH4 seems to have two additional copies of tyrosine-type recombinases/integrases, one of which possibly corresponds to XerC, and which seem to have no homology with those found in EH1. Numerous differences were detected among other proteins associated with DNA replication, recombination and repair, although none could be clearly associated with persister formation.

Interestingly, both plasmids present in the EH4 also encoded conjugal transfer proteins.

It is also noteworthy that, while the identified TA system proteins showed no homology in the genome of EH1, the additional cold-shock protein Csp found in EH4’s genome seems to be a supplementary copy of this gene. So, while EH1 presents a total of four cold-shock or cold-shock-like proteins, all homologous to those in EH4, the additional copy found in EH4 differs slightly from the EH1 proteins, sharing 89% identity (159/179) with 100% coverage and no gaps (0/179).

Additional differences were also found for proteins involved in transcription regulation, mobile genetic elements, and other functional categories.

## 4. Discussion

*Enterococcus* spp. is a diverse genus with well described intrinsic and extrinsic resistances to many antibiotics. While this seems especially true for *E. faecium*, *E. faecalis* is also frequently associated with infection, which is attributed to their vast assortment of virulence genes and biofilm formation capacity [2]. Beyond resistance and virulence, enterococci usually present a high adaptability to external stressors, which makes them great candidates for persistence studies.

In this study, a collection of 40 enterococci from diverse origins, including human and veterinary isolates, was exposed to high concentrations of ciprofloxacin to uncover potential persistence formation, following a protocol similar to the one described by Pont et al. [8]. Due to the limited studies regarding persistence in *Enterococcus* spp., adherence to established protocols is crucial, as, although persistence induction may be achieved by different stressors, they may activate distinct mechanisms [7,9]. However, as concluded by the results obtained in this study, and possibly due to the resistance presented by this genus, ciprofloxacin at a concentration of 10× the MIC (or lower for some isolates) was not entirely efficient in inducing persistence, with most isolates showing no significant reduction in bacterial concentration upon exposure to these antibiotic concentrations. Nevertheless, one potential persister strain was identified in time–kill assays, exhibiting a biphasic killing curve similar to those reported in previous studies [7], including for *Enterococcus* spp. [6,8]. Additionally, and in accordance with previous studies, no development of a higher degree of resistance or cross-resistance to ciprofloxacin or other antibiotics was observed in this strain. Its growth curve also presented an extended lag phase, but recovered their multiplication ability within a few hours, in accordance with what was described by Pont and colleagues [8]. Notably, the non-persistent isolate (EH1) also represented an example of antibiotic tolerance in *Enterococcus* spp., where some cells survive despite exposure to high antibiotic concentrations [4,5]. However, the observation of both the growth curves suggests that this re-growth ability was more limited in the non-persistent isolate. Regardless, both isolates serve as examples of how bacteria can survive under extreme antibiotic stress, potentially contributing to prolonged infections.

No genetic alterations were detected in specific antibiotic resistance sequences that could explain sudden resistance development, which is consistent with what is expected regarding persister isolates [8]. However, it is interesting to note that both isolates presented genes associated with ciprofloxacin resistance, which re-enforces the concept that persistence does not seem to be directly correlated with resistance.

Also, the only SNP insertion identified was in the *asa1* gene, which encodes a group of aggregation substances in *Enterococcus* spp. related to cell adhesion and immune-system evasion, which are important factors in infection development [37,38]. As *asa1* has only been infrequently linked to biofilm formation [39], the insertion identified in this study could be biologically non-significant, especially considering that two similar additional copies of this gene could be found within this isolate’s genome, although one of them is of smaller size. Further studies are needed to clarify the importance of this single alteration. Nevertheless, it is important to note that biofilms are commonly associated with persistence. A study by Kaviar et al. [11] supports this association, as it reports an overexpression of biofilm-associated genes (*esp*, *agg*, and *gelE*) in biofilm-derived persister cells in comparison to their non-persistent counterparts.

FastANI retains only fragments with an identity above a certain threshold (close to 80% or higher), in order to calculate ANI across homologous regions [35]. This means that fragments that do not align, as well as those aligning below this threshold, are excluded. These fragments likely correspond to absent or highly divergent regions. These excluded regions, together with sequence variation (e.g., indels) and differences in gene prediction, likely contribute to the ~232–255 unique proteins identified by Roary. Therefore, despite these differences, ANI values indicate that the observed variation reflects normal strain-level diversity rather than a substantial genomic divergence.

Candidate genes associated with persistence were analyzed not only based on their presence/absence, but also considering their functional relevance and prior evidence from the literature supporting their role in persister formation. TA systems, especially type II systems, have frequently been linked with persistence in many bacterial species [9], and are usually composed of a toxin and its corresponding antitoxin, where the deleterious effects of the toxin are counteracted by direct binding of a protein antitoxin [40]. In persistence formation, systems such as *relAB* and *mazEF* are responsible for stress-induced protease activity that degrades the antitoxin, leading to toxin accumulation [9,41,42,43]. Christensen et al. [43] and Pedersen et al. [42] showed that, in *Escherichia coli*, the accumulation of these toxins was correlated with translation inhibition and, in the case of *mazEF*, possibly with replication arrest, consequently promoting persistence. In comparison with the EH1 isolate, EH4 seems to have additional proteins, potentially associated with both the *relAB* and *mazEF* systems, which may result in a higher expression of these systems in the presence of stress factors. Additionally, MazF overexpression has been associated with cell protection to ciprofloxacin [44]. Nevertheless, it is important to note that the absence of additional TA genes could also lead to persister formation, due to a possible redundancy found among these systems [41].

Members of the TA family associated with tolerance to antibiotics and located in plasmids have been previously described in microorganisms such as *E. coli* and *Salmonella* spp. [45]. Additionally, the localization of these additional copies on a putative conjugative plasmid could facilitate their horizontal dissemination.

EH4 also has an additional copy of a cold-shock protein, which have also been shown to be controlled by the TA system, the *mqsRA* in *E. coli*, and have been previously possibly associated with persistence formation in this species [46], and more importantly in *E. faecium* (CspA) [8]. An additional copy of XerC, a recombinase that plays an important role in DNA repair after ciprofloxacin-induced damage in *Staphylococcus aureus* [47], was also found.

The observed differences in toxin–antitoxin systems, cold-shock proteins and tyrosine-type recombinases are consistent with previous reports linking these elements to stress responses and persistence; however, given the limited number of isolates analyzed, they should be interpreted as candidate features rather than definitive persistence-specific mechanisms.

In conclusion, genes related to TA systems, cold-shock proteins and DNA recombination and repair proteins, such as XerC, emerged as plausible candidates in the persister isolate and may represent relevant targets for future functional studies addressing ciprofloxacin-induced persistence in *Enterococcus* spp. In addition, conjugation studies could allow us to understand if the detected TA system genes located in plasmids could contribute to persister formation.

One of the limitations of this study is the fact that WGS, while enabling the identification of potential genes of interest that could be the focus of further investigation, does not provide information on gene expression levels. This aspect should be addressed in future studies using RNA-sequencing. The relatively high number of detected proteins with unknown function, together with the fact that persistence was observed in only a single isolate, also limits the amount of information that can be extracted from this and other subsequent analysis. The testing of additional isolates and experimental conditions would therefore be an important next step.

Additionally, solubility constraints lead to seven isolates being tested at concentrations below the recommended threshold concentration for persistence induction in *Enterococcus* spp., which may have led to an underestimation of persister frequency and could partially explain the low number of persistent isolates obtained.

## 5. Conclusions

To the best of our knowledge, this is the first study that attempted to induce persistence in a large collection of *Enterococcus* isolates, including strains from veterinary origin. Although a single human-derived *E. faecalis* isolate was successfully induced into a persister state, ciprofloxacin at 10× the MIC seemed to be insufficient to achieve effective killing in the majority of the isolates, indicating that higher concentrations or different antibiotics should be explored in future studies. Importantly, these findings demonstrate that *Enterococcus* spp. can survive exposure to high antibiotic concentrations through mechanisms consistent with tolerance or persistence. However, validation of the role of these genes in persistence using methodologies such as RNA-sequencing is a crucial next step in this study.

## Figures and Tables

**Table 1:** FastANI values of the original EH1 and EH4 isolates.

Genome 1	Genome 2	ANI (%)	Aligned Fragments	Total Fragments	Aligned (%)	Fragments Not Aligned
EH1	EH4	99.8	1038	1113	93.26	75
EH4	EH1	99.8	1039	1111	93.52	72

**Table 2:** Approximate number of different proteins present in each isolate, grouped by function.

Associated Putative Function	Present Only in EH1	Present Only in EH4
Antibiotic Resistance	6	-
Virulence	3	6
Toxin–Antitoxin Systems	7	8
Other Functions	154	119
Hypothetical Proteins	62	122
Total	232	255

**Table 3:** Candidate genes associated with persistence in EH4, in comparison with the non-persistent EH1.

Isolate	Prokka ID	Blastx ID ^1^		Location
Toxin–Antitoxin System				
EH1	Antitoxin epsilon	Antitoxin epsilon	95%; 89% (32/36); 91% (33/36); 0% (0/36); 3^−12^	CR
	Antitoxin epsilon	Antitoxin	91%; 100% (61/61); 100% (61/61); 0% (0/61); 3^−25^	CR
	Toxin zeta	Zeta toxin family protein (partial)	92%; 100% (122/122); 100% (122/122); 0% (0/122); 4^−85^	CR
	Hypothetical protein	Type II TA system RelB /DinJ family antitoxin.	98%; 96% (53/55); 100% (55/55); 0% (0/55); 1^−30^	CR
	Hypothetical protein	Type II TA system RnlB family antitoxin.	72%; 51% (43/84); 72% (61/84); 0% (0/115); 2^−21^	CR
	Hypothetical protein	Type III TA system ToxN/AbiQ family toxin	99%; 91% (69/76); 97% (74/76); 0% (0/76); 5^−44^ *	CR
	Hypothetical protein	Type III TA system ToxN/AbiQ family toxin	99%; 100% (101/101); 100% (101/101); 0% (0/101); 1^−64^ *	CR
EH4	Hypothetical protein	MazG-like family protein	99%; 90% (89/99); 95% (95/99); 0% (0/99); 8^−57^	CR
	Hypothetical protein	TA system, antitoxin component, Xre domain protein	99%; 90% (89/99); 95% (95/99); 0% (0/99); 8^−57^	CR
	Endoribonuclease PemK	Type II TA system PemK/MazF family toxin	96%; 100% (116/116); 100% (116/116); 0% (0/116); 2^−79^	P1
	Antitoxin MazE	AbrB/MazE/SpoVT family DNA-binding domain-containing protein	99%; 100% (87/87); 100% (87/87); 0% (0/87); 1^−55^	P1
	Hypothetical protein	Type II TA system RelB family antitoxin	99%; 100% (77/77); 100% (77/77); 0% (0/77); 5^−32^	P1
	Hypothetical protein	Type II TA system RelE family toxin	99%; 94% (84/89); 98% (88/89); 0% (0/89); 8^−57^	P1
	Hypothetical protein	Type II TA system PemK/MazF family toxin	99%; 99% (107/108); 99% (107/108); 0% (0/108); 1^−72^	P2
	Hypothetical protein	AbrB/MazE/SpoVT family DNA-binding domain-containing protein	99%; 99% (76/77); 98% (76/77); 0% (0/77); 5^−47^	P2
Cold-Shock Proteins				
EH4	Cold-shock protein 1	Cold-shock protein	99%; 96% (119/124); 96% (120/124); 0% (0/124); 5^−81^	CR
Tyrosine-Type Recombinase/Integrase				
EH1	Tyrosine recombinase XerD	Tyrosine-type recombinase/integrase	98%; 98% (391/398); 99% (395/398); 0% (0/398); 0	CR
EH4	Tyrosine recombinase XerC	Tyrosine-type recombinase/integrase	100%; 78% (303/387); 88% (344/387); 0% (3/387); 0	CR
	Putative prophage phiRv2 integrase	Tyrosine-type recombinase/integrase	100%; 99% (373/376); 99% (374/376); 0% (0/376); 0	CR

^1^ Cover; Identities; Gaps; E value; * Identification made through homology with *Peptostreptococcus olsenii*; TA: Toxin–Antitoxin; CR: Chromosome, P1: Plasmid LR962420, P2: Plasmid CP143581.

## Data Availability

The raw data supporting the conclusions of this article will be made available by the authors on request.

## Supplementary Materials

The following supporting information can be downloaded at https://www.mdpi.com/article/10.3390/microorganisms14040921/s1, Supplementary File S1: Standard assembly metrics and QUAST results; Table S1: Gene comparison between the non-persister (EH1) and persister (EH4) isolates.

## Abbreviations

The following abbreviations are used in this manuscript:

BHI	Brain Heart Infusion
CFU	Colony-Forming Units
CSP	Cold-Shock Protein
DUF	Domain of Unknown Function
GES	Guanidinium Thiocyanate–EDTA–Sarkosyl
HAIs	Hospital-Acquired Infections
HCl	Hydrochloric Acid
MH	Mueller–Hinton
MHCA	Mueller–Hinton Cation Adjusted
MIC	Minimum Inhibitory Concentration
OD	Optical Density
SNVs	Single Nucleotide Variations
TA	Toxin–Antitoxin
TSA	Tryptic Soy Agar
WGS	Whole-Genome Sequencing
