# Supplementary material for: Candidate Genomic Features Associated with Persistence in Enterococcus spp
Source: Microorganisms. 2026 Apr 19;14(4):921. doi: 10.3390/microorganisms14040921 (PMC13119490; doi:10.3390/microorganisms14040921)
Supplement: Supplementary file 1 [file microorganisms-14-00921-s001.zip › Supplementary File S1/QUAST/EH1.1/report.pdf]

Report

	EH1.1_final
# contigs (>= 0 bp)	3
# contigs (>= 1000 bp)	3
# contigs (>= 5000 bp)	3
# contigs (>= 10000 bp)	3
# contigs (>= 25000 bp)	2
# contigs (>= 50000 bp)	2
Total length (>= 0 bp)	3346925
Total length (>= 1000 bp)	3346925
Total length (>= 5000 bp)	3346925
Total length (>= 10000 bp)	3346925
Total length (>= 25000 bp)	3327616
Total length (>= 50000 bp)	3327616
# contigs	3
Largest contig	3002769
Total length	3346925
Reference length	2870381
GC (%)	37.09
Reference GC (%)	37.47
N50	3002769
NG50	3002769
N90	324847
NG90	3002769
auN	2725642.2
auNG	3178156.5
L50	1
LG50	1
L90	2
LG90	1
# misassemblies	54
# misassembled contigs	2
Misassembled contigs length	3327616
# local misassemblies	43
# scaffold gap ext. mis.	0
# scaffold gap loc. mis.	0
# unaligned mis. contigs	0
# unaligned contigs	1 + 2 part
Unaligned length	744075
Genome fraction (%)	90.752
Duplication ratio	1.002
# N's per 100 kbp	0.00
# mismatches per 100 kbp	795.91
# indels per 100 kbp	23.98
Largest alignment	383837
Total aligned length	2602688
NA50	68632
NGA50	81794
NA90	-
NGA90	3781
auNA	116150.2
auNGA	135433.5
LA50	12
LGA50	9
LA90	-
LGA90	46

All statistics are based on contigs of size >= 500 bp, unless otherwise noted (e.g., "# contigs (>= 0 bp)" and "Total length (>= 0 bp)" include all contigs).

Misassemblies report

	EH1.1_final
# misassemblies	54
# contig misassemblies	54
# c. relocations	52
# c. translocations	2
# c. inversions	0
# scaffold misassemblies	0
# s. relocations	0
# s. translocations	0
# s. inversions	0
# misassembled contigs	2
Misassembled contigs length	3327616
# local misassemblies	43
# scaffold gap ext. mis.	0
# scaffold gap loc. mis.	0
# unaligned mis. contigs	0
# mismatches	20715
# indels	624
# indels (<= 5 bp)	531
# indels (> 5 bp)	93
Indels length	6294

All statistics are based on contigs of size >= 500 bp, unless otherwise noted (e.g., "# contigs (>= 0 bp)" and "Total length (>= 0 bp)" include all contigs).

Unaligned report

	EH1.1_final
# fully unaligned contigs	1
Fully unaligned length	19309
# partially unaligned contigs	2
Partially unaligned length	724766
# N's	0

All statistics are based on contigs of size >= 500 bp, unless otherwise noted (e.g., "# contigs (>= 0 bp)" and "Total length (>= 0 bp)" include all contigs).

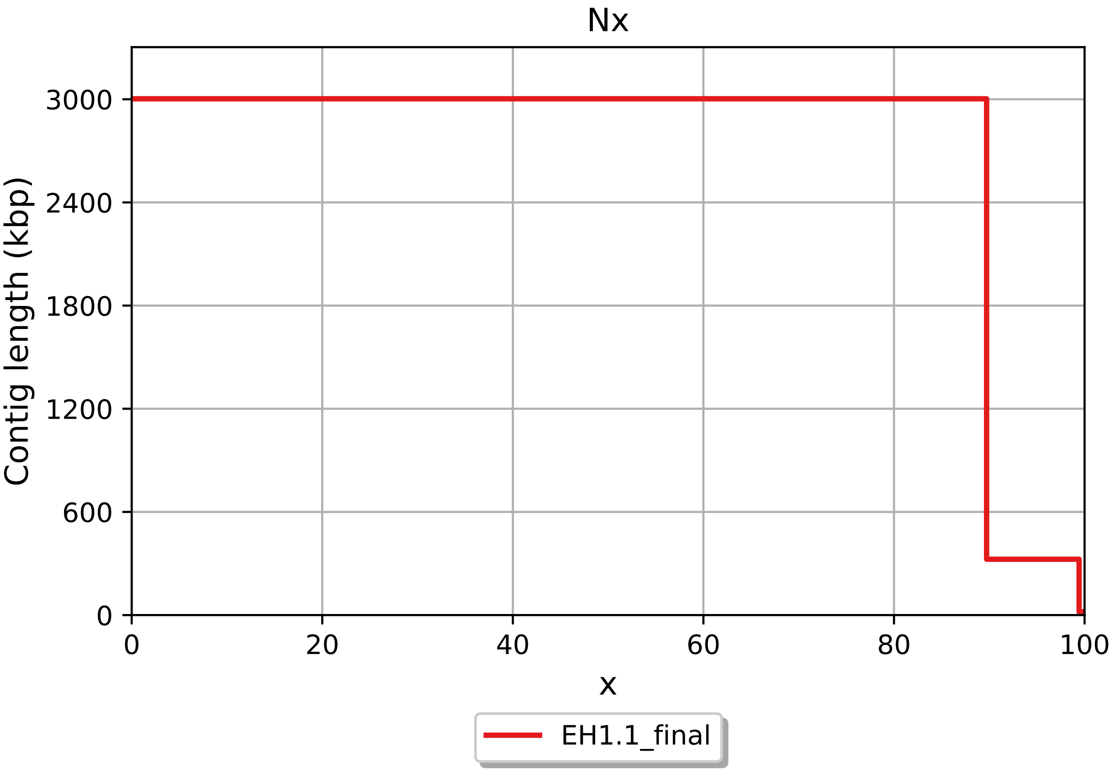

Nx
Contig length (kbp)
3000
2400
1800
1200
600
0
0
20
40
60
80
100
x
EH1.1_final

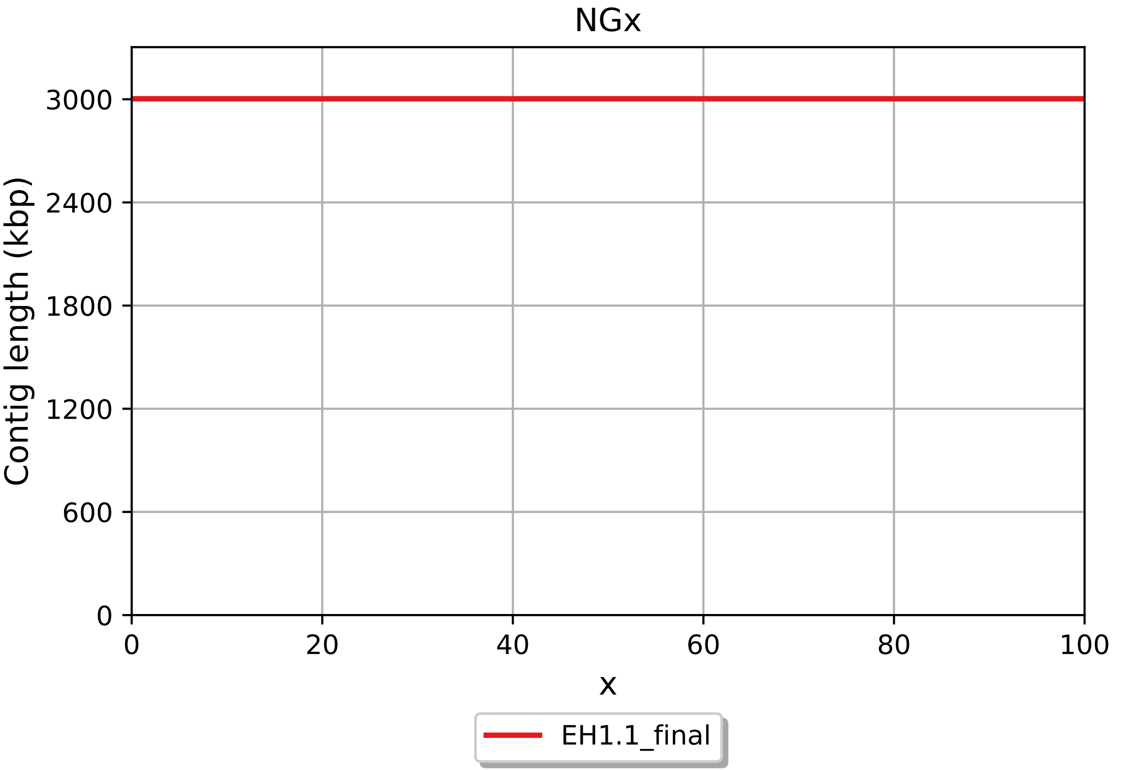
NGx
3000
2400
1800
1200
600
0
Contig length (kbp)
0
20
40
60
80
100
x
EH1.1_final

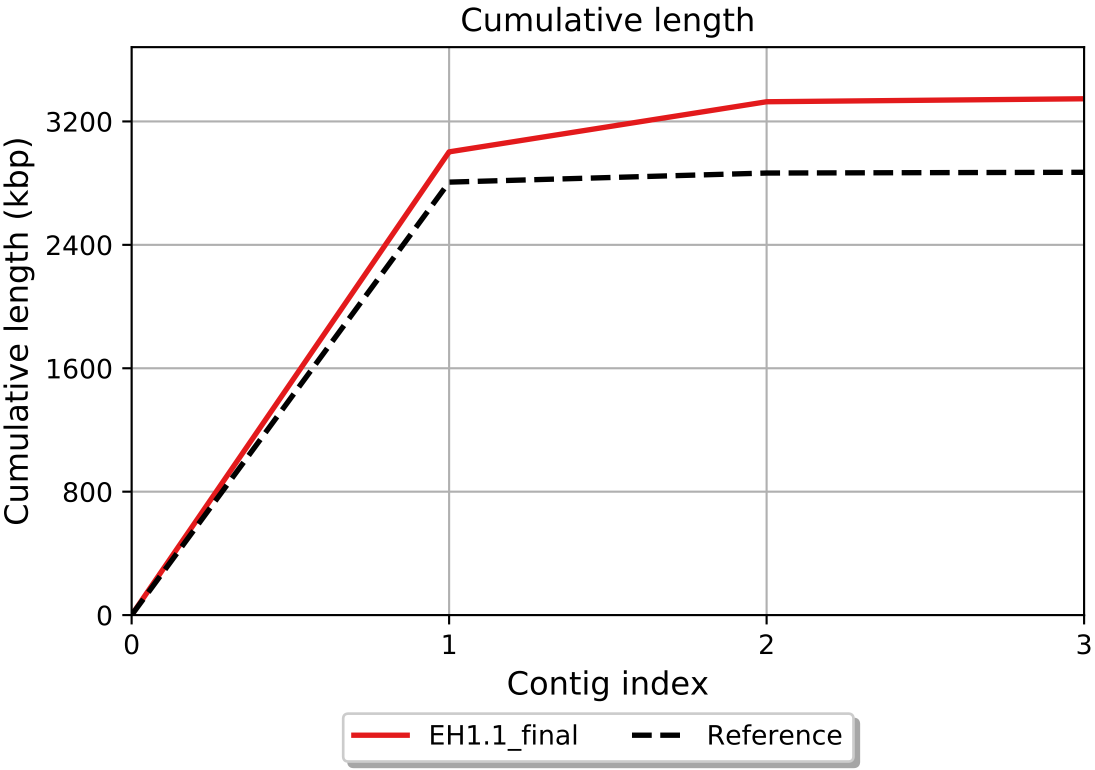

Cumulative length
Cumulative length (kbp)
0
800
1600
2400
3200
0
1
2
3
Contig index
EH1.1_final
Reference

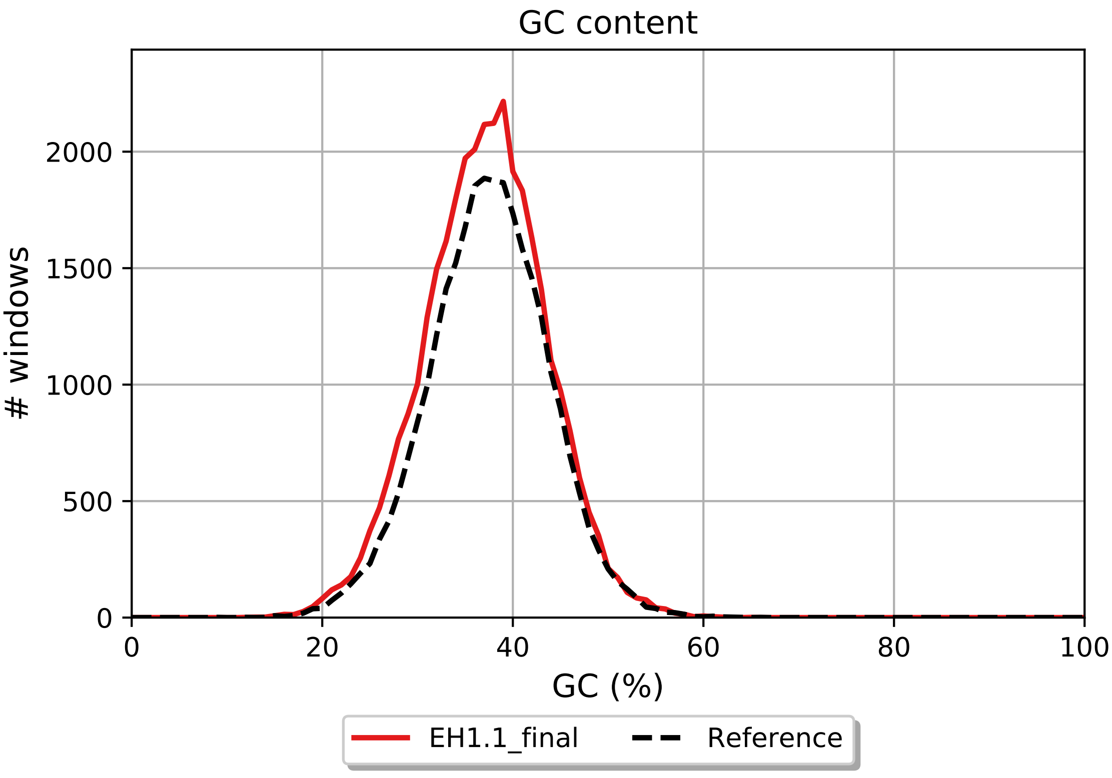
GC content
windows
2000
1500
1000
500
0
0
20
40
60
80
100
GC (%)
EH1.1_final
Reference

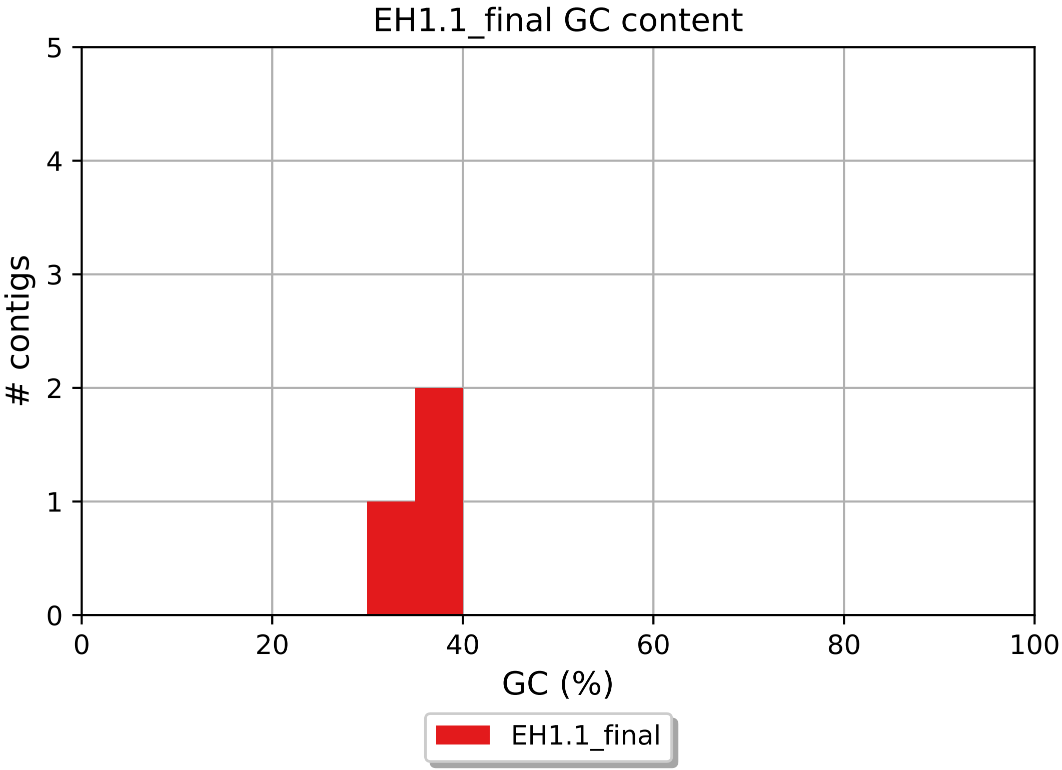

EH1.1_final GC content
contigs
5
4
3
2
1
0
0
20
40
60
80
100
GC (%)
EH1.1_final

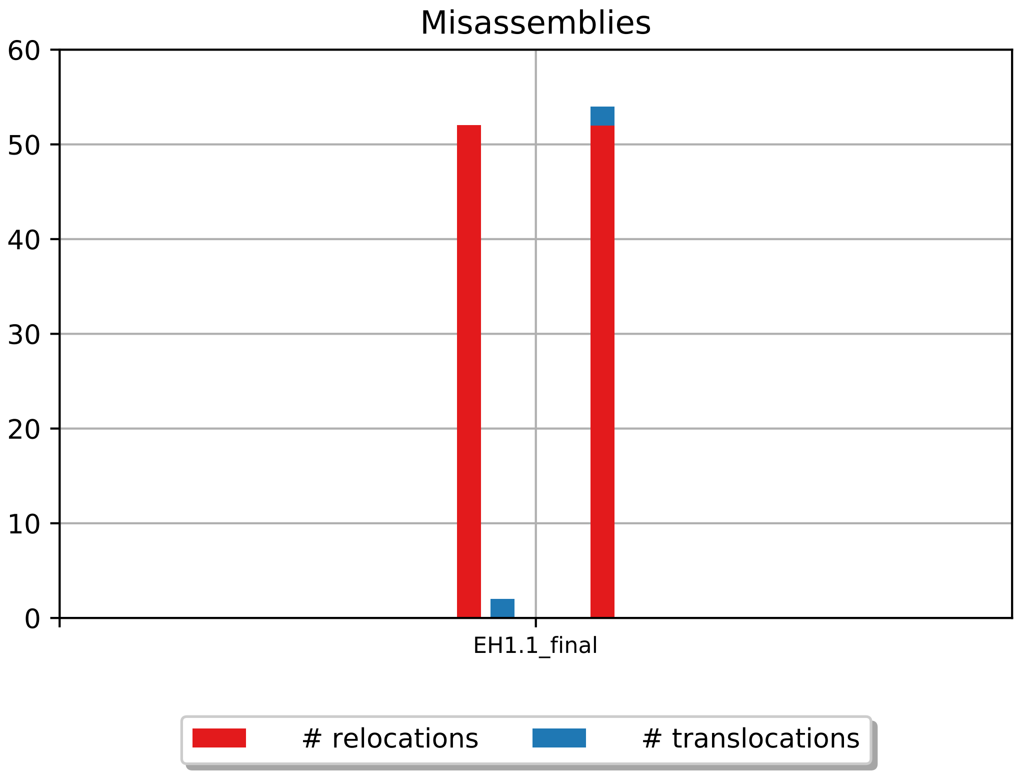
Misassemblies
60
50
40
30
20
10
0
EH1.1_final
relocations
translocations

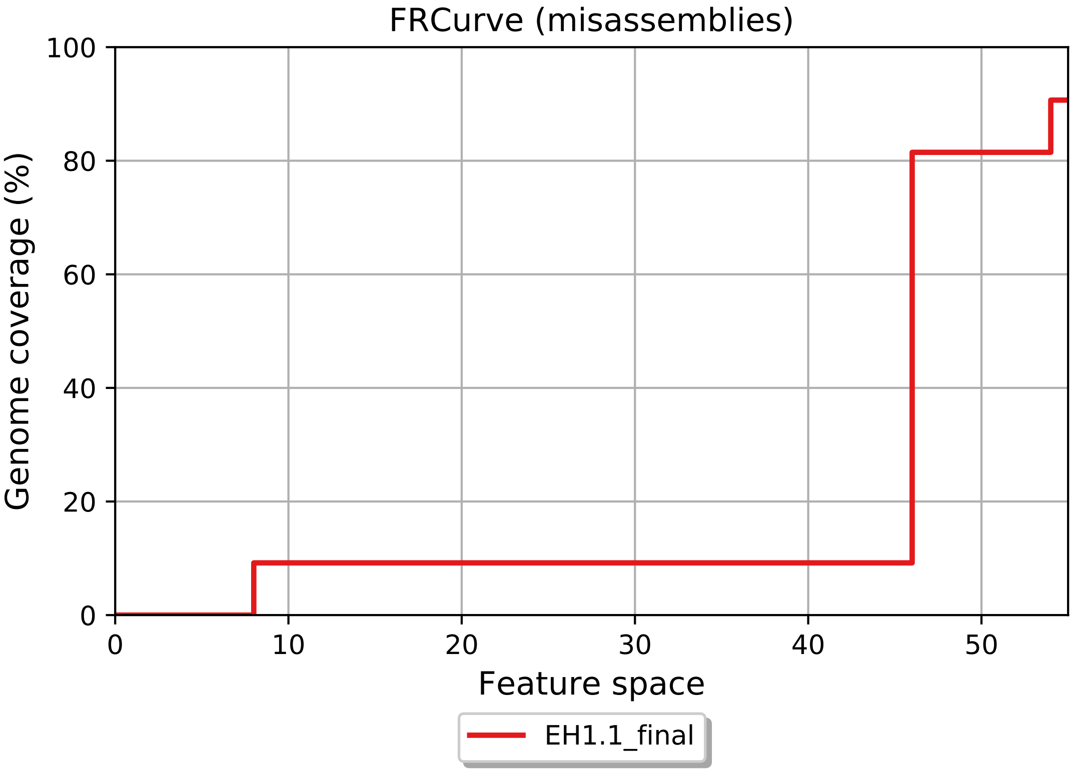

FRCurve (misassemblies)
Genome coverage (%)
0
20
40
60
80
100
0
10
20
30
40
50
Feature space
EH1.1_final

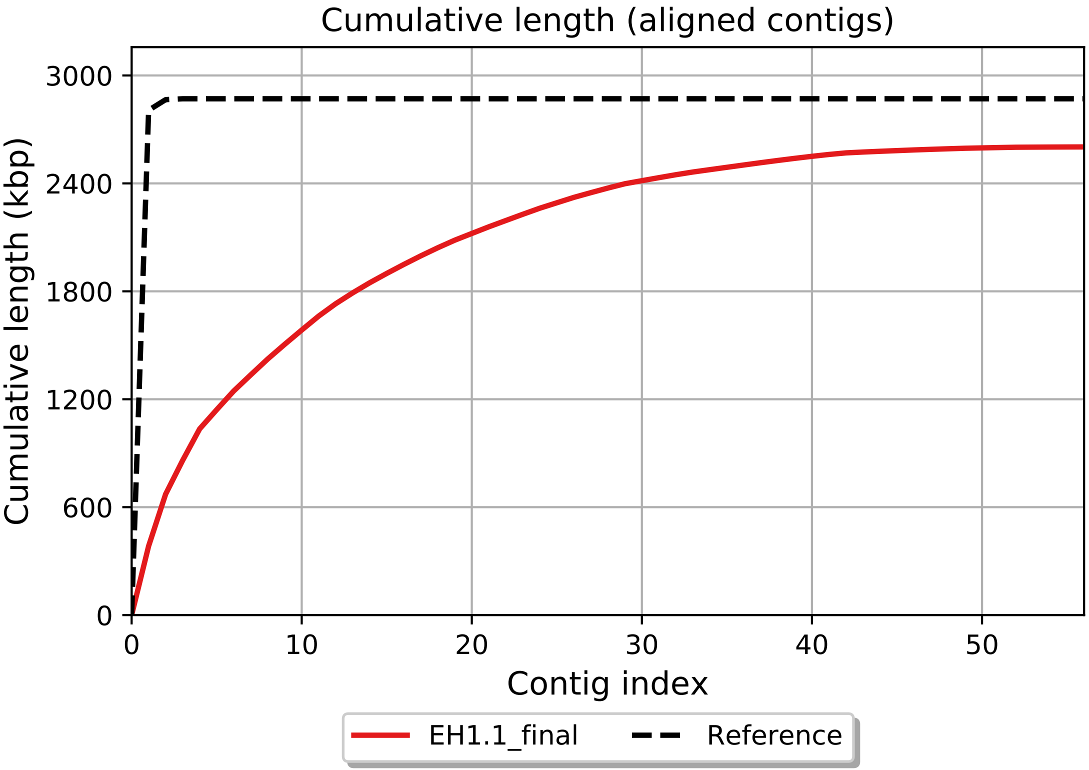
Cumulative length (aligned contigs)
Cumulative length (kbp)
3000
2400
1800
1200
600
0
0
10
20
30
40
50
Contig index
EH1.1_final
Reference

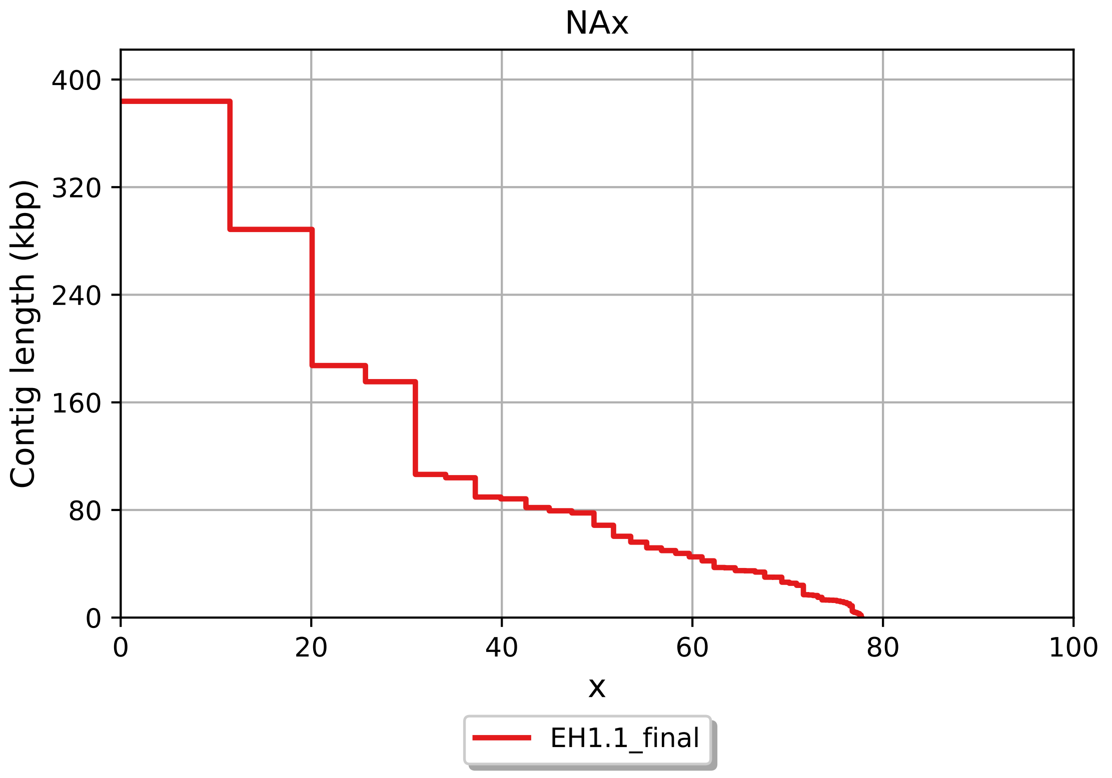
NAx
Contig length (kbp)
400
320
240
160
80
0
0
20
40
60
80
100
x
EH1.1_final

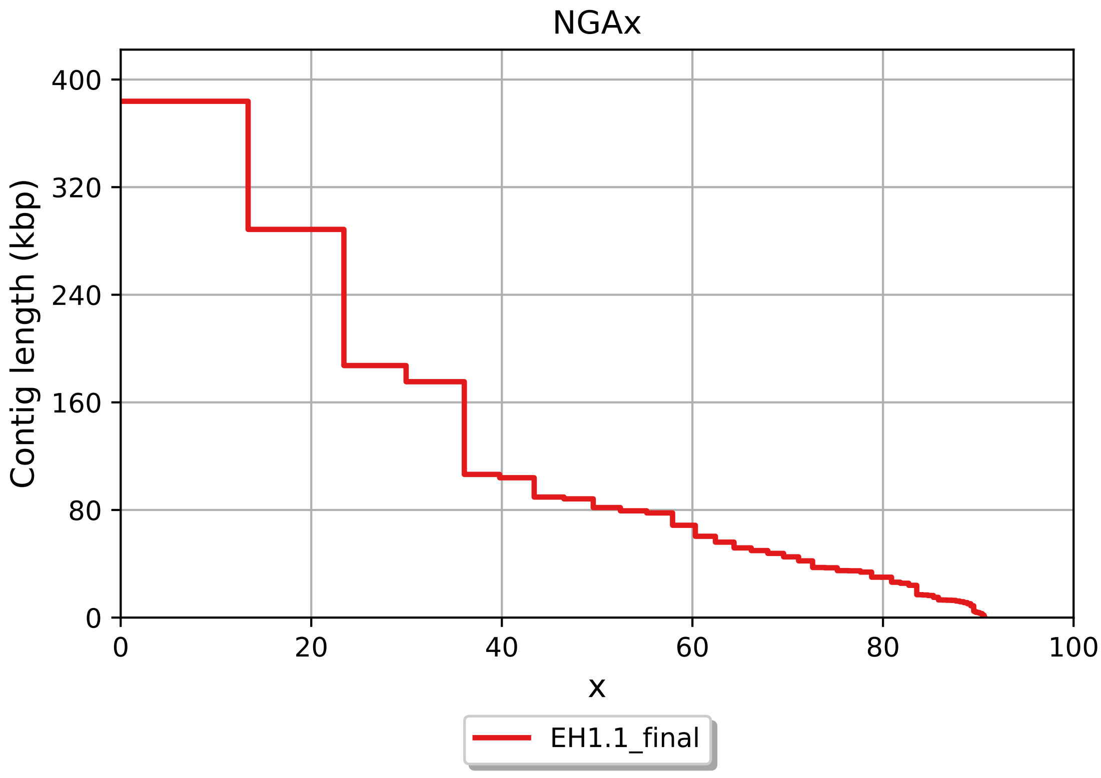

NGAx
Contig length (kbp)
400
320
240
160
80
0
0
20
40
60
80
100
x
EH1.1_final
