# Supplementary material for: Candidate Genomic Features Associated with Persistence in Enterococcus spp
Source: Microorganisms. 2026 Apr 19;14(4):921. doi: 10.3390/microorganisms14040921 (PMC13119490; doi:10.3390/microorganisms14040921)
Supplement: Supplementary file 1 [file microorganisms-14-00921-s001.zip › Supplementary File S1/QUAST/EH1.3/report.pdf]

Report

	EH1.3_final
# contigs (>= 0 bp)	3
# contigs (>= 1000 bp)	3
# contigs (>= 5000 bp)	3
# contigs (>= 10000 bp)	3
# contigs (>= 25000 bp)	2
# contigs (>= 50000 bp)	2
Total length (>= 0 bp)	3351158
Total length (>= 1000 bp)	3351158
Total length (>= 5000 bp)	3351158
Total length (>= 10000 bp)	3351158
Total length (>= 25000 bp)	3331849
Total length (>= 50000 bp)	3331849
# contigs	3
Largest contig	3007001
Total length	3351158
Reference length	2870381
GC (%)	37.09
Reference GC (%)	37.47
N50	3007001
NG50	3007001
N90	324848
NG90	3007001
auN	2729788.9
auNG	3187017.4
L50	1
LG50	1
L90	2
LG90	1
# misassemblies	52
# misassembled contigs	2
Misassembled contigs length	3331849
# local misassemblies	44
# scaffold gap ext. mis.	0
# scaffold gap loc. mis.	0
# unaligned mis. contigs	0
# unaligned contigs	1 + 2 part
Unaligned length	748377
Genome fraction (%)	90.749
Duplication ratio	0.999
# N's per 100 kbp	0.00
# mismatches per 100 kbp	798.03
# indels per 100 kbp	23.96
Largest alignment	383845
Total aligned length	2596508
NA50	68632
NGA50	81676
NA90	-
NGA90	3089
auNA	116353.3
auNGA	135842.0
LA50	12
LGA50	9
LA90	-
LGA90	46

All statistics are based on contigs of size >= 500 bp, unless otherwise noted (e.g., "# contigs (>= 0 bp)" and "Total length (>= 0 bp)" include all contigs).

Misassemblies report

	EH1.3_final
# misassemblies	52
# contig misassemblies	52
# c. relocations	50
# c. translocations	2
# c. inversions	0
# scaffold misassemblies	0
# s. relocations	0
# s. translocations	0
# s. inversions	0
# misassembled contigs	2
Misassembled contigs length	3331849
# local misassemblies	44
# scaffold gap ext. mis.	0
# scaffold gap loc. mis.	0
# unaligned mis. contigs	0
# mismatches	20721
# indels	622
# indels (<= 5 bp)	529
# indels (> 5 bp)	93
Indels length	6292

All statistics are based on contigs of size >= 500 bp, unless otherwise noted (e.g., "# contigs (>= 0 bp)" and "Total length (>= 0 bp)" include all contigs).

Unaligned report

	EH1.3_final
# fully unaligned contigs	1
Fully unaligned length	19309
# partially unaligned contigs	2
Partially unaligned length	729068
# N's	0

All statistics are based on contigs of size >= 500 bp, unless otherwise noted (e.g., "# contigs (>= 0 bp)" and "Total length (>= 0 bp)" include all contigs).

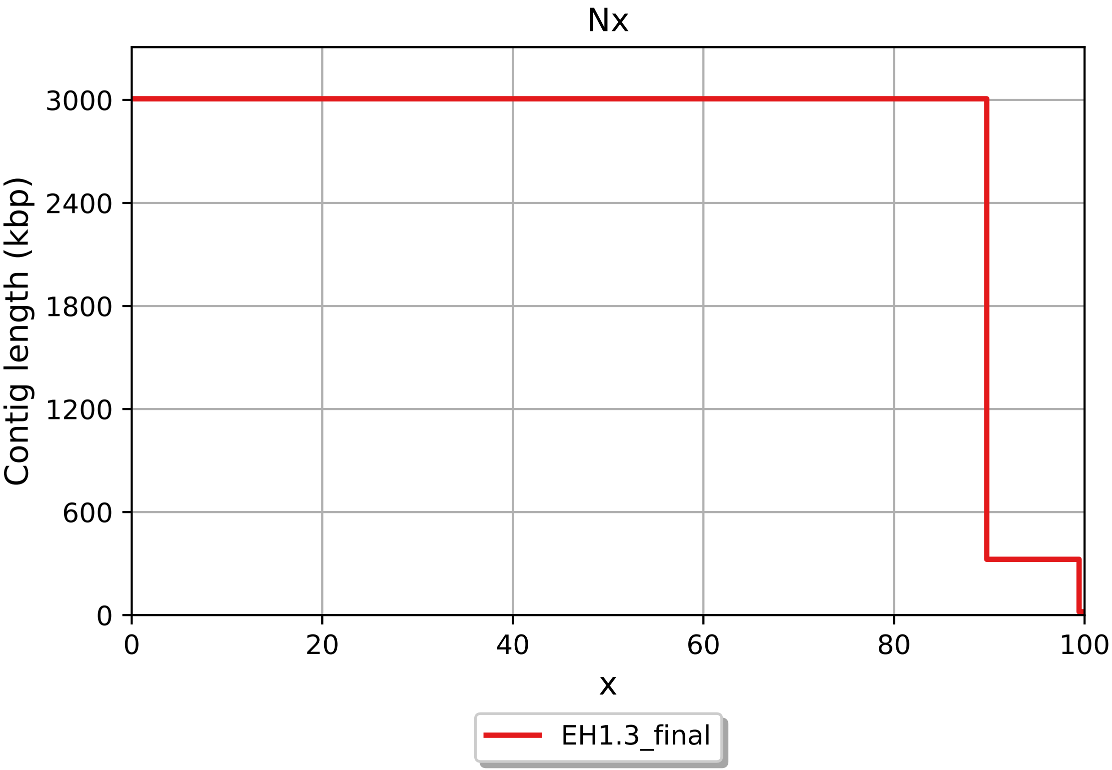
Nx
3000
2400
1800
1200
600
0
Contig length (kbp)
0
20
40
60
80
100
x
EH1.3_final

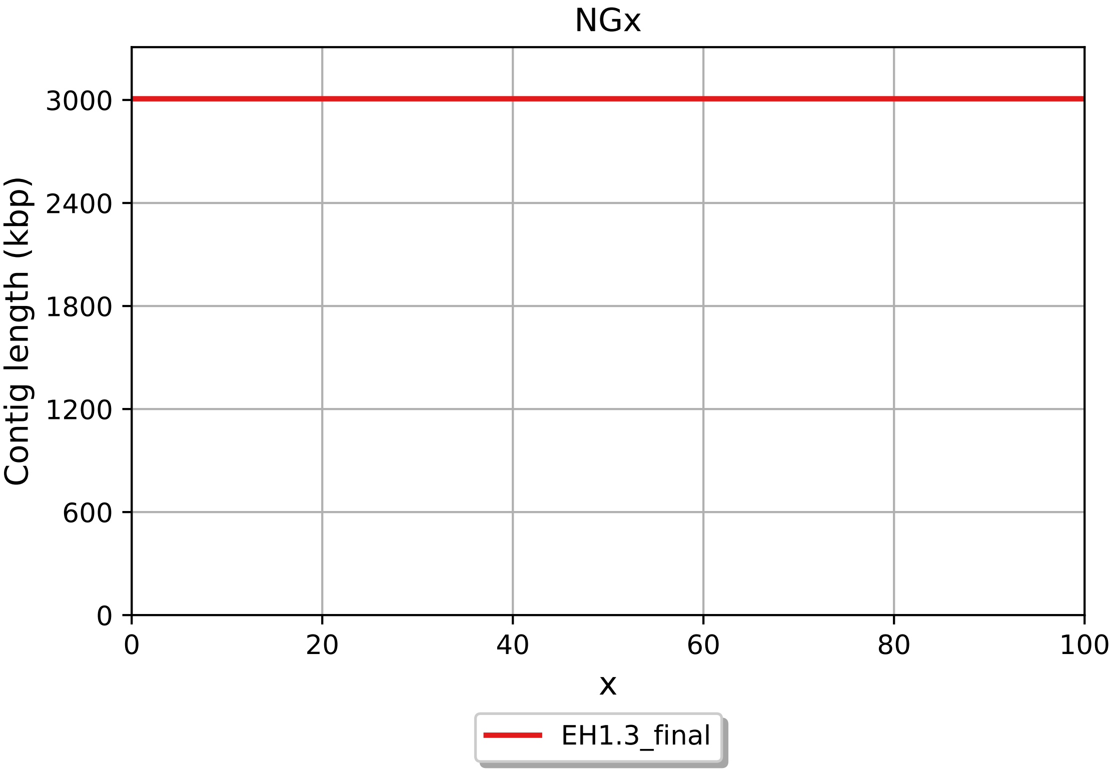

NGx
3000
2400
1800
1200
600
0
Contig length (kbp)
0
20
40
60
80
100
x
EH1.3_final

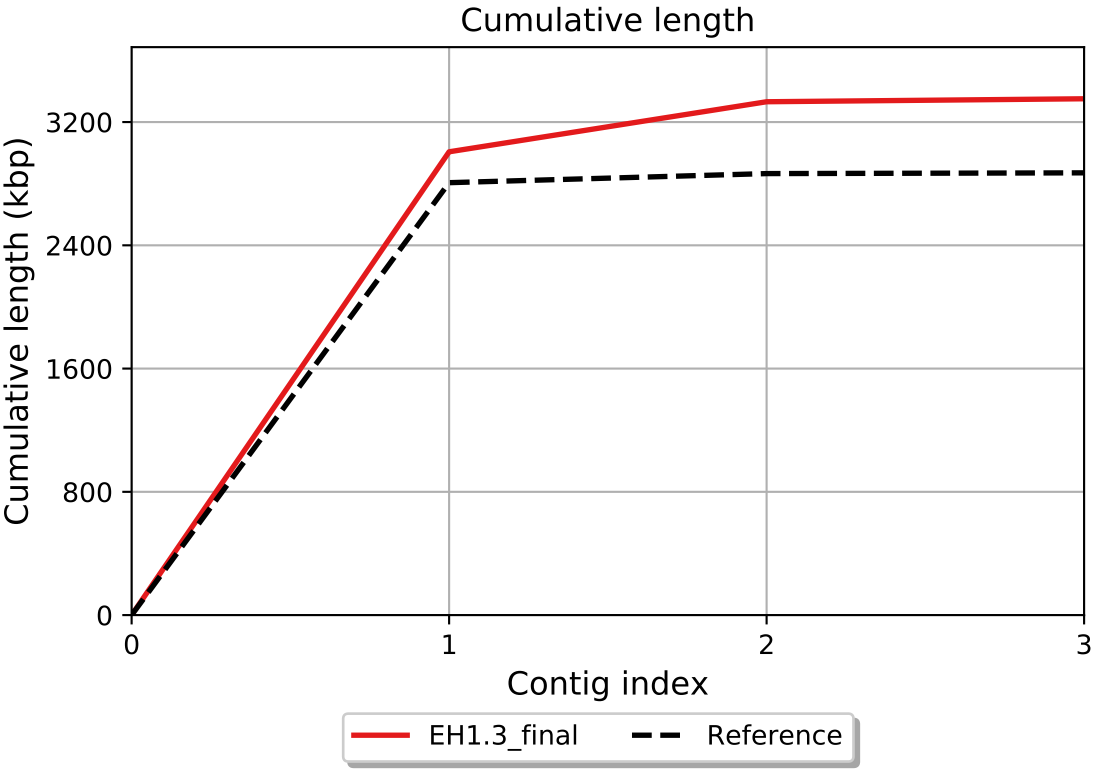
Cumulative length
Cumulative length (kbp)
0
800
1600
2400
3200
0
1
2
3
Contig index
EH1.3_final
Reference

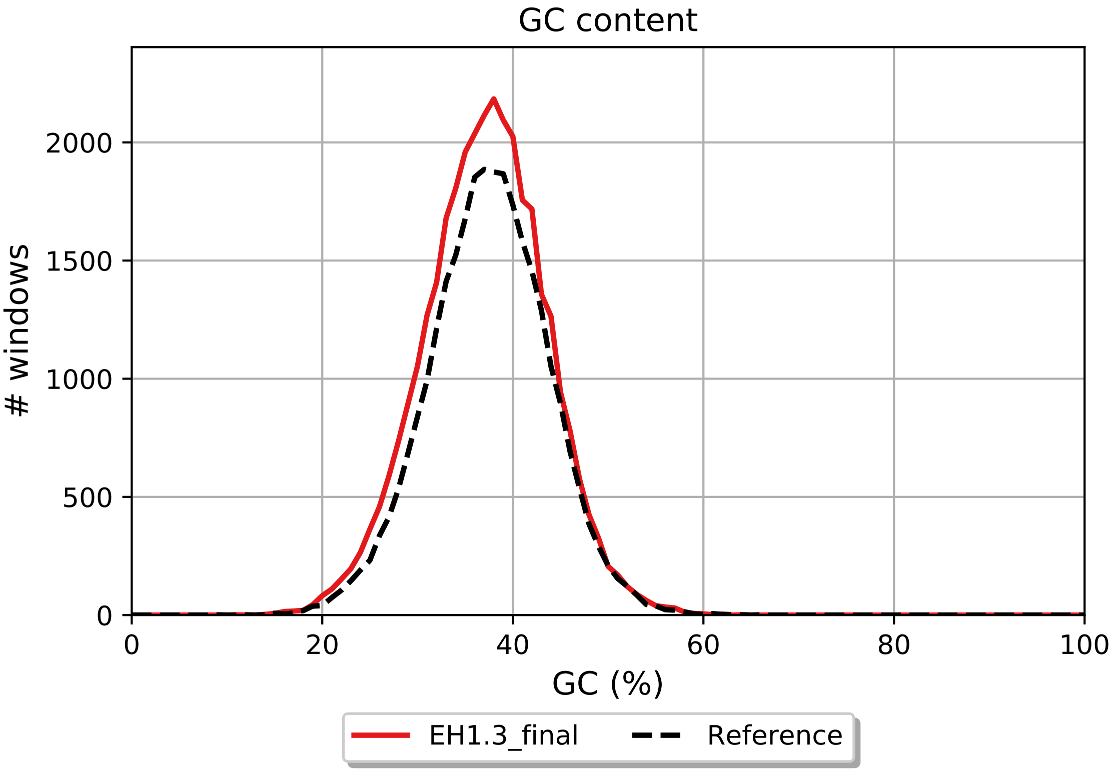
GC content
windows
2000
1500
1000
500
0
0
20
40
60
80
100
GC (%)
EH1.3_final
Reference

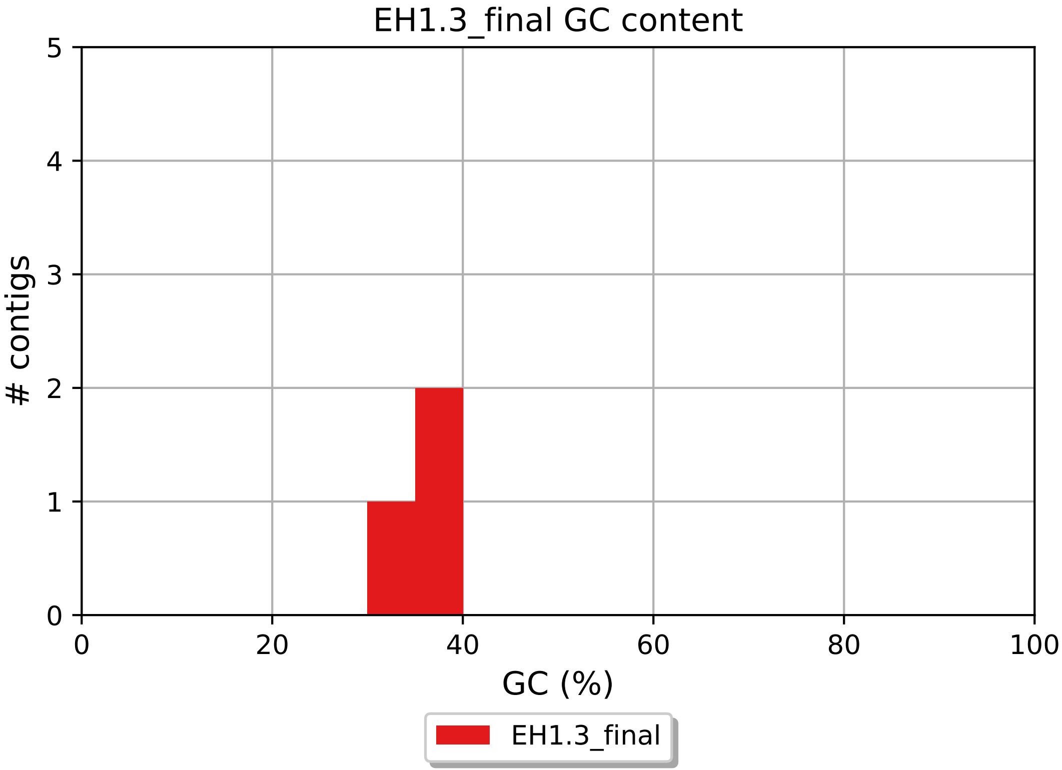
EH1.3_final GC content
contigs
0
1
2
3
4
5
0
20
40
60
80
100
GC (%)
EH1.3_final

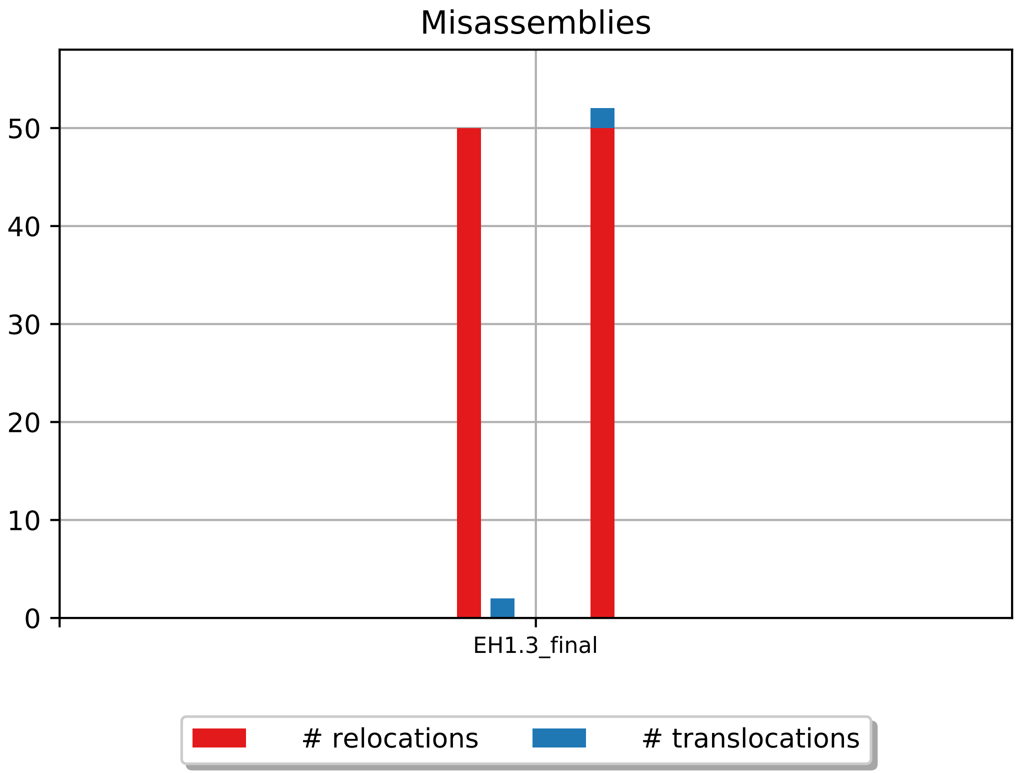
Misassemblies
50
40
30
20
10
0
EH1.3_final
relocations
translocations

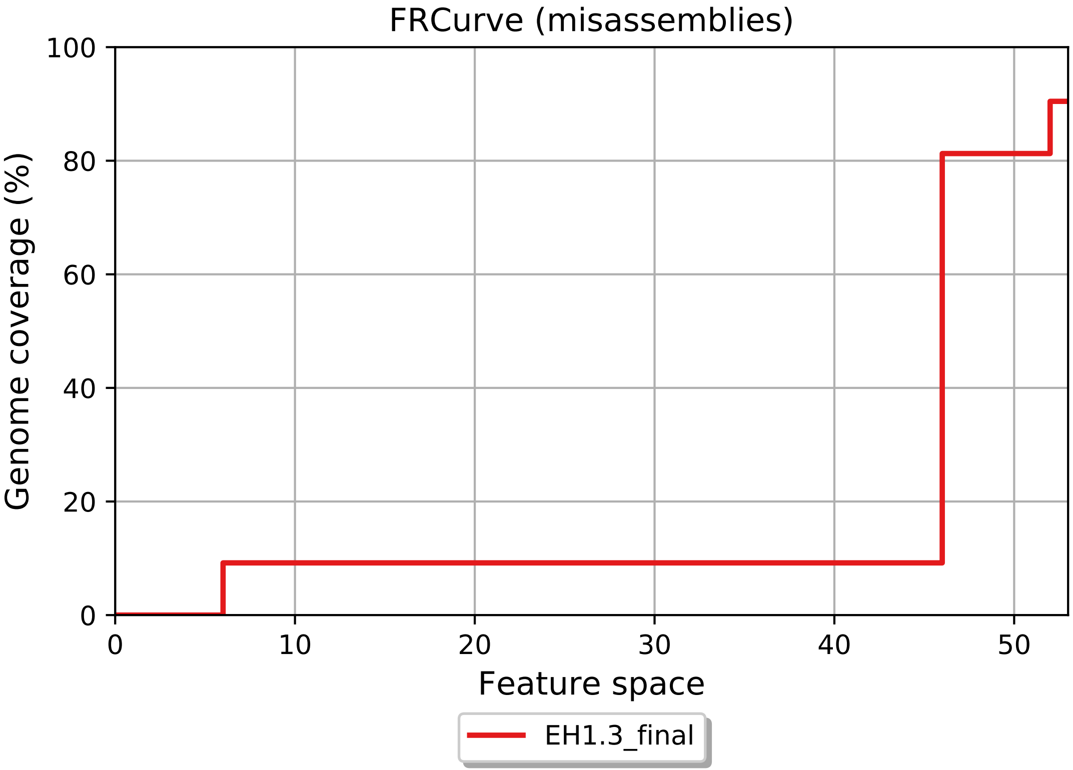
FRCurve (misassemblies)
Genome coverage (%)
100
80
60
40
20
0
0
10
20
30
40
50
Feature space
EH1.3_final

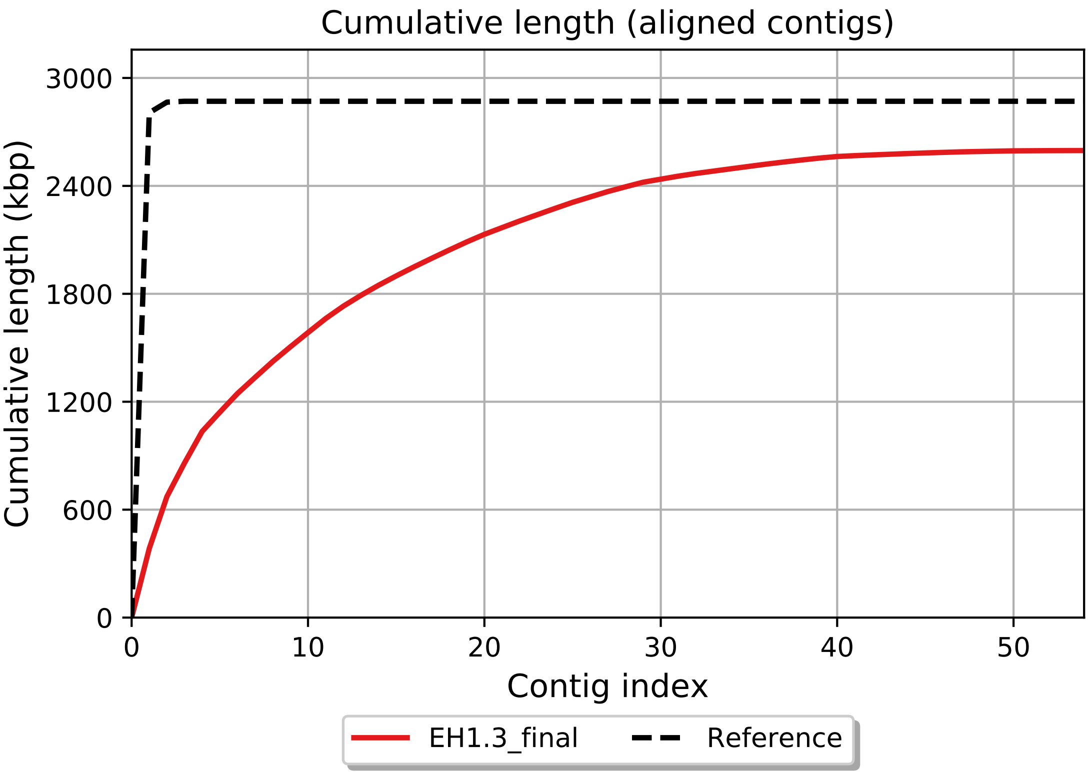

Cumulative length (aligned contigs)
Cumulative length (kbp)
0
600
1200
1800
2400
3000
0
10
20
30
40
50
Contig index
EH1.3_final
Reference

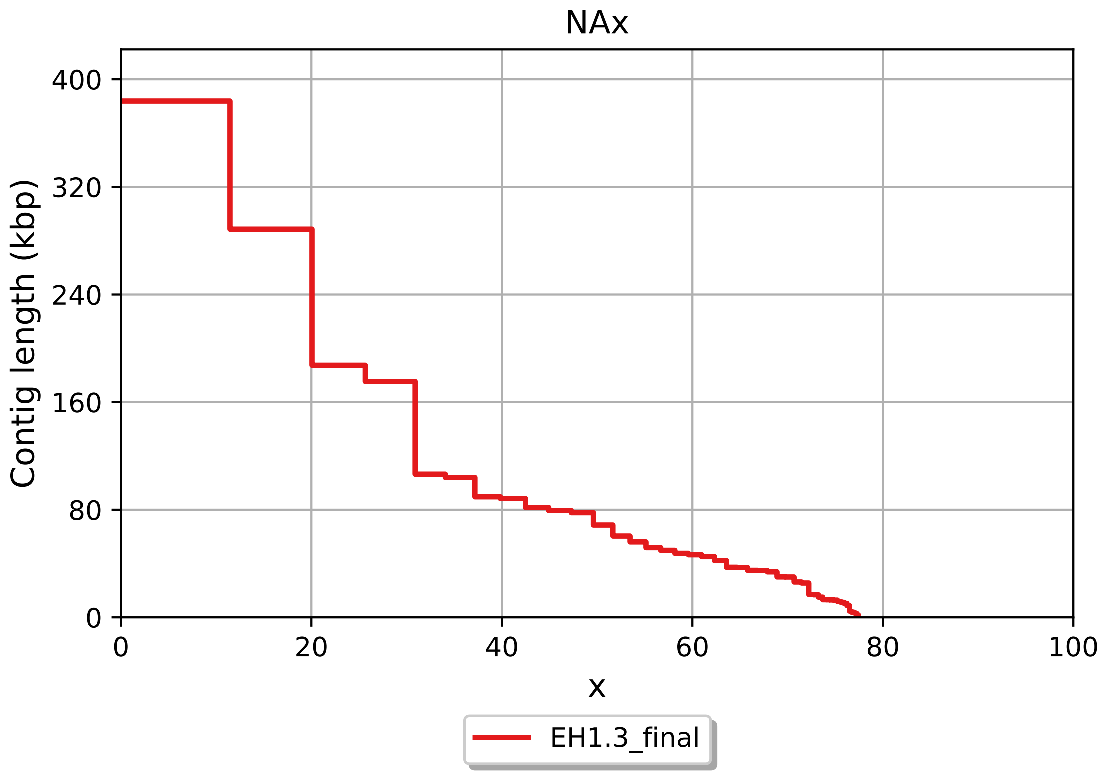
NAx
Contig length (kbp)
400
320
240
160
80
0
0
20
40
60
80
100
x
EH1.3_final

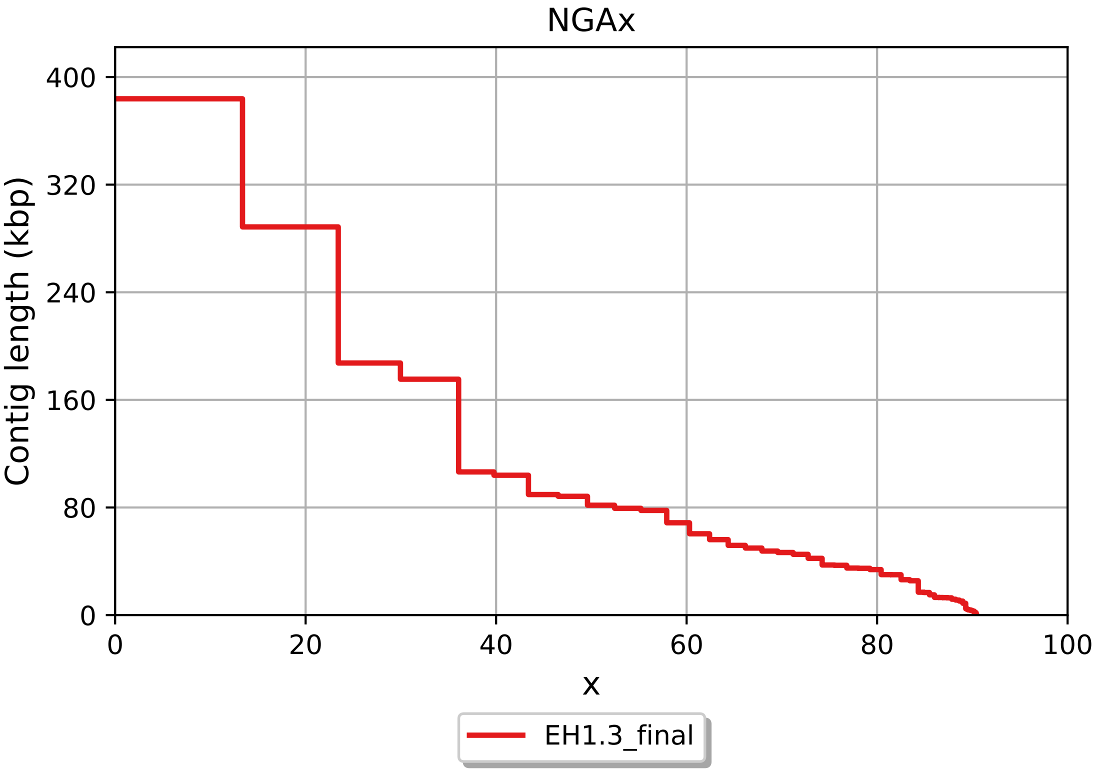

NGAx
Contig length (kbp)
400
320
240
160
80
0
0
20
40
60
80
100
x
EH1.3_final
