# Supplementary material for: Candidate Genomic Features Associated with Persistence in Enterococcus spp
Source: Microorganisms. 2026 Apr 19;14(4):921. doi: 10.3390/microorganisms14040921 (PMC13119490; doi:10.3390/microorganisms14040921)
Supplement: Supplementary file 1 [file microorganisms-14-00921-s001.zip › Supplementary File S1/QUAST/EH1_negative/report.pdf]

Report

	EH1_negative_final
# contigs (>= 0 bp)	3
# contigs (>= 1000 bp)	3
# contigs (>= 5000 bp)	3
# contigs (>= 10000 bp)	3
# contigs (>= 25000 bp)	1
# contigs (>= 50000 bp)	1
Total length (>= 0 bp)	3341712
Total length (>= 1000 bp)	3341712
Total length (>= 5000 bp)	3341712
Total length (>= 10000 bp)	3341712
Total length (>= 25000 bp)	3309807
Total length (>= 50000 bp)	3309807
# contigs	3
Largest contig	3309807
Total length	3341712
Reference length	2870381
GC (%)	37.09
Reference GC (%)	37.47
N50	3309807
NG50	3309807
N90	3309807
NG90	3309807
auN	3278365.7
auNG	3816689.8
L50	1
LG50	1
L90	1
LG90	1
# misassemblies	54
# misassembled contigs	1
Misassembled contigs length	3309807
# local misassemblies	43
# scaffold gap ext. mis.	0
# scaffold gap loc. mis.	0
# unaligned mis. contigs	0
# unaligned contigs	2 + 1 part
Unaligned length	738852
Genome fraction (%)	90.752
Duplication ratio	1.002
# N's per 100 kbp	0.00
# mismatches per 100 kbp	796.01
# indels per 100 kbp	24.01
Largest alignment	383837
Total aligned length	2602739
NA50	60448
NGA50	79380
NA90	-
NGA90	3781
auNA	116299.7
auNGA	135396.7
LA50	12
LGA50	9
LA90	-
LGA90	45

All statistics are based on contigs of size >= 500 bp, unless otherwise noted (e.g., "# contigs (>= 0 bp)" and "Total length (>= 0 bp)" include all contigs).

Misassemblies report

	EH1_negative_final
# misassemblies	54
# contig misassemblies	54
# c. relocations	52
# c. translocations	2
# c. inversions	0
# scaffold misassemblies	0
# s. relocations	0
# s. translocations	0
# s. inversions	0
# misassembled contigs	1
Misassembled contigs length	3309807
# local misassemblies	43
# scaffold gap ext. mis.	0
# scaffold gap loc. mis.	0
# unaligned mis. contigs	0
# mismatches	20718
# indels	625
# indels (<= 5 bp)	532
# indels (> 5 bp)	93
Indels length	6295

All statistics are based on contigs of size >= 500 bp, unless otherwise noted (e.g., "# contigs (>= 0 bp)" and "Total length (>= 0 bp)" include all contigs).

Unaligned report

	EH1_negative_final
# fully unaligned contigs	2
Fully unaligned length	31905
# partially unaligned contigs	1
Partially unaligned length	706947
# N's	0

All statistics are based on contigs of size >= 500 bp, unless otherwise noted (e.g., "# contigs (>= 0 bp)" and "Total length (>= 0 bp)" include all contigs).

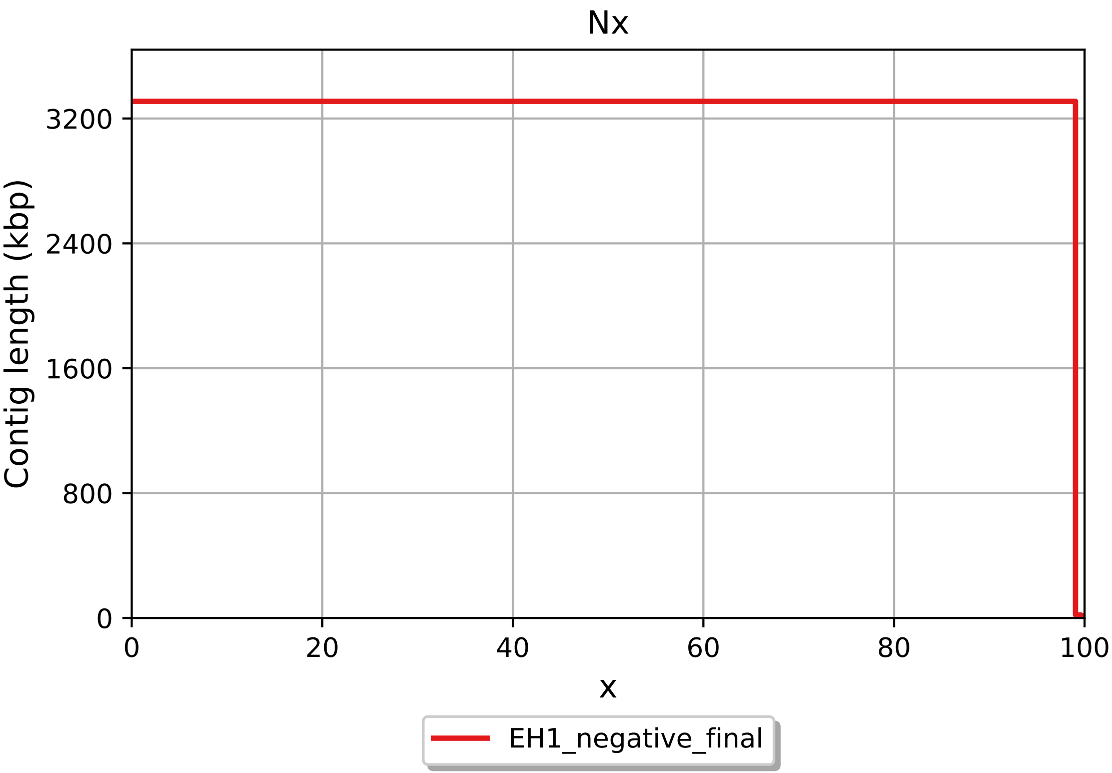
Nx
Contig length (kbp)
3200
2400
1600
800
0
0
20
40
60
80
100
x
EH1_negative_final

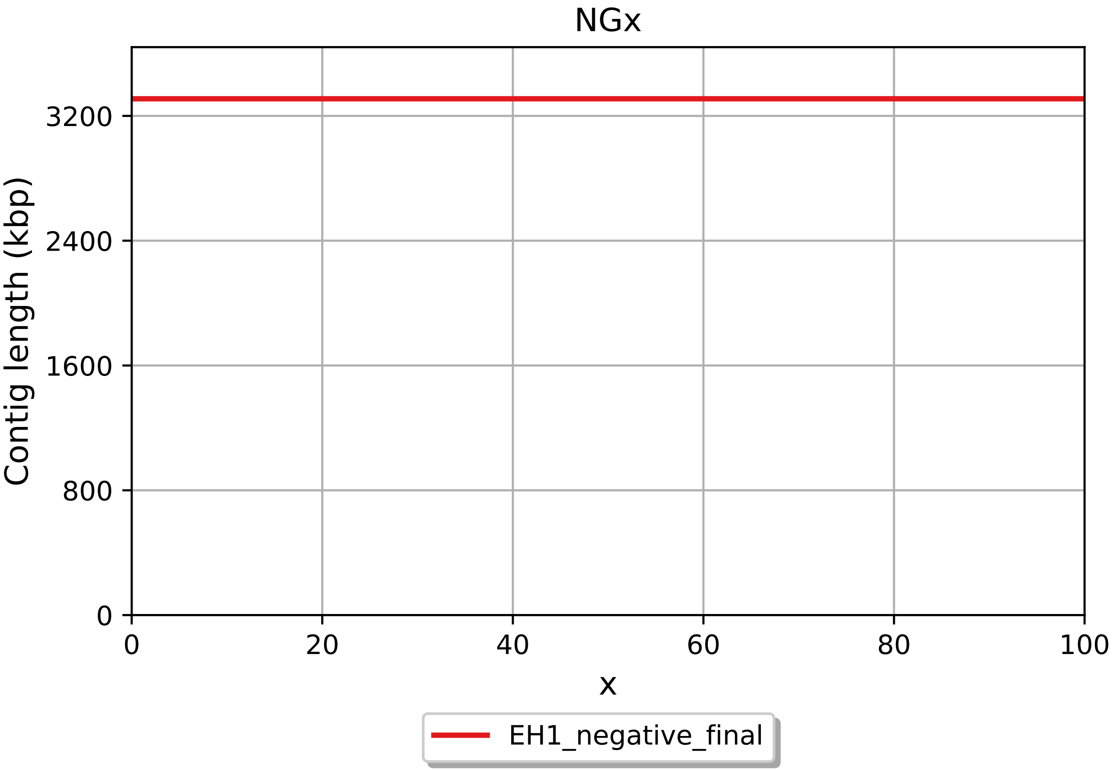
NGx
Contig length (kbp)
3200
2400
1600
800
0
0
20
40
60
80
100
x
EH1_negative_final

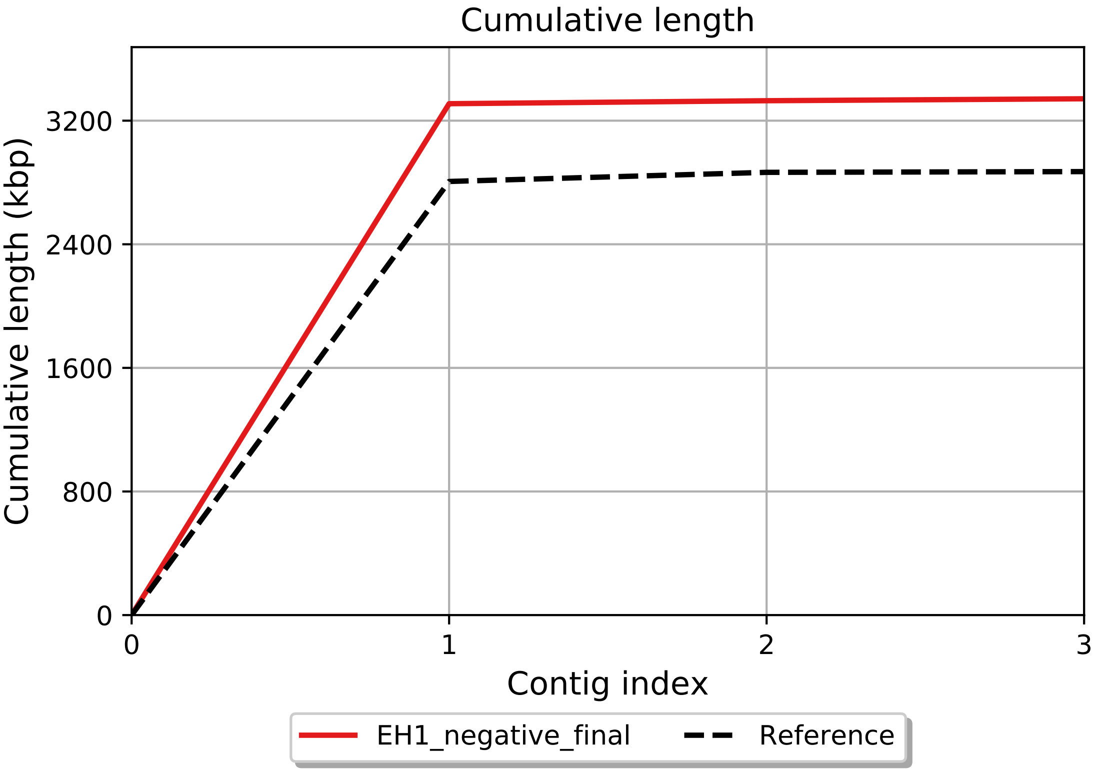
Cumulative length
Cumulative length (kbp)
0
800
1600
2400
3200
0
1
2
3
Contig index
EH1_negative_final
Reference

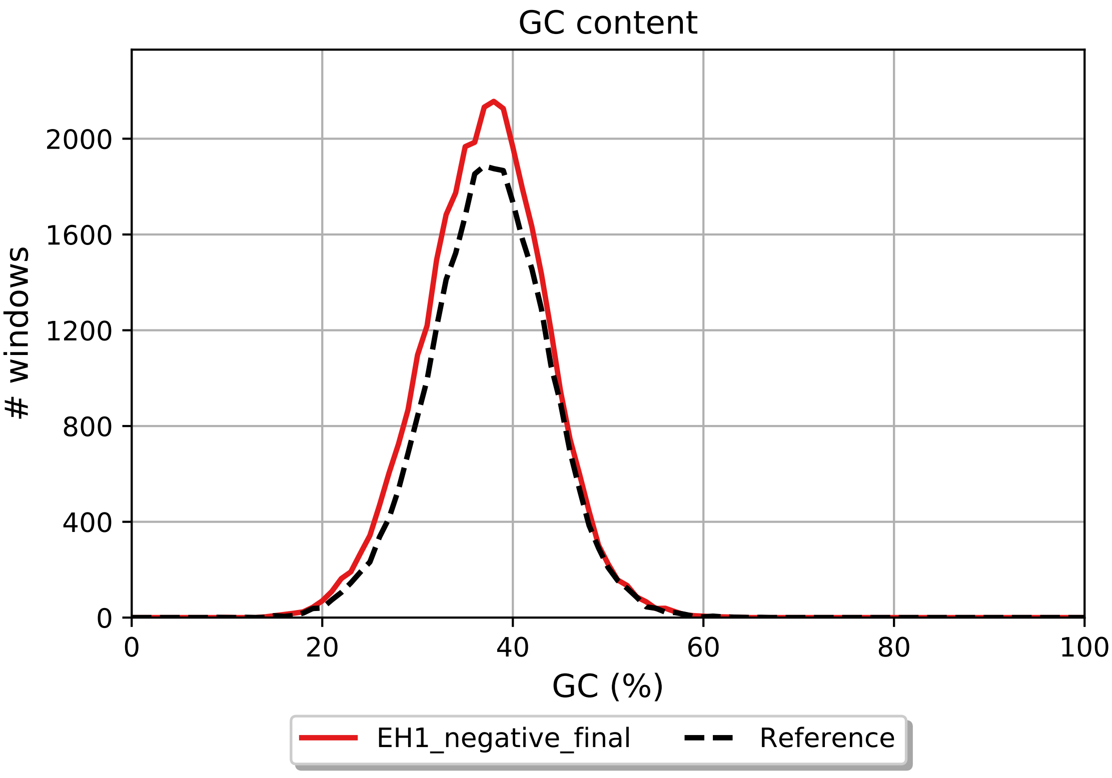
GC content
windows
2000
1600
1200
800
400
0
0
20
40
60
80
100
GC (%)
EH1_negative_final
Reference

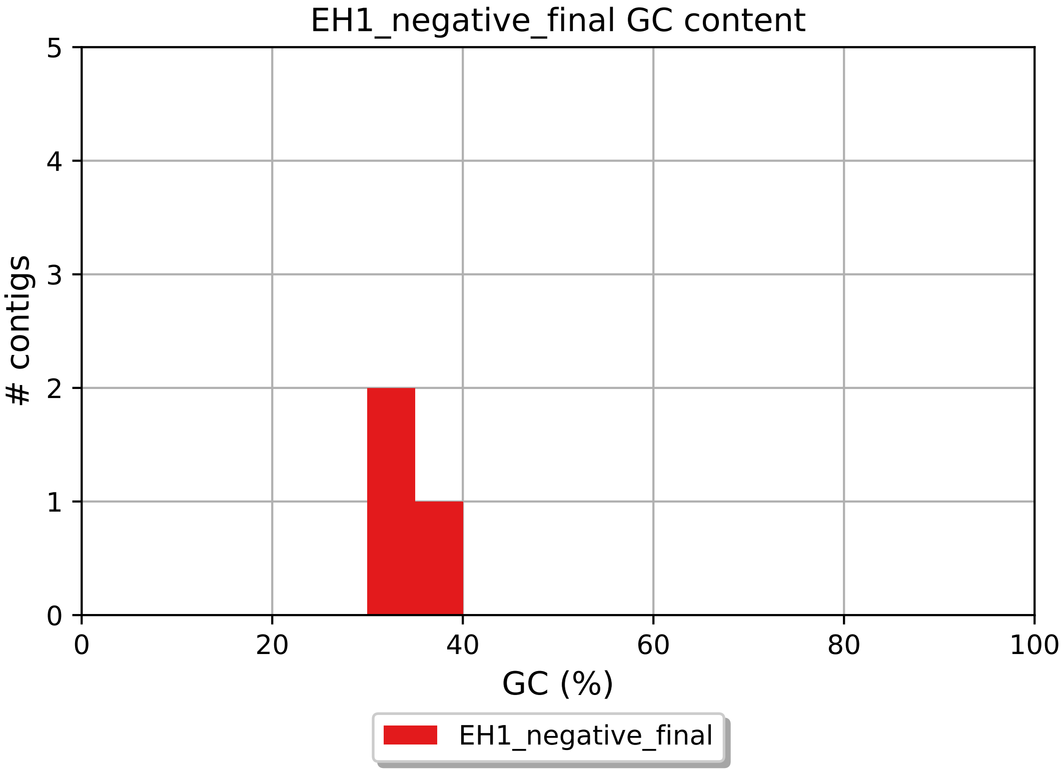
EH1_negative_final GC content
contigs
GC (%)
0
1
2
3
4
5
0
20
40
60
80
100
EH1_negative_final

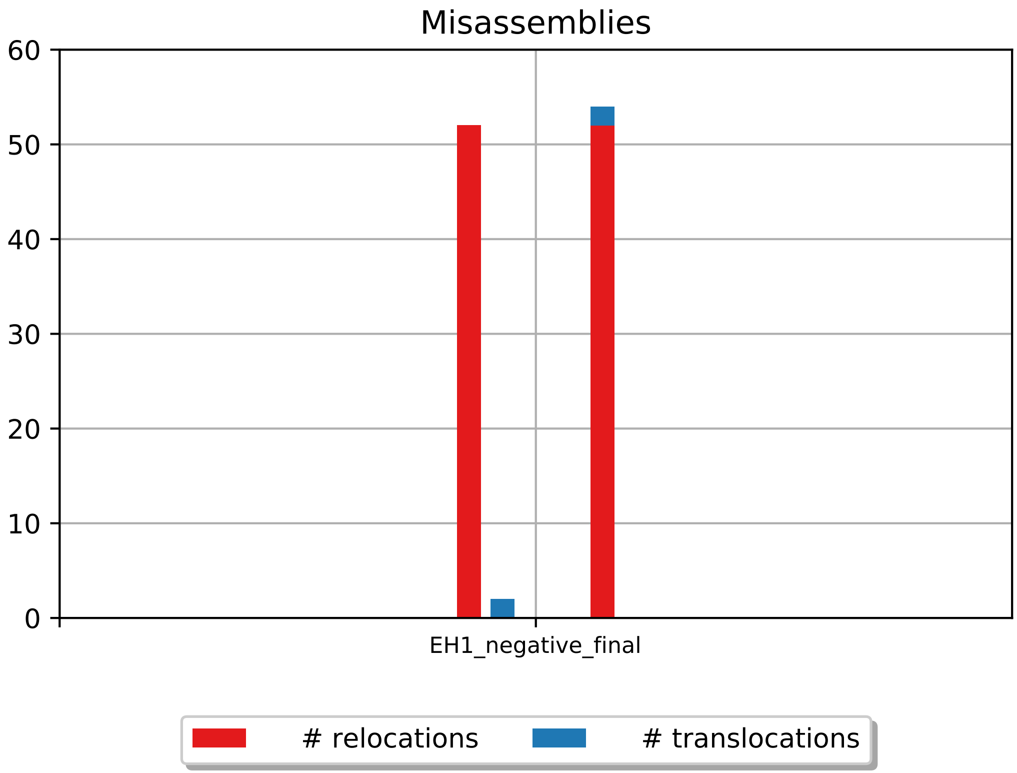

Misassemblies
60
50
40
30
20
10
0
EH1_negative_final
relocations
translocations

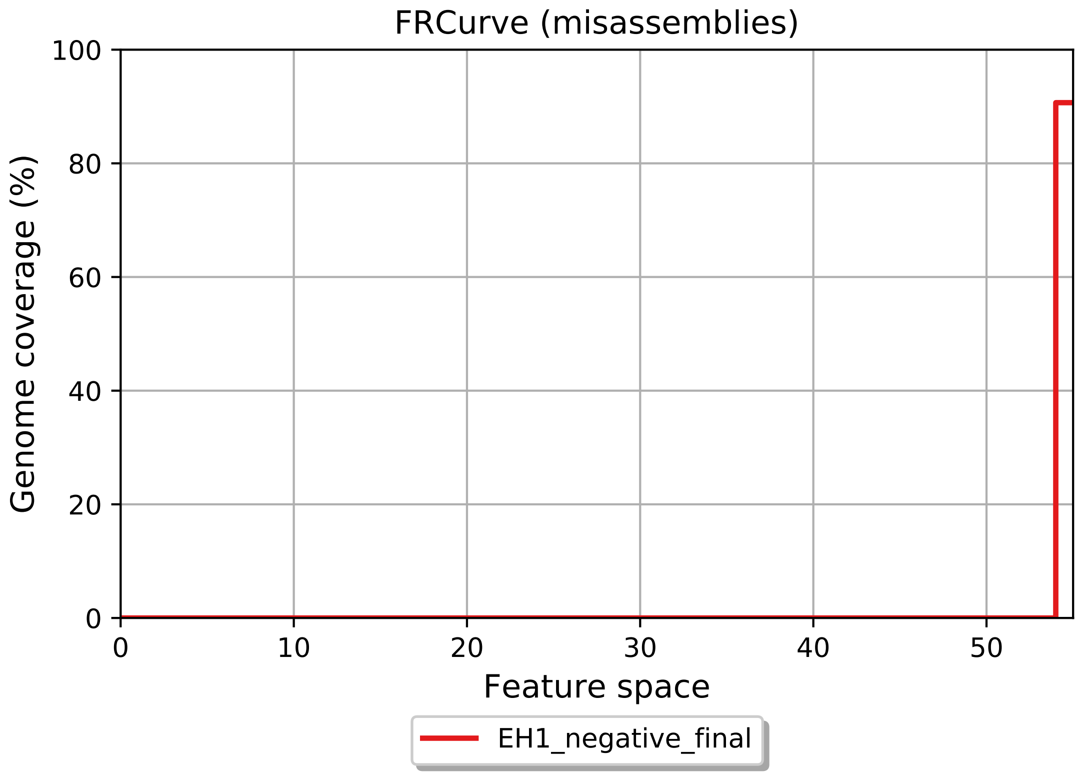
FRCurve (misassemblies)
Genome coverage (%)
100
80
60
40
20
0
0
10
20
30
40
50
Feature space
EH1_negative_final

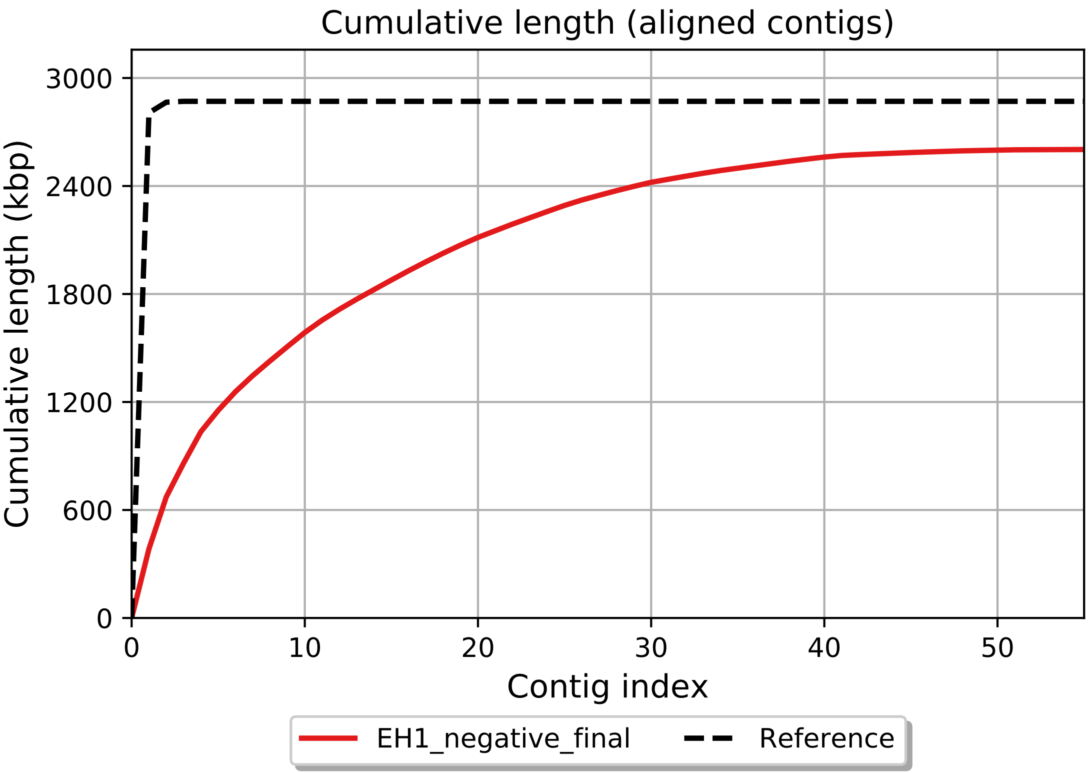

Cumulative length (aligned contigs)
Cumulative length (kbp)
3000
2400
1800
1200
600
0
0
10
20
30
40
50
Contig index
EH1_negative_final
Reference

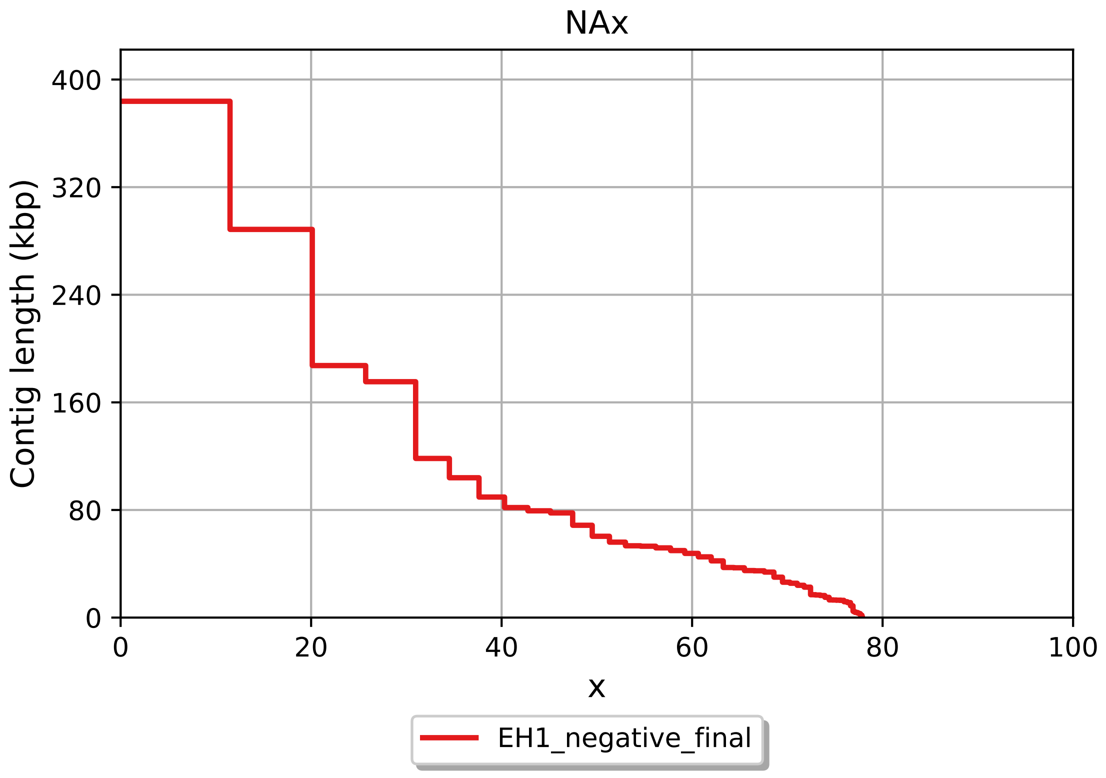
NAx
Contig length (kbp)
400
320
240
160
80
0
0
20
40
60
80
100
x
EH1_negative_final

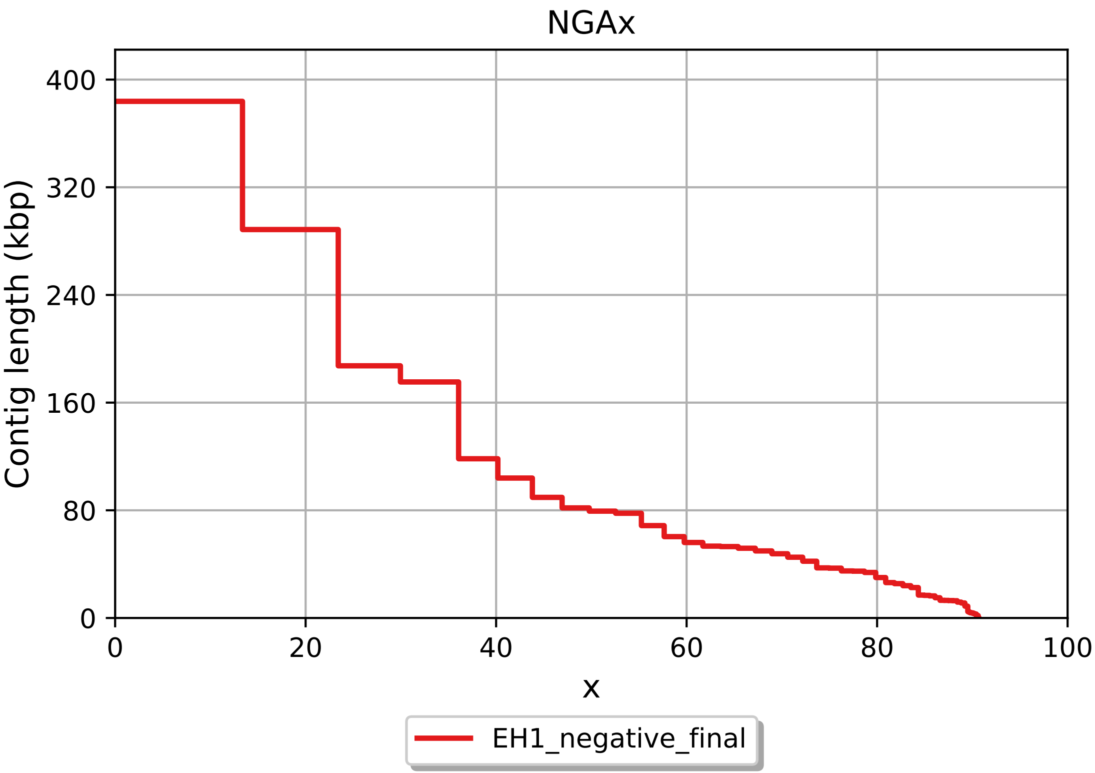

NGAx
Contig length (kbp)
400
320
240
160
80
0
0
20
40
60
80
100
x
EH1_negative_final
