# Supplementary figures and images for: Candidate Genomic Features Associated with Persistence in Enterococcus spp
Source: Microorganisms. 2026 Apr 19;14(4):921. doi: 10.3390/microorganisms14040921 (PMC13119490; doi:10.3390/microorganisms14040921)

Cumulative length (aligned contigs)

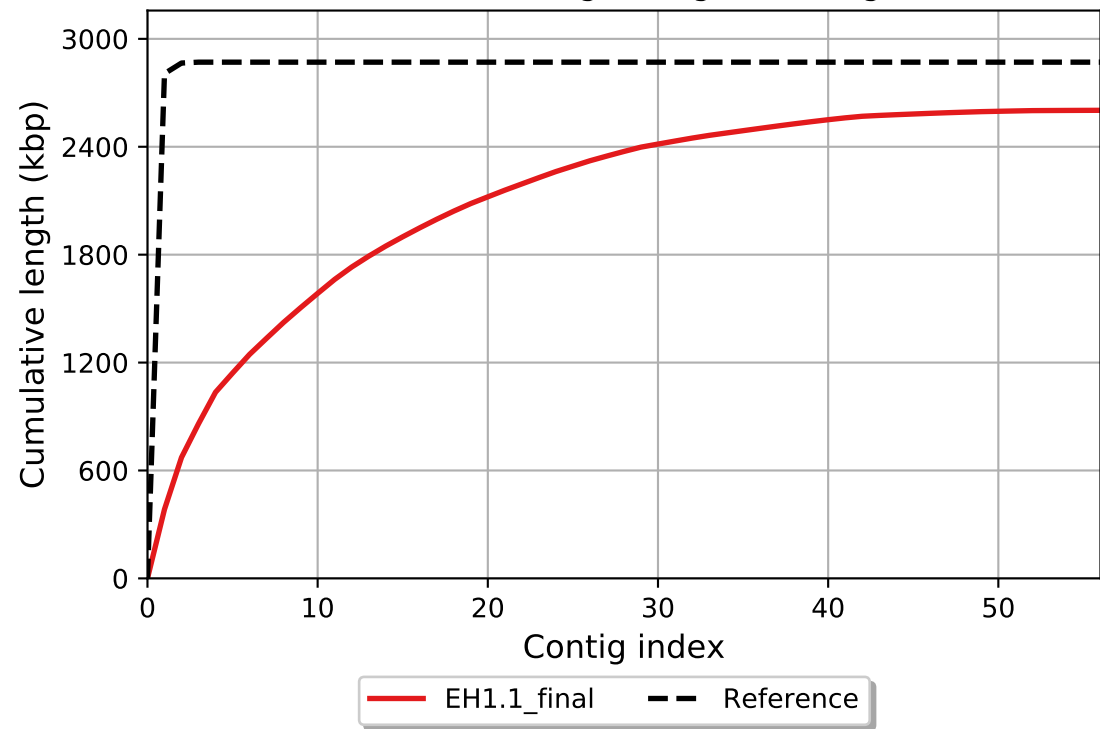

Supplement: Supplementary file 1 [file microorganisms-14-00921-s001.zip › Supplementary File S1/QUAST/EH1.1/aligned_stats/cumulative_plot.pdf]

NAx

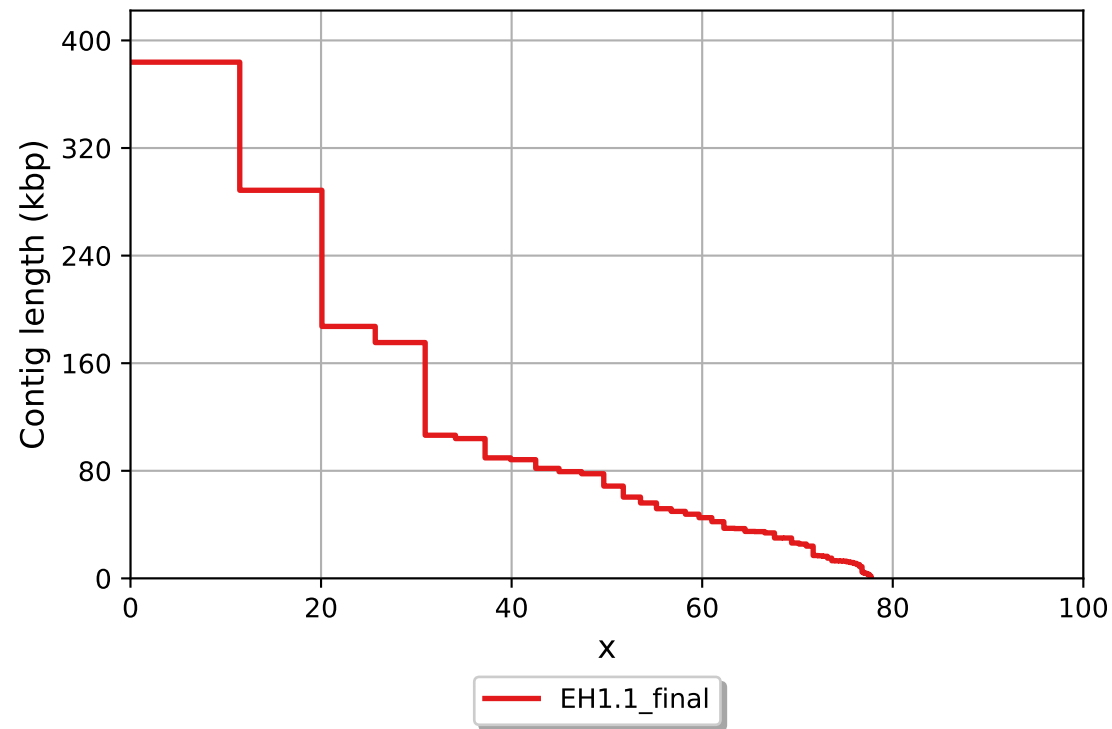

Supplement: Supplementary file 1 [file microorganisms-14-00921-s001.zip › Supplementary File S1/QUAST/EH1.1/aligned_stats/NAx_plot.pdf]

# NGAx

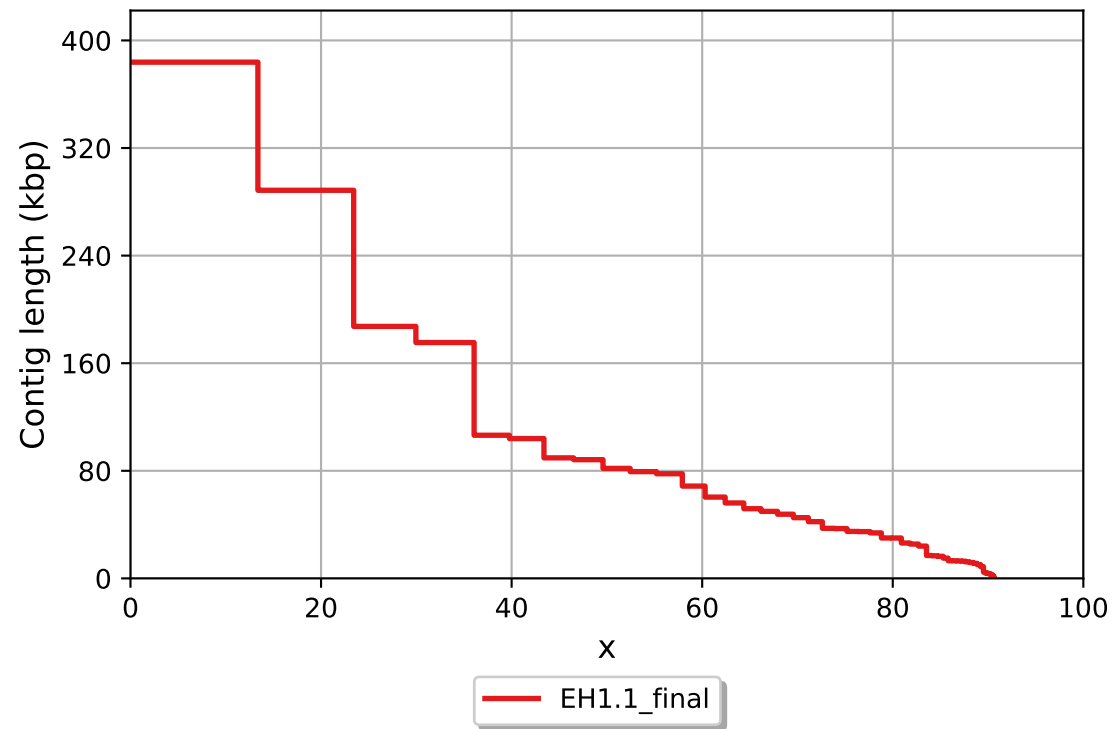

Supplement: Supplementary file 1 [file microorganisms-14-00921-s001.zip › Supplementary File S1/QUAST/EH1.1/aligned_stats/NGAx_plot.pdf]

Cumulative length

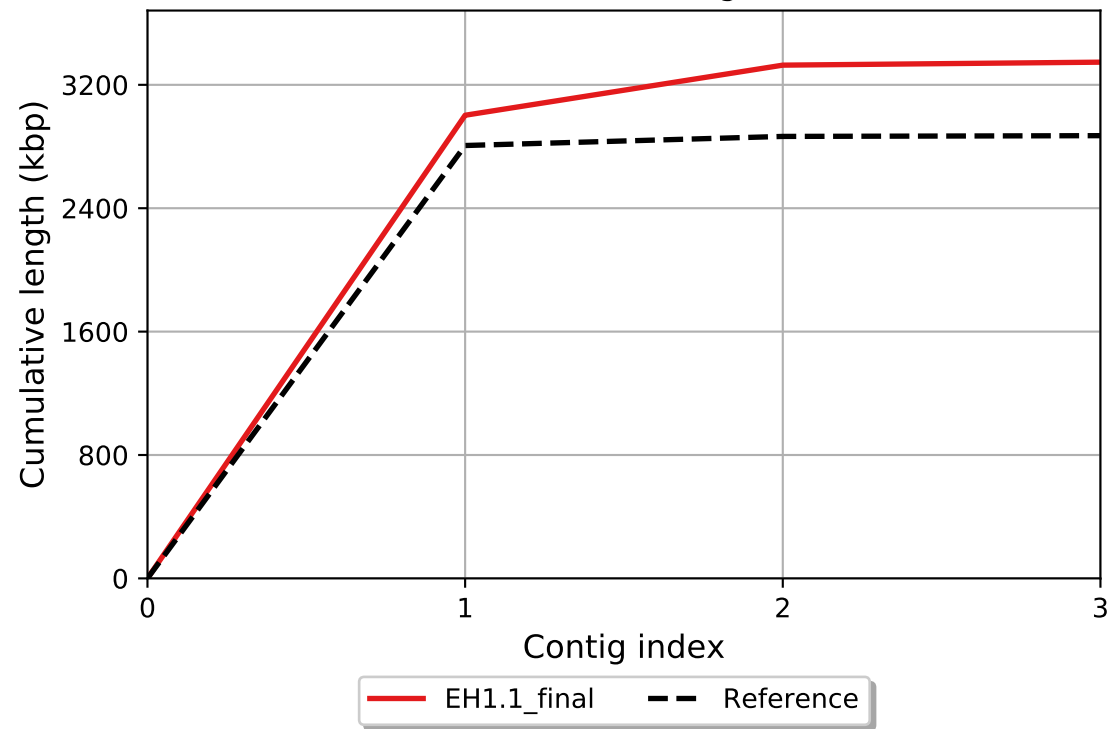

Supplement: Supplementary file 1 [file microorganisms-14-00921-s001.zip › Supplementary File S1/QUAST/EH1.1/basic_stats/cumulative_plot.pdf]

EH1.1\_final GC content

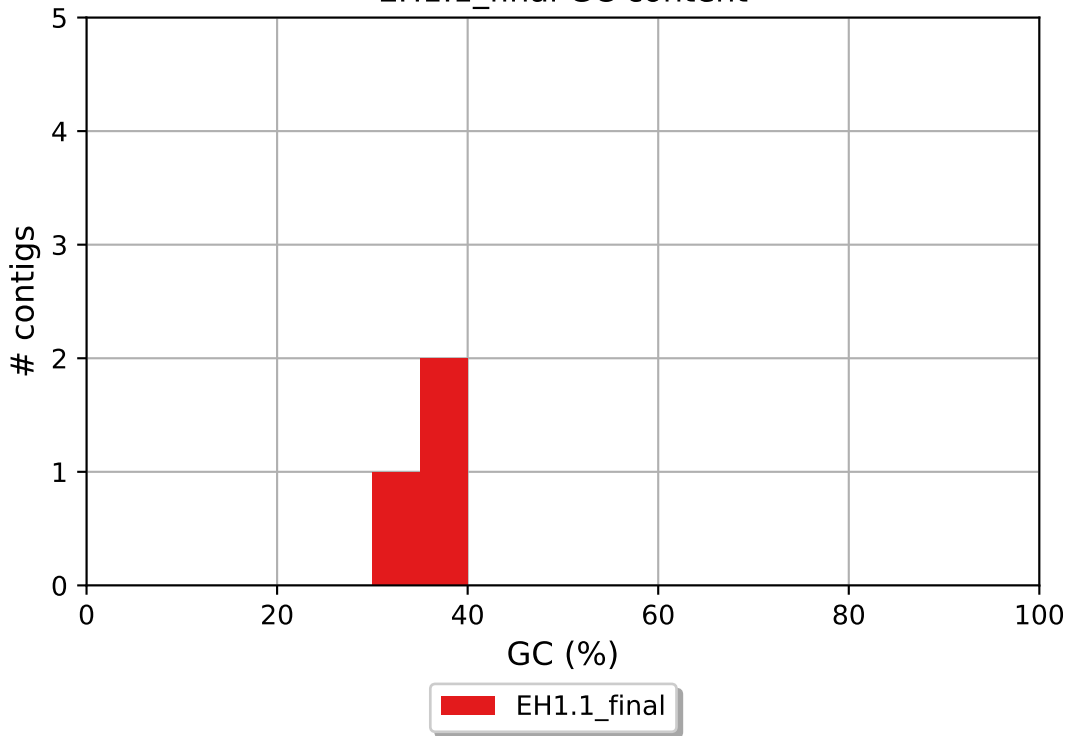

Supplement: Supplementary file 1 [file microorganisms-14-00921-s001.zip › Supplementary File S1/QUAST/EH1.1/basic_stats/EH1.1_final_GC_content_plot.pdf]

# GC content

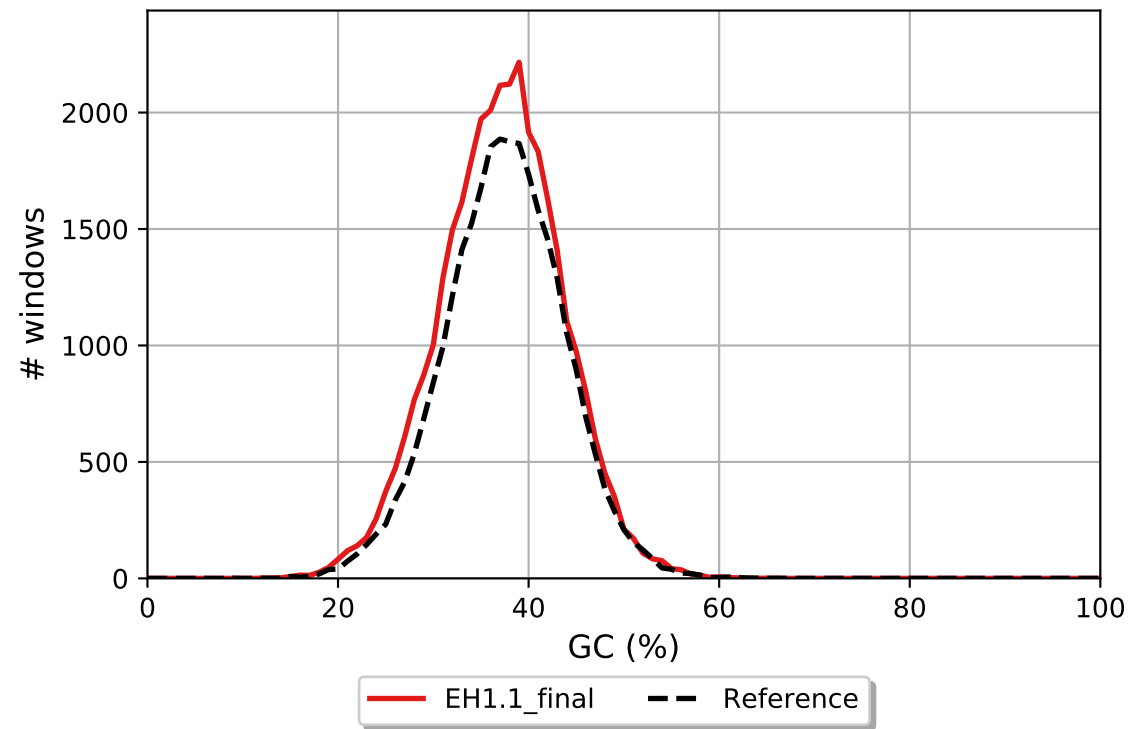

Supplement: Supplementary file 1 [file microorganisms-14-00921-s001.zip › Supplementary File S1/QUAST/EH1.1/basic_stats/GC_content_plot.pdf]

NGx

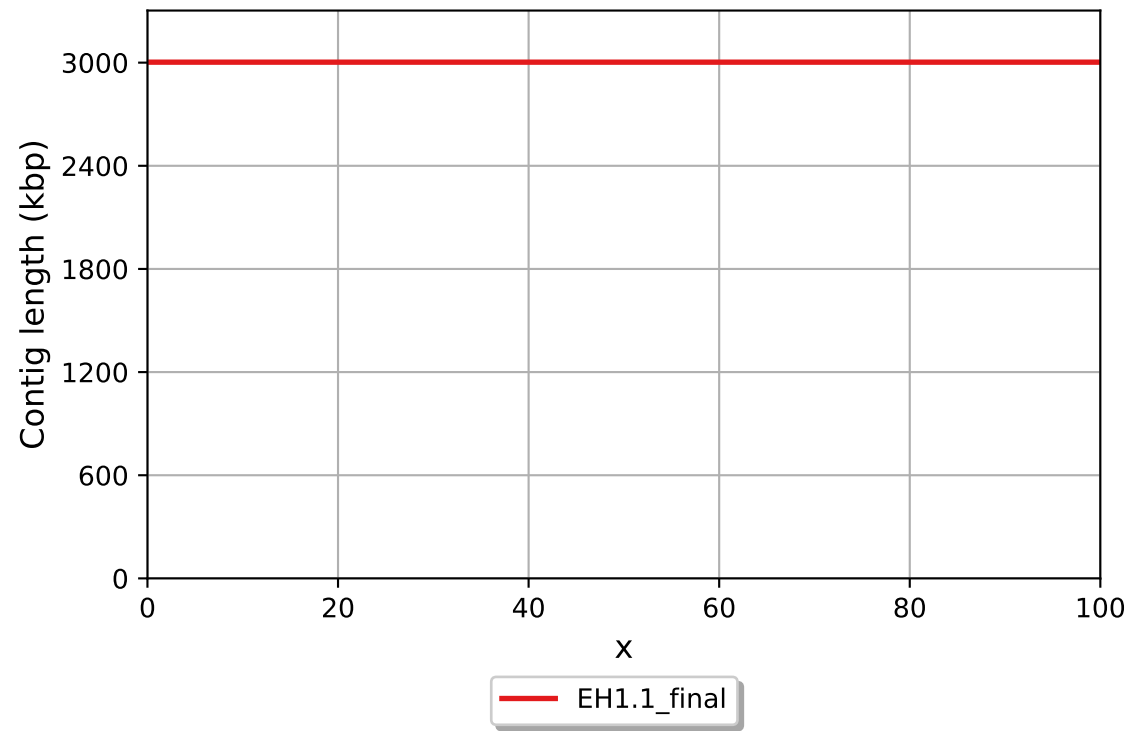

Supplement: Supplementary file 1 [file microorganisms-14-00921-s001.zip › Supplementary File S1/QUAST/EH1.1/basic_stats/NGx_plot.pdf]

Nx

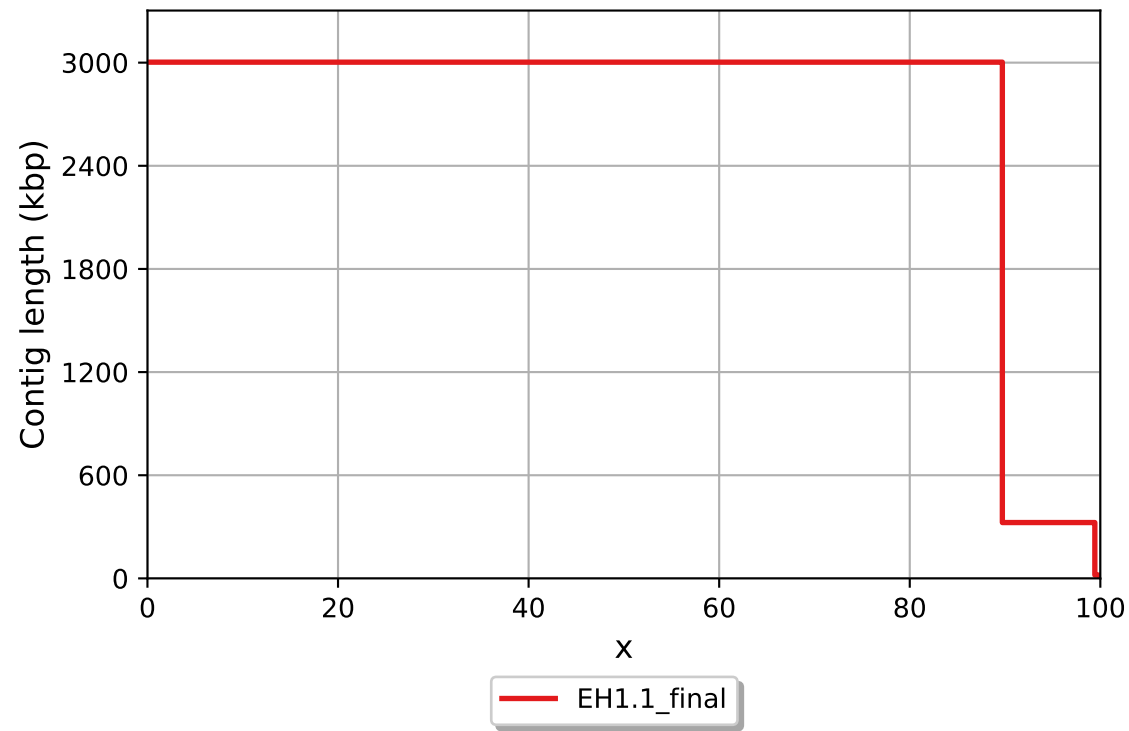

Supplement: Supplementary file 1 [file microorganisms-14-00921-s001.zip › Supplementary File S1/QUAST/EH1.1/basic_stats/Nx_plot.pdf]

FRCurve (misassemblies)

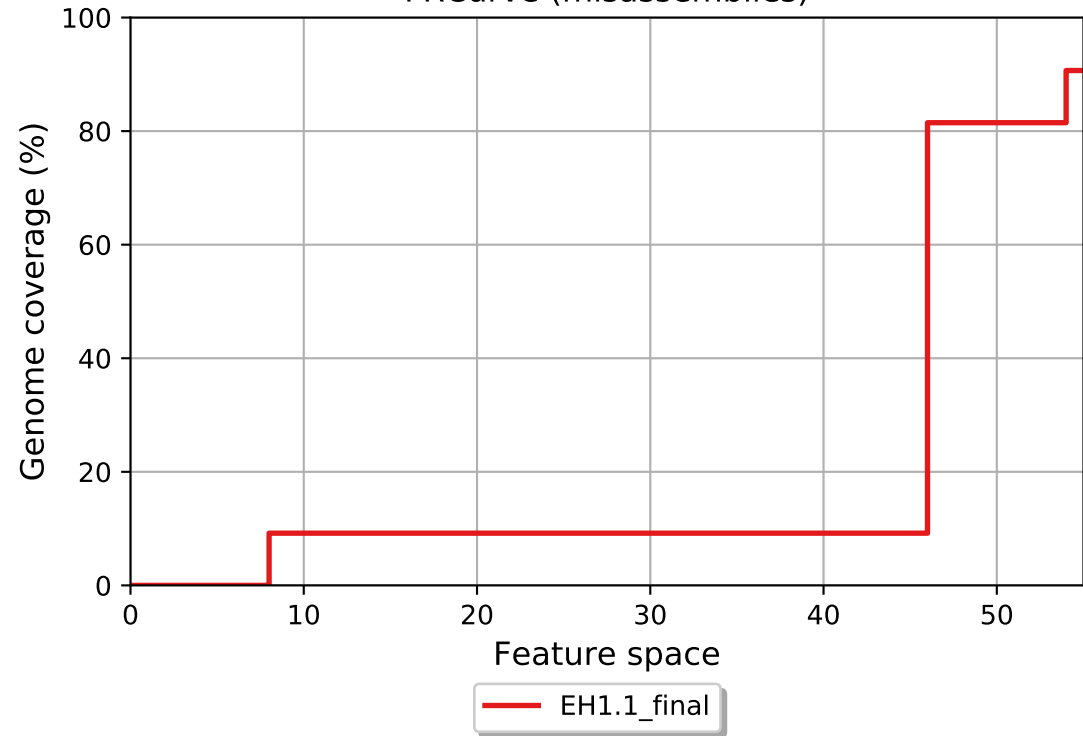

Supplement: Supplementary file 1 [file microorganisms-14-00921-s001.zip › Supplementary File S1/QUAST/EH1.1/contigs_reports/misassemblies_frcurve_plot.pdf]

# Misassemblies

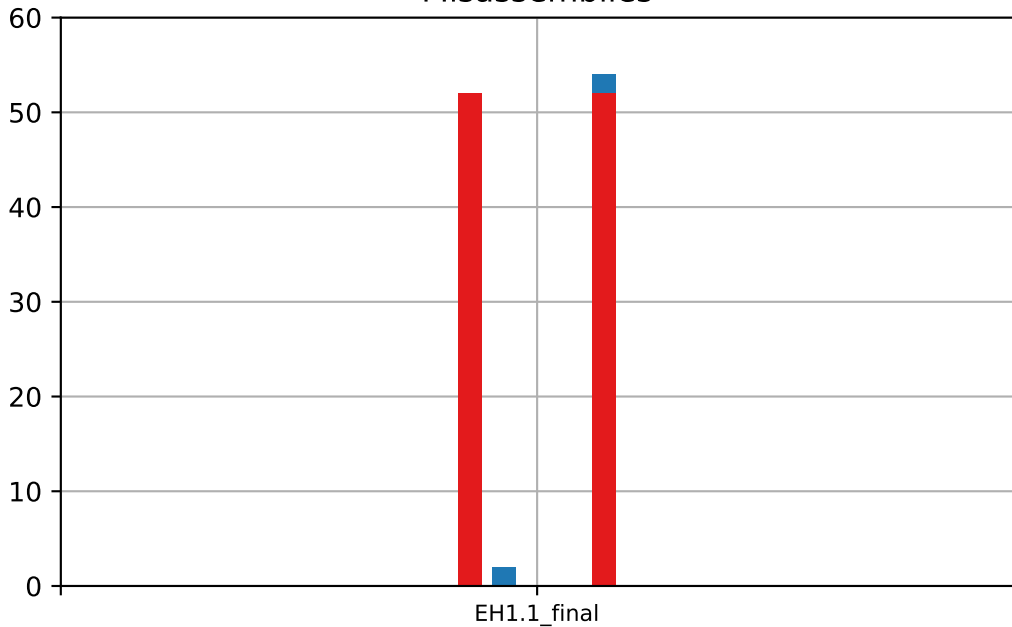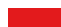

# relocations

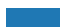

# translocations

Supplement: Supplementary file 1 [file microorganisms-14-00921-s001.zip › Supplementary File S1/QUAST/EH1.1/contigs_reports/misassemblies_plot.pdf]

Cumulative length (aligned contigs)

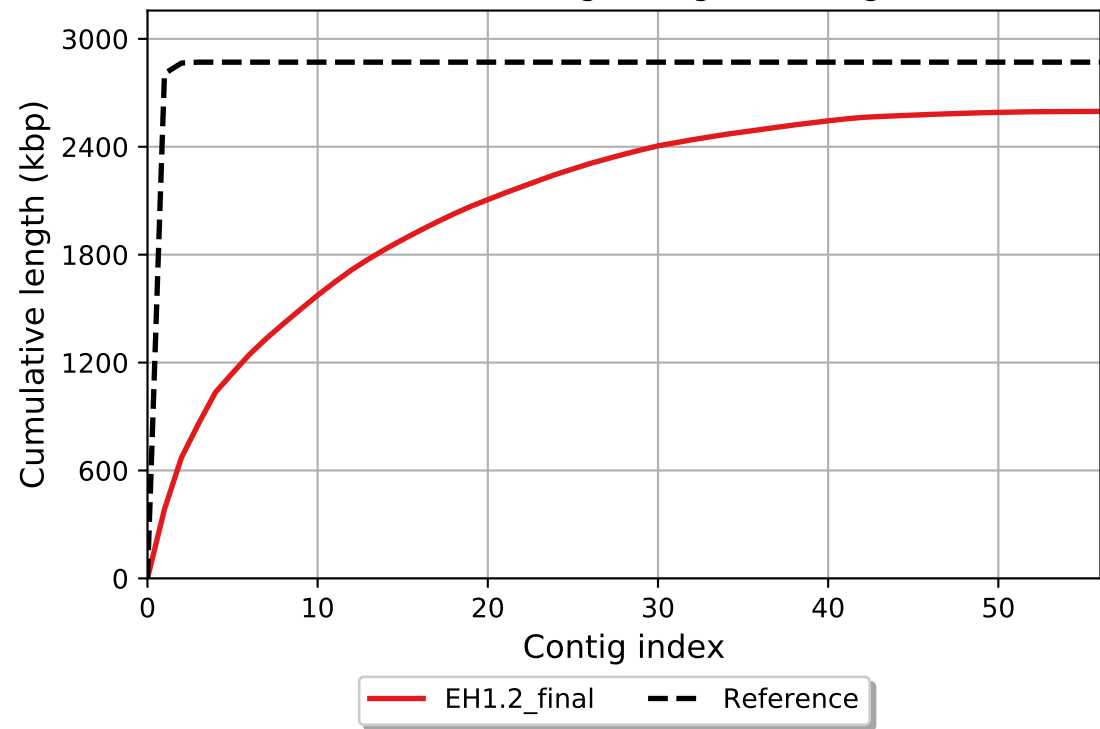

Supplement: Supplementary file 1 [file microorganisms-14-00921-s001.zip › Supplementary File S1/QUAST/EH1.2/aligned_stats/cumulative_plot.pdf]

NAx

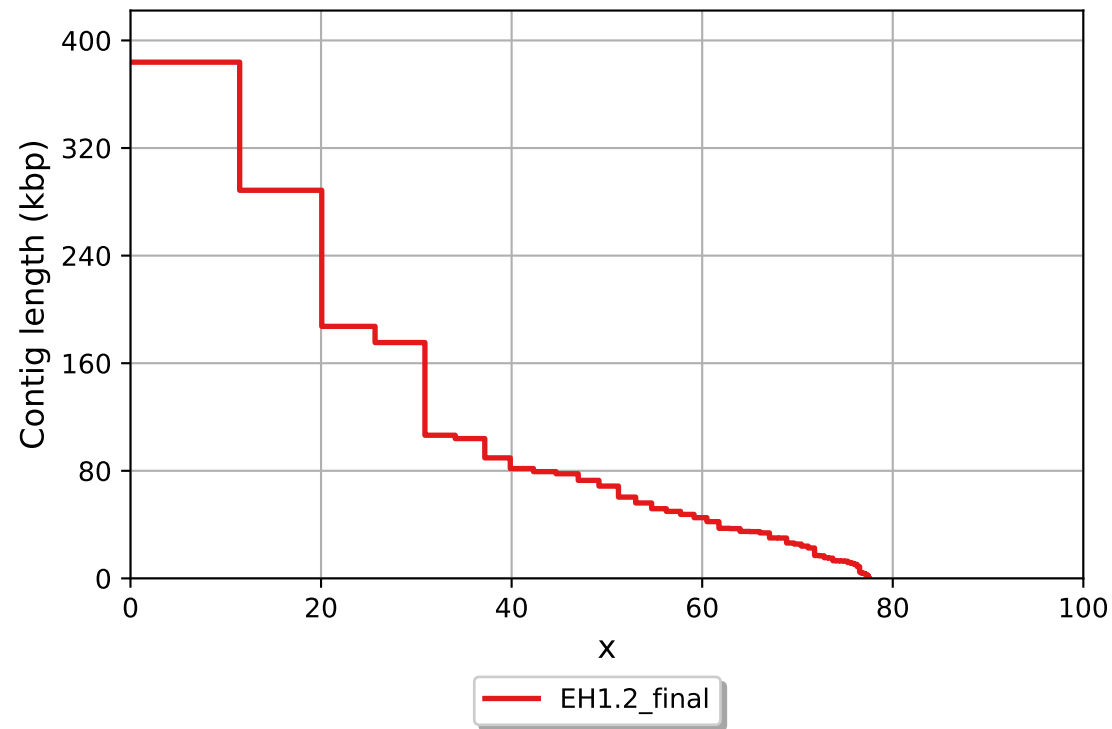

Supplement: Supplementary file 1 [file microorganisms-14-00921-s001.zip › Supplementary File S1/QUAST/EH1.2/aligned_stats/NAx_plot.pdf]

# NGAx

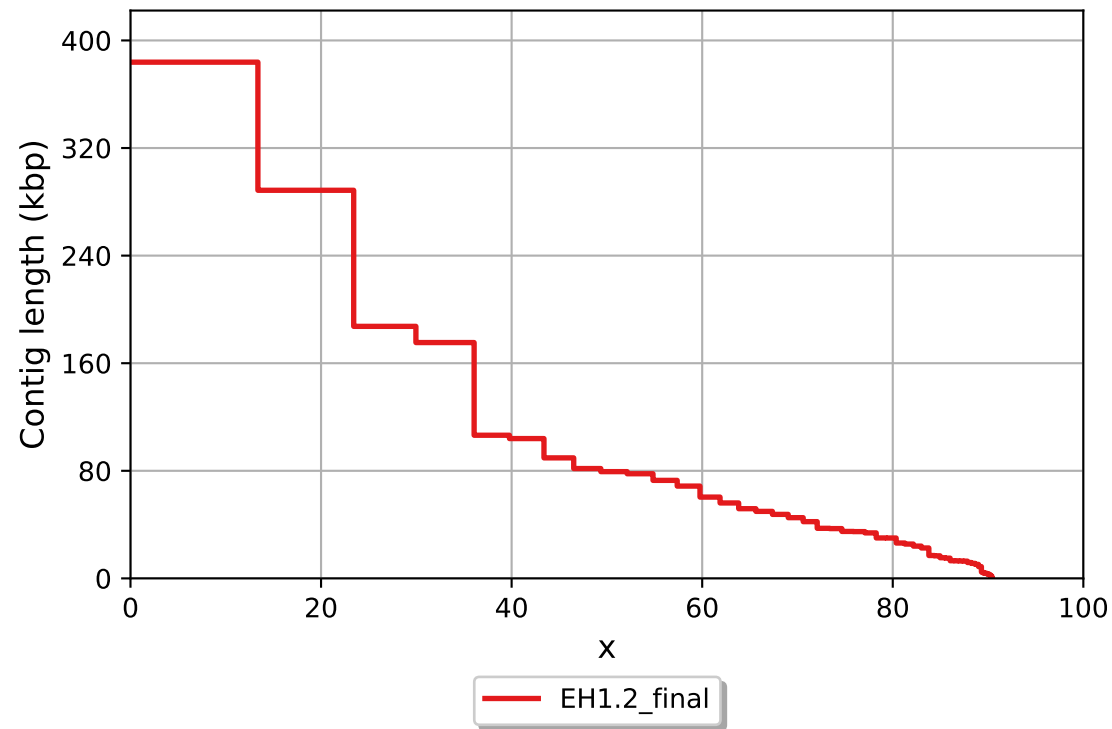

Supplement: Supplementary file 1 [file microorganisms-14-00921-s001.zip › Supplementary File S1/QUAST/EH1.2/aligned_stats/NGAx_plot.pdf]

Cumulative length

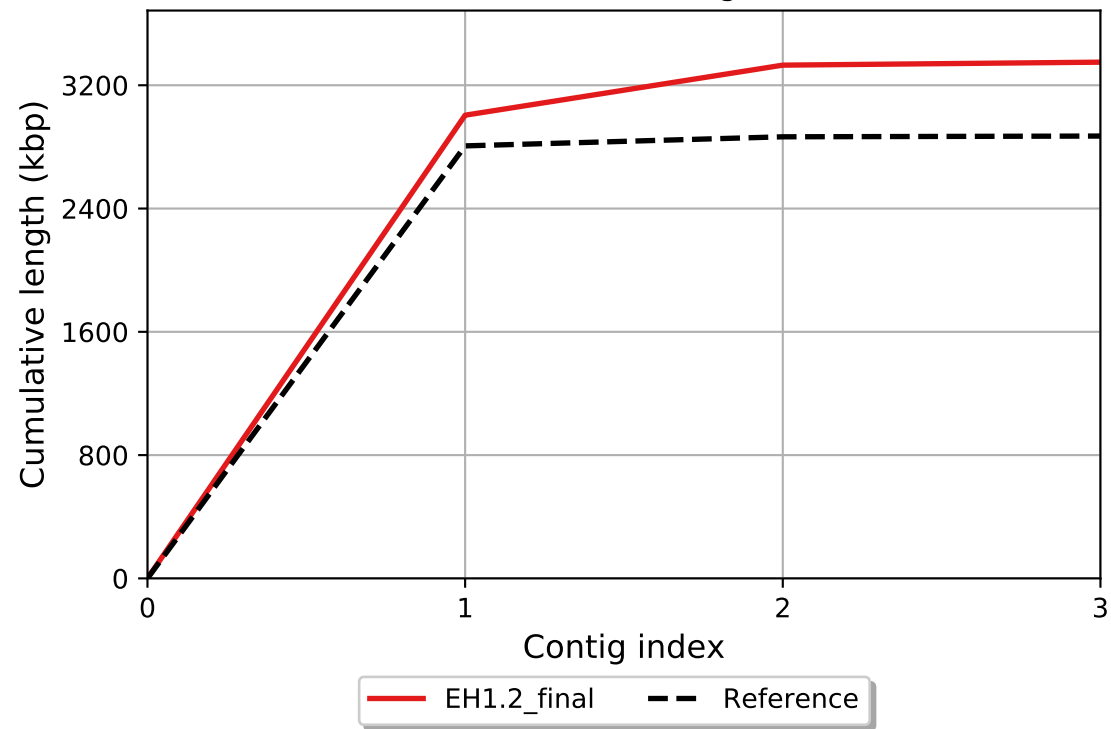

Supplement: Supplementary file 1 [file microorganisms-14-00921-s001.zip › Supplementary File S1/QUAST/EH1.2/basic_stats/cumulative_plot.pdf]

EH1.2\_final GC content

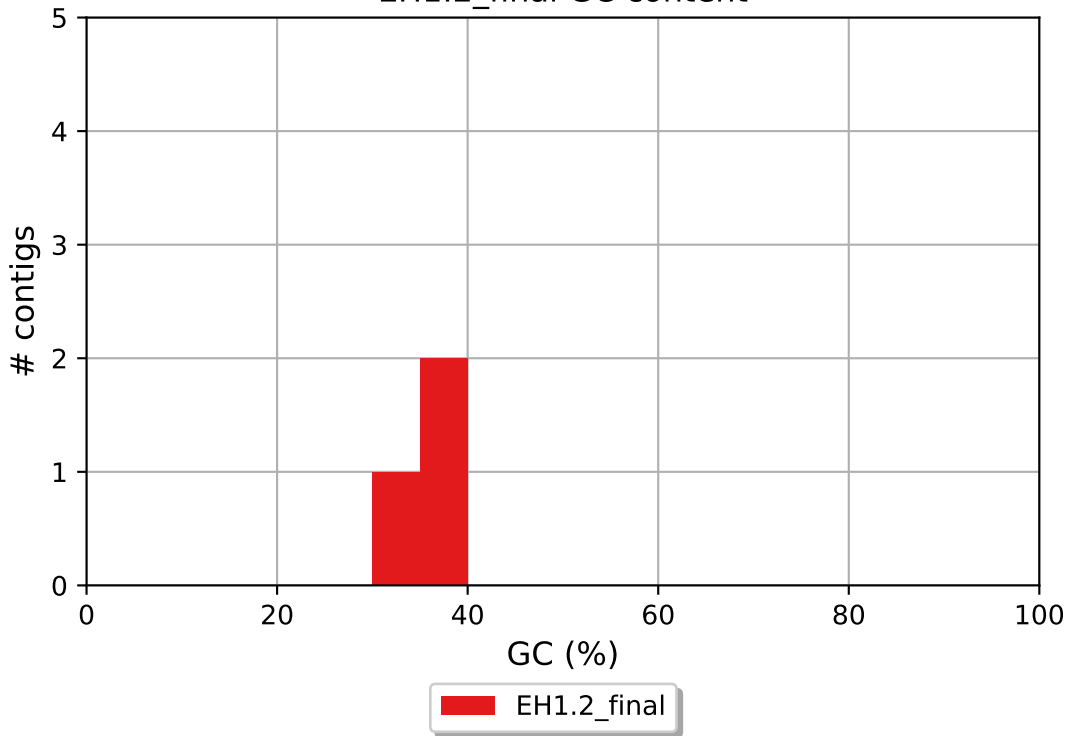

Supplement: Supplementary file 1 [file microorganisms-14-00921-s001.zip › Supplementary File S1/QUAST/EH1.2/basic_stats/EH1.2_final_GC_content_plot.pdf]

# GC content

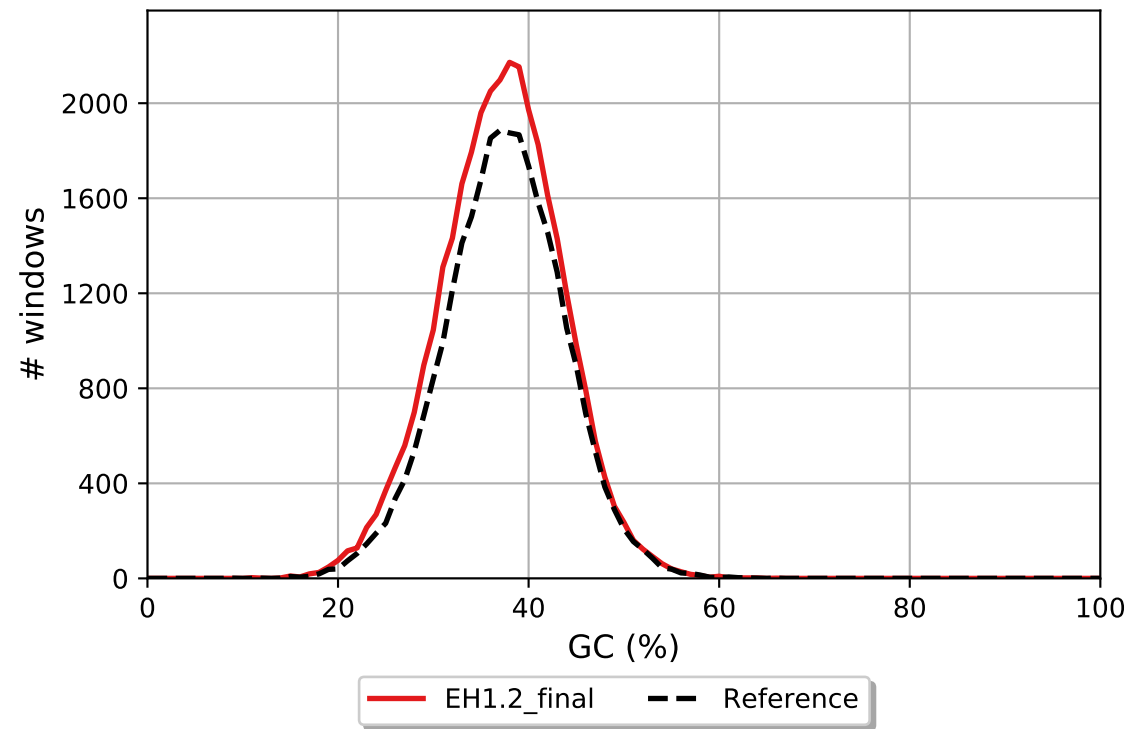

Supplement: Supplementary file 1 [file microorganisms-14-00921-s001.zip › Supplementary File S1/QUAST/EH1.2/basic_stats/GC_content_plot.pdf]

NGx

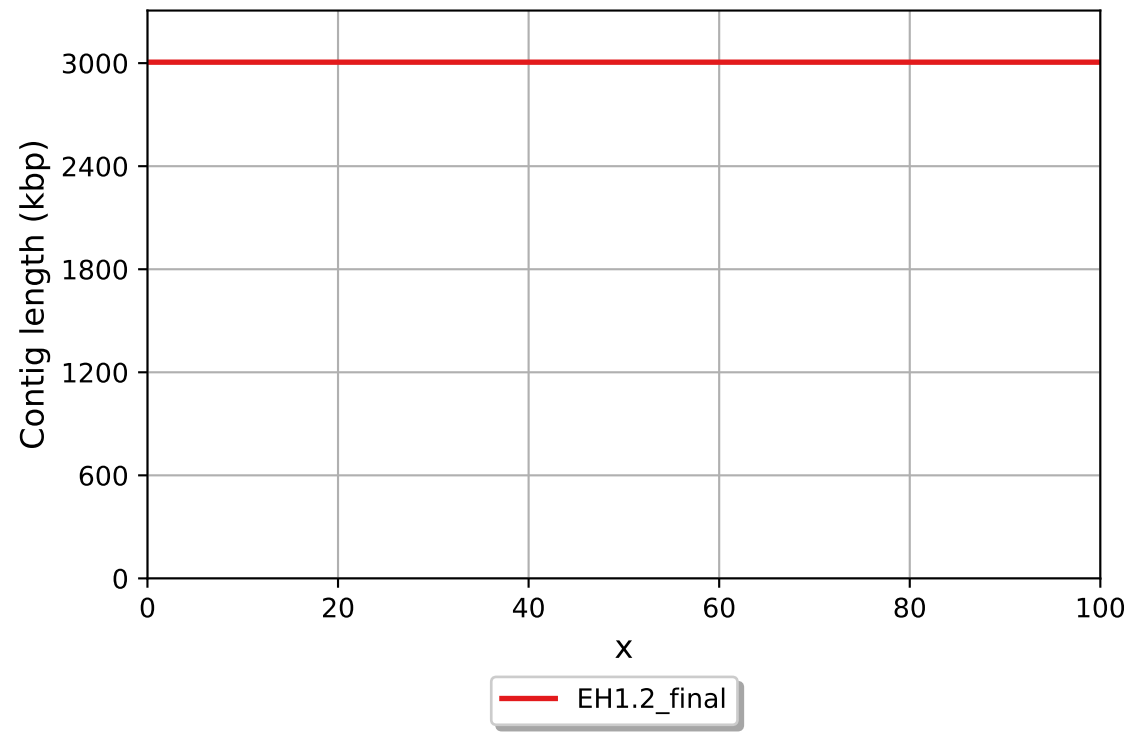

Supplement: Supplementary file 1 [file microorganisms-14-00921-s001.zip › Supplementary File S1/QUAST/EH1.2/basic_stats/NGx_plot.pdf]

Nx

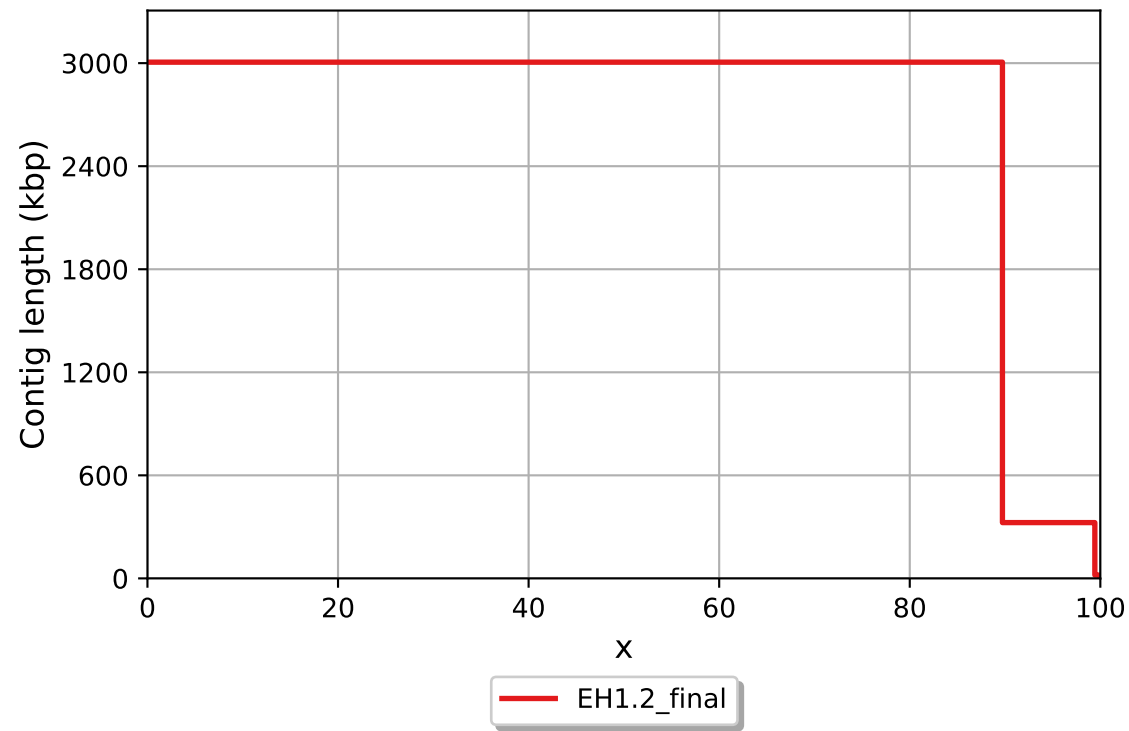

Supplement: Supplementary file 1 [file microorganisms-14-00921-s001.zip › Supplementary File S1/QUAST/EH1.2/basic_stats/Nx_plot.pdf]

FRCurve (misassemblies)

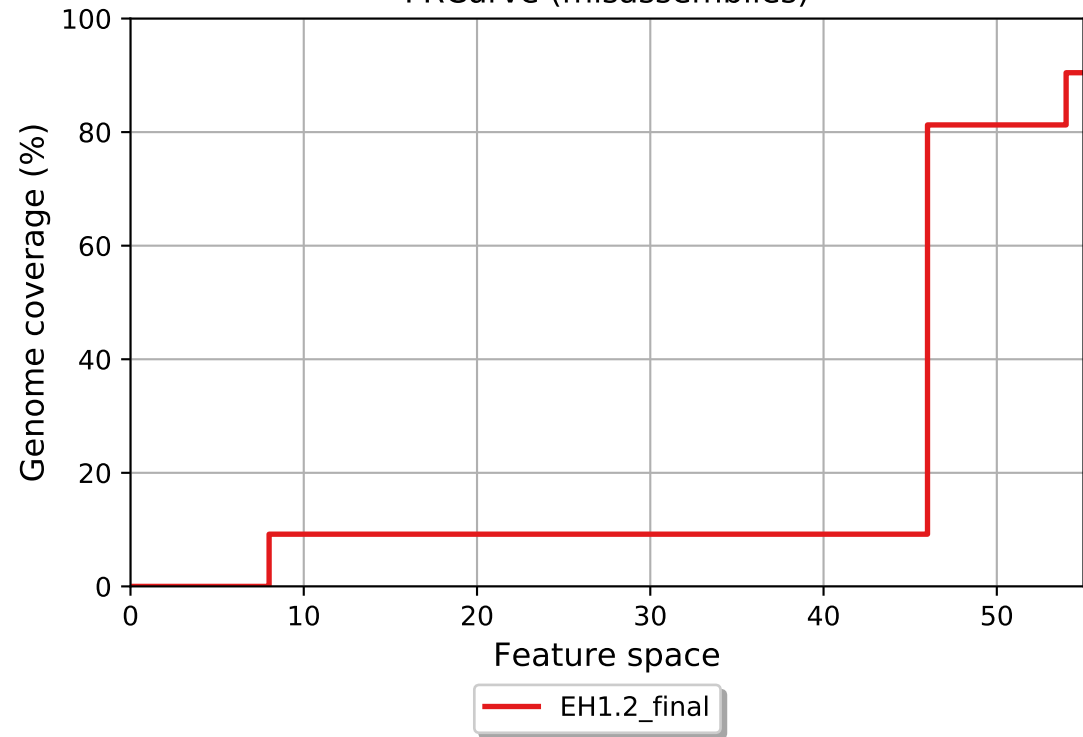

Supplement: Supplementary file 1 [file microorganisms-14-00921-s001.zip › Supplementary File S1/QUAST/EH1.2/contigs_reports/misassemblies_frcurve_plot.pdf]

# Misassemblies

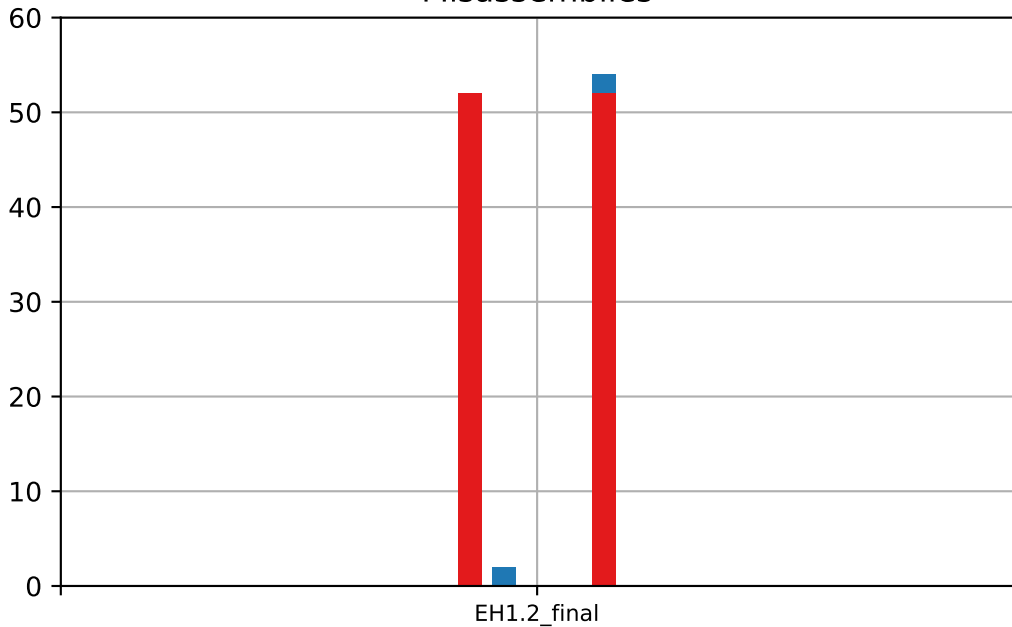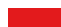

# relocations

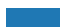

# translocations

Supplement: Supplementary file 1 [file microorganisms-14-00921-s001.zip › Supplementary File S1/QUAST/EH1.2/contigs_reports/misassemblies_plot.pdf]

Cumulative length (aligned contigs)

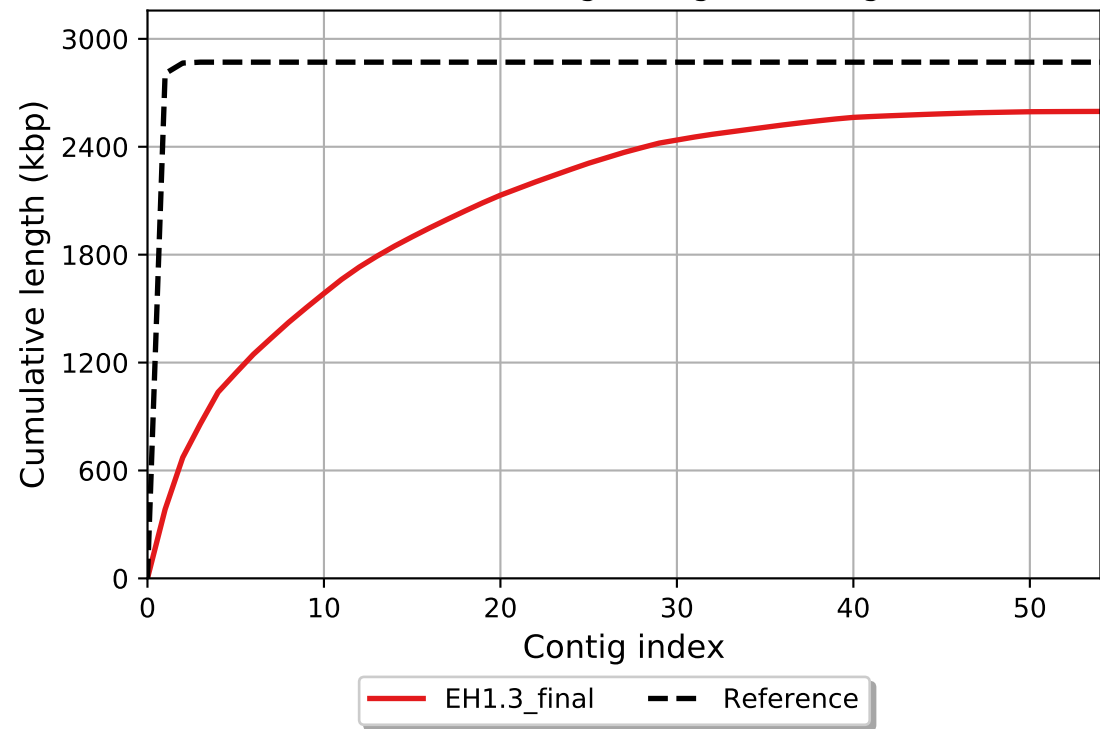

Supplement: Supplementary file 1 [file microorganisms-14-00921-s001.zip › Supplementary File S1/QUAST/EH1.3/aligned_stats/cumulative_plot.pdf]

NAx

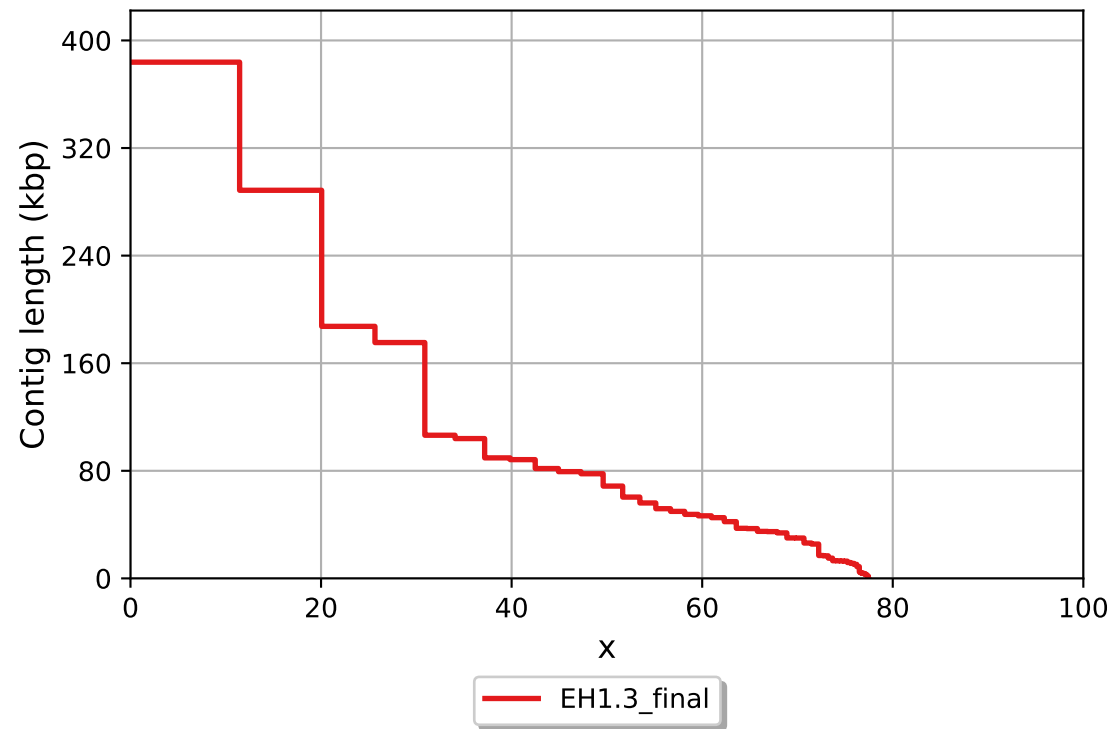

Supplement: Supplementary file 1 [file microorganisms-14-00921-s001.zip › Supplementary File S1/QUAST/EH1.3/aligned_stats/NAx_plot.pdf]

# NGAx

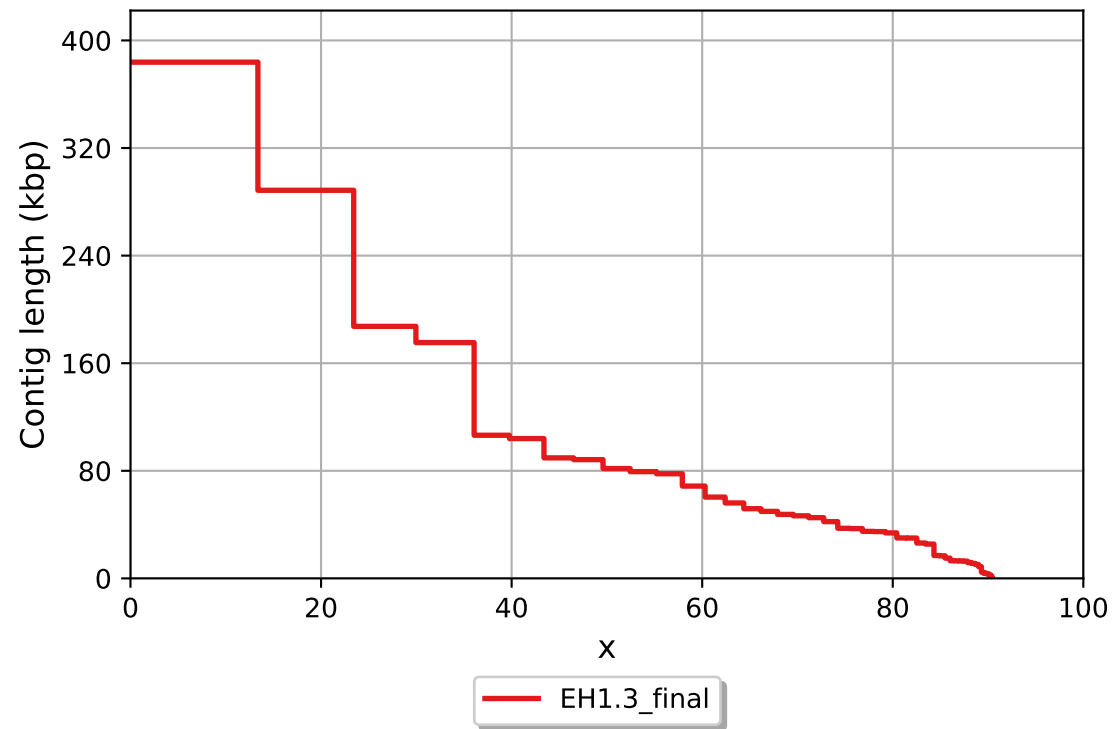

Supplement: Supplementary file 1 [file microorganisms-14-00921-s001.zip › Supplementary File S1/QUAST/EH1.3/aligned_stats/NGAx_plot.pdf]

Cumulative length

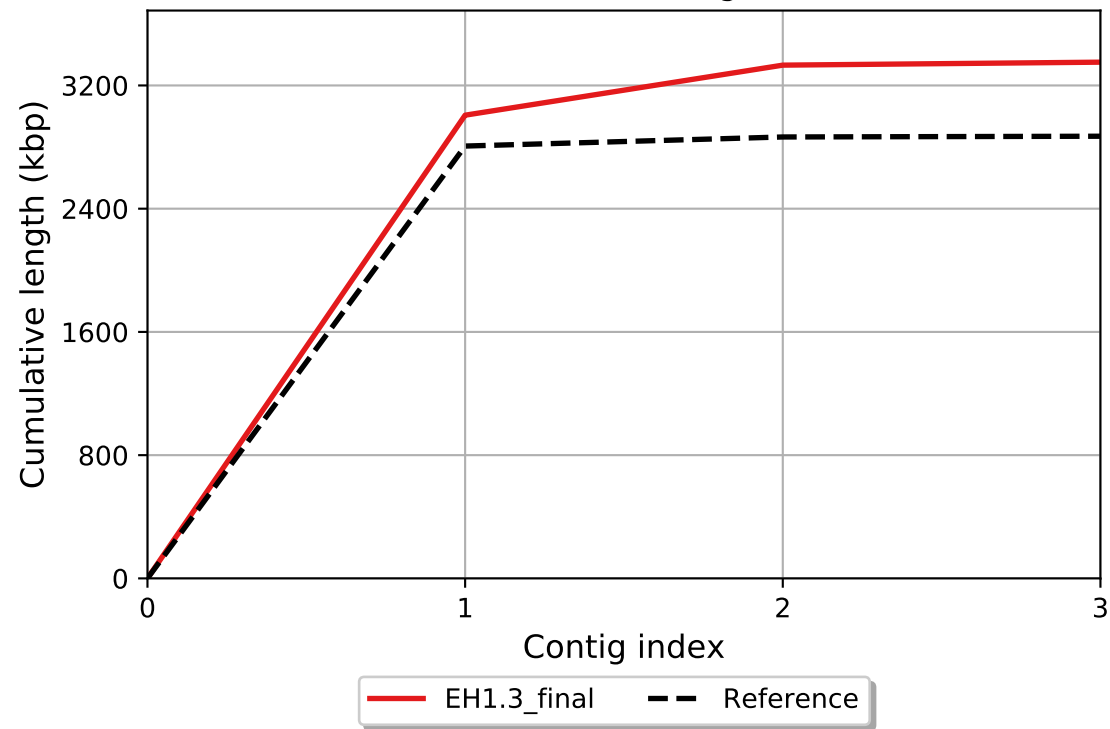

Supplement: Supplementary file 1 [file microorganisms-14-00921-s001.zip › Supplementary File S1/QUAST/EH1.3/basic_stats/cumulative_plot.pdf]

EH1.3\_final GC content

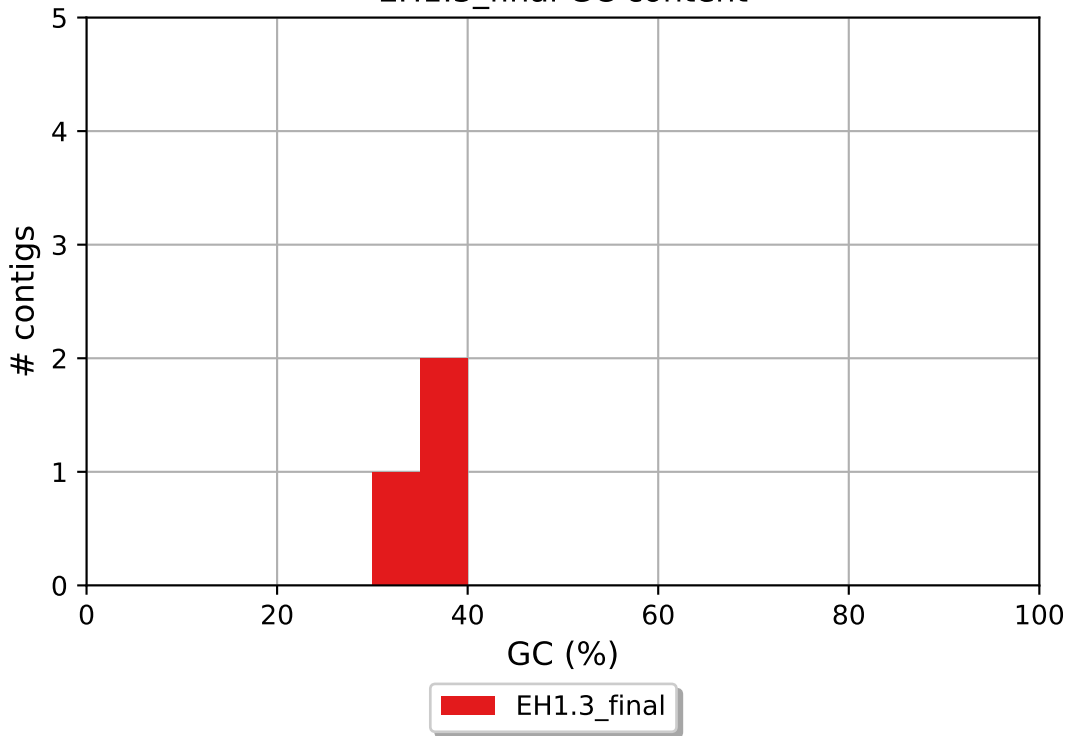

Supplement: Supplementary file 1 [file microorganisms-14-00921-s001.zip › Supplementary File S1/QUAST/EH1.3/basic_stats/EH1.3_final_GC_content_plot.pdf]

# GC content

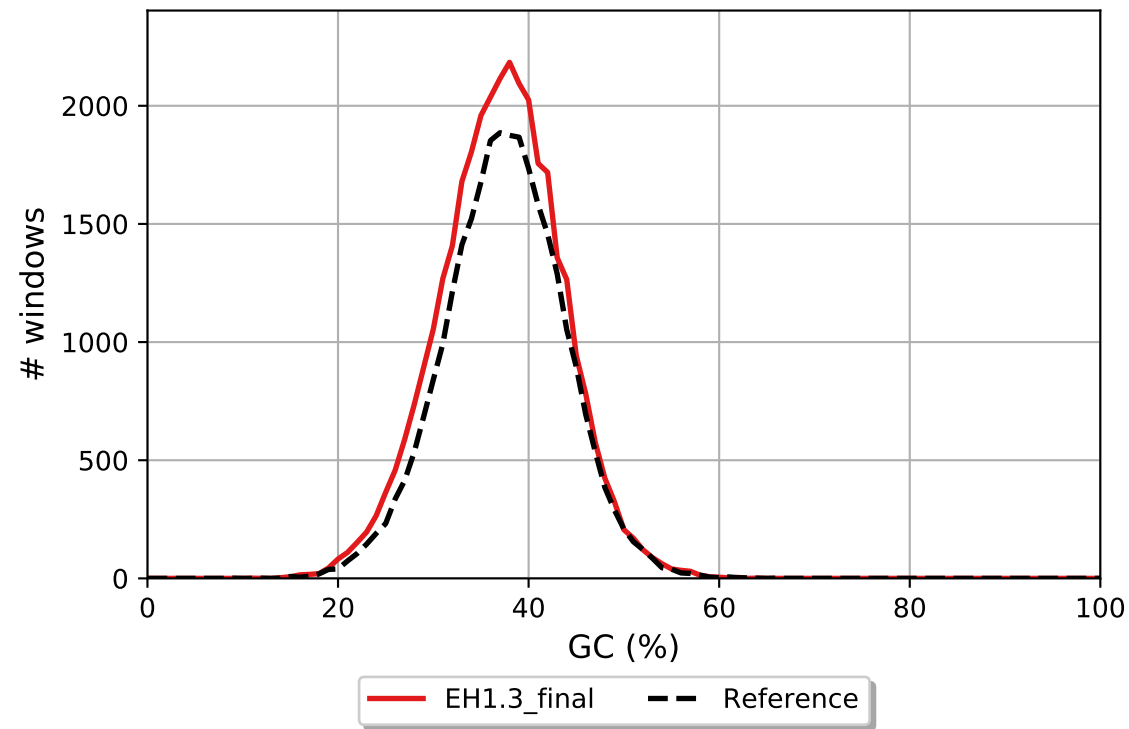

Supplement: Supplementary file 1 [file microorganisms-14-00921-s001.zip › Supplementary File S1/QUAST/EH1.3/basic_stats/GC_content_plot.pdf]

NGx

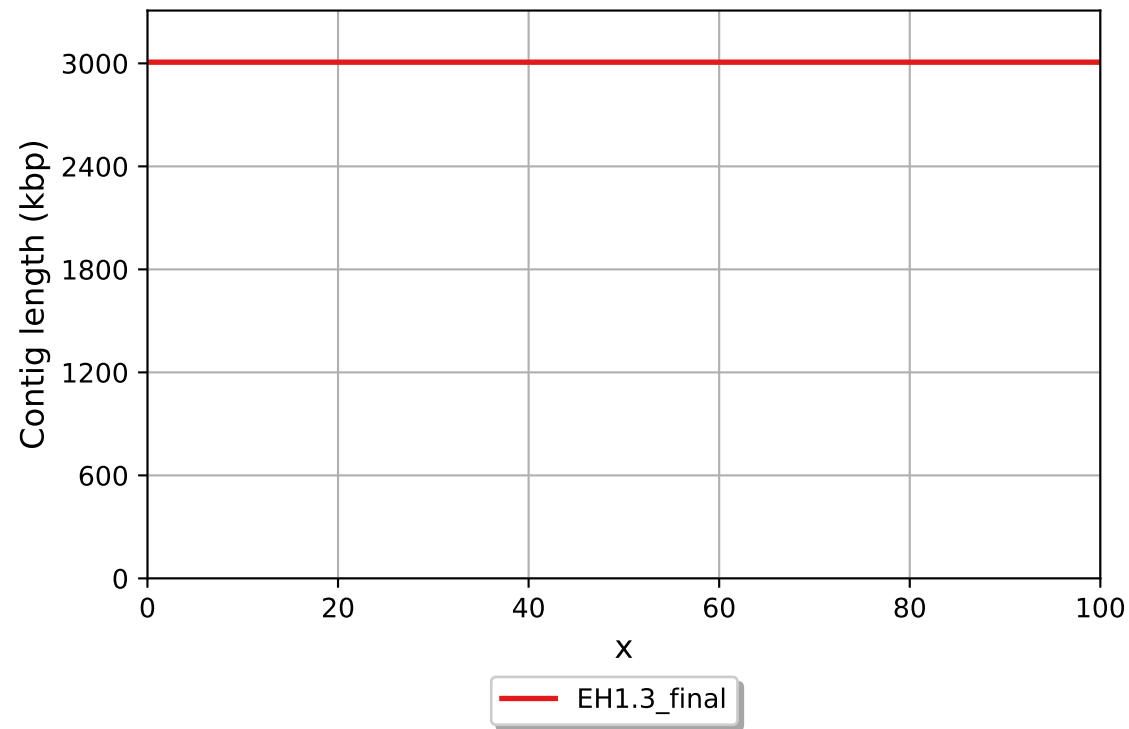

Supplement: Supplementary file 1 [file microorganisms-14-00921-s001.zip › Supplementary File S1/QUAST/EH1.3/basic_stats/NGx_plot.pdf]

Nx

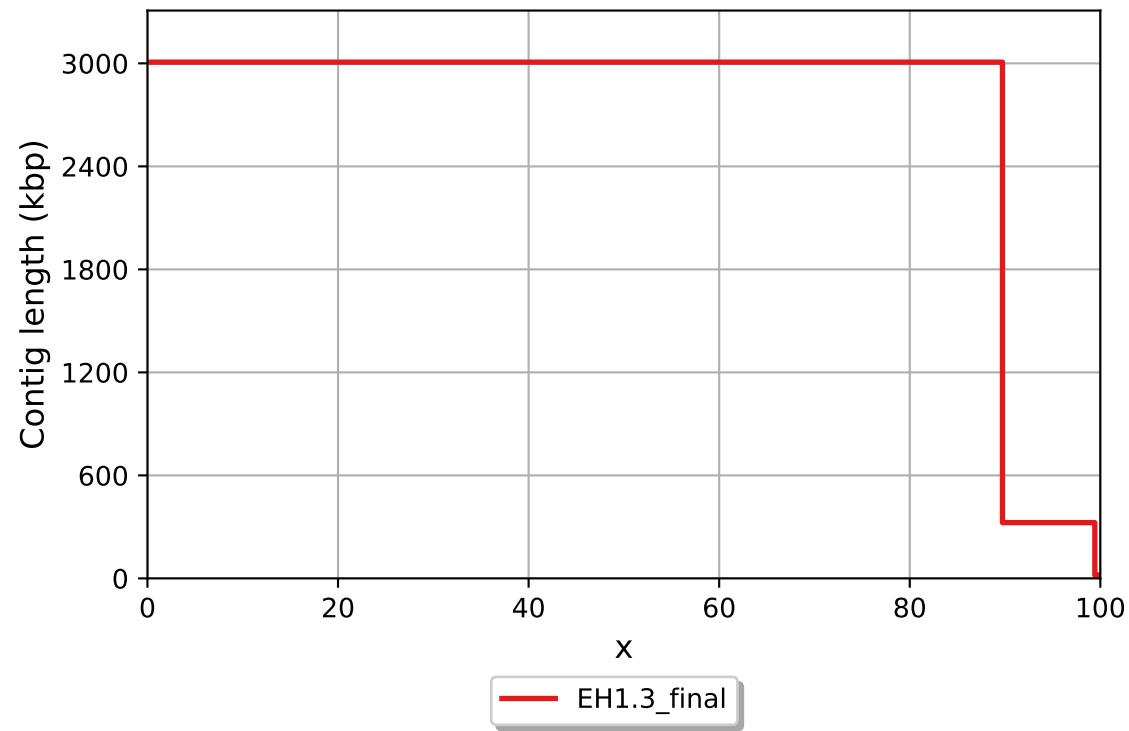

Supplement: Supplementary file 1 [file microorganisms-14-00921-s001.zip › Supplementary File S1/QUAST/EH1.3/basic_stats/Nx_plot.pdf]

FRCurve (misassemblies)

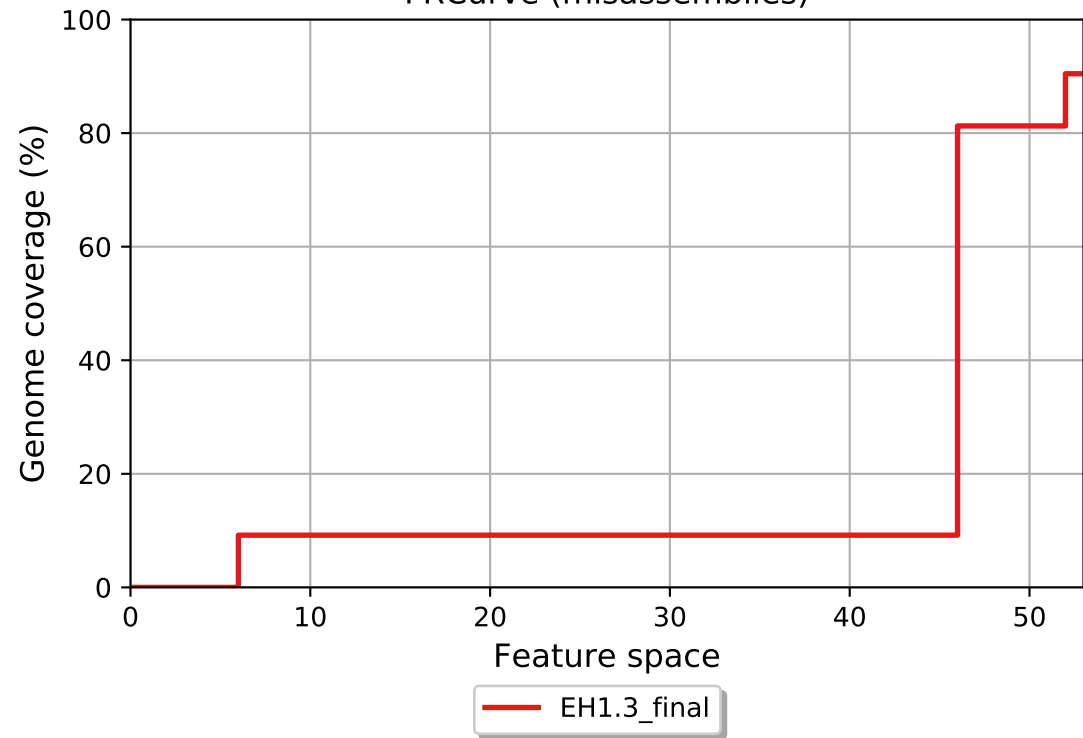

Supplement: Supplementary file 1 [file microorganisms-14-00921-s001.zip › Supplementary File S1/QUAST/EH1.3/contigs_reports/misassemblies_frcurve_plot.pdf]

# Misassemblies

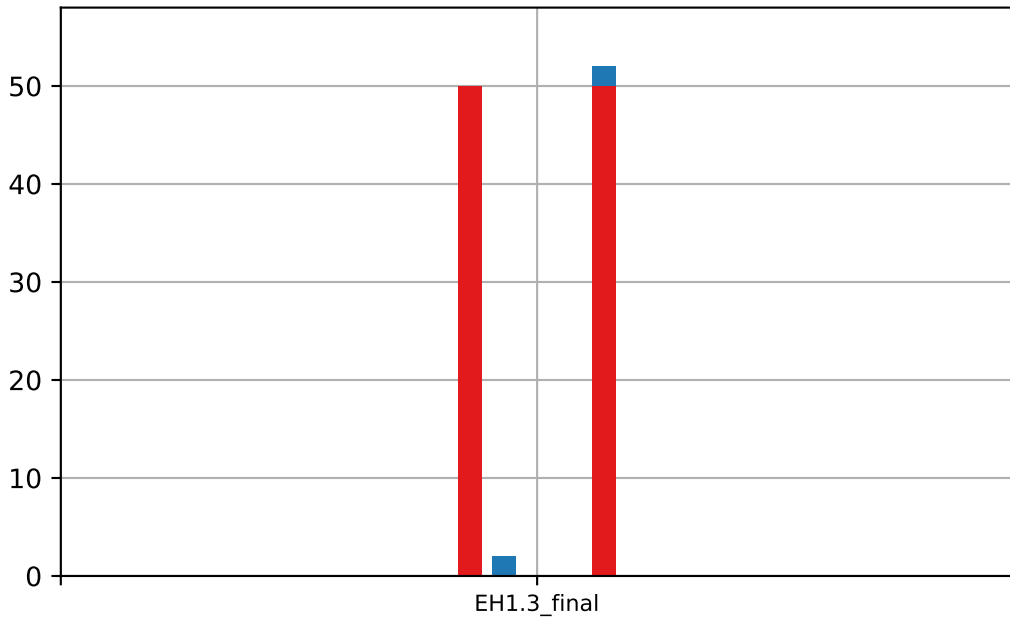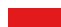

# relocations

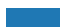

# translocations

Supplement: Supplementary file 1 [file microorganisms-14-00921-s001.zip › Supplementary File S1/QUAST/EH1.3/contigs_reports/misassemblies_plot.pdf]

Cumulative length (aligned contigs)

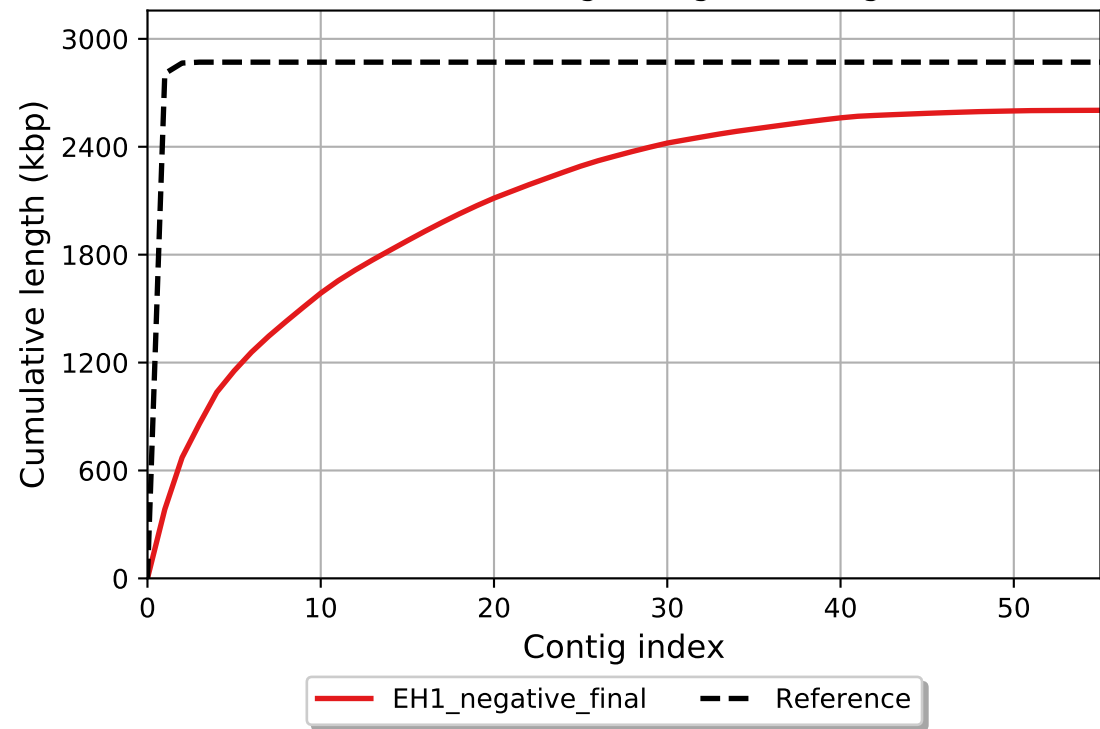

Supplement: Supplementary file 1 [file microorganisms-14-00921-s001.zip › Supplementary File S1/QUAST/EH1_negative/aligned_stats/cumulative_plot.pdf]

NAx

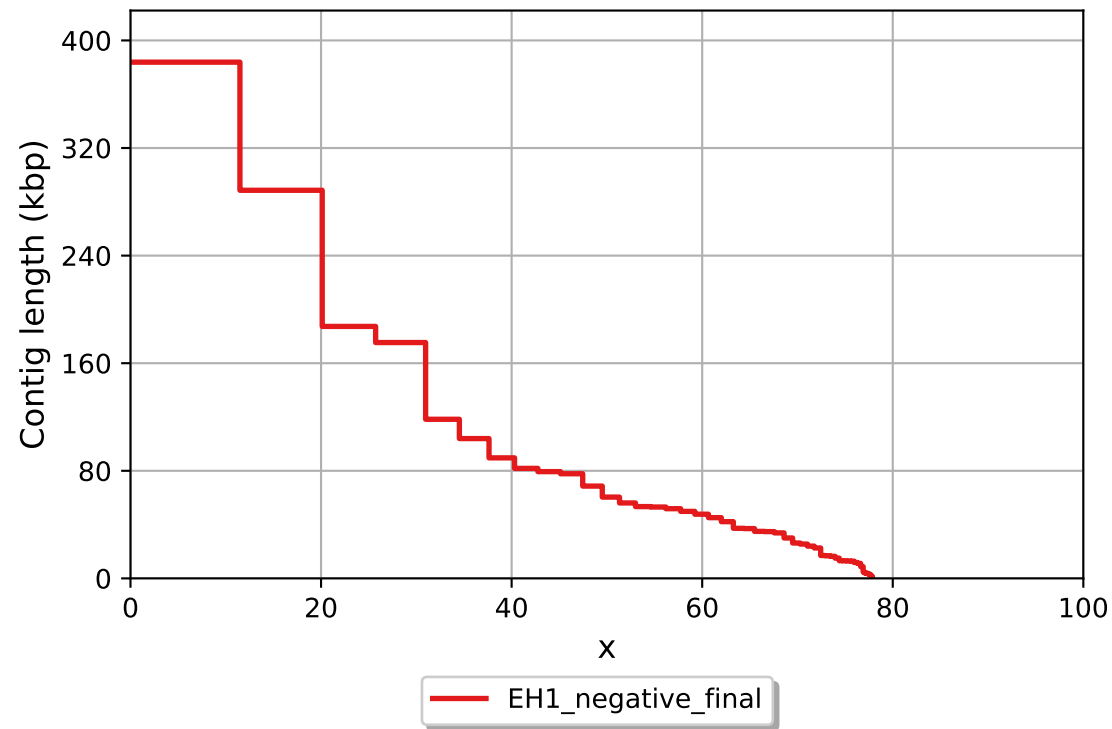

Supplement: Supplementary file 1 [file microorganisms-14-00921-s001.zip › Supplementary File S1/QUAST/EH1_negative/aligned_stats/NAx_plot.pdf]

# NGAx

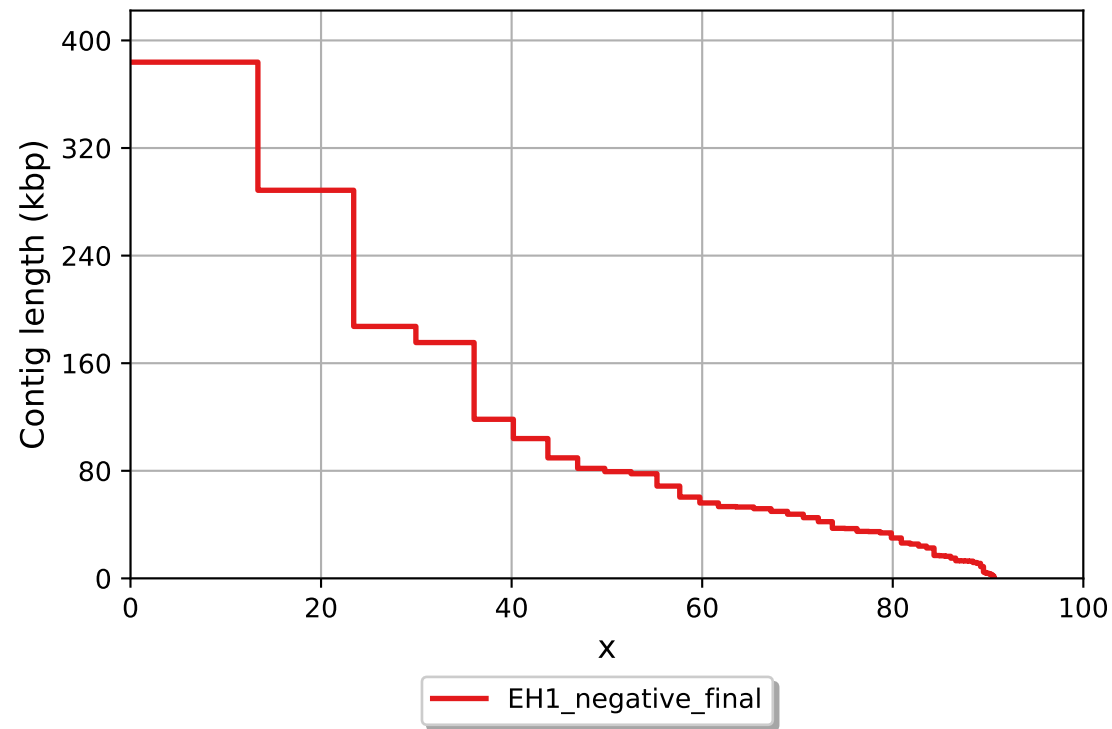

Supplement: Supplementary file 1 [file microorganisms-14-00921-s001.zip › Supplementary File S1/QUAST/EH1_negative/aligned_stats/NGAx_plot.pdf]

Cumulative length

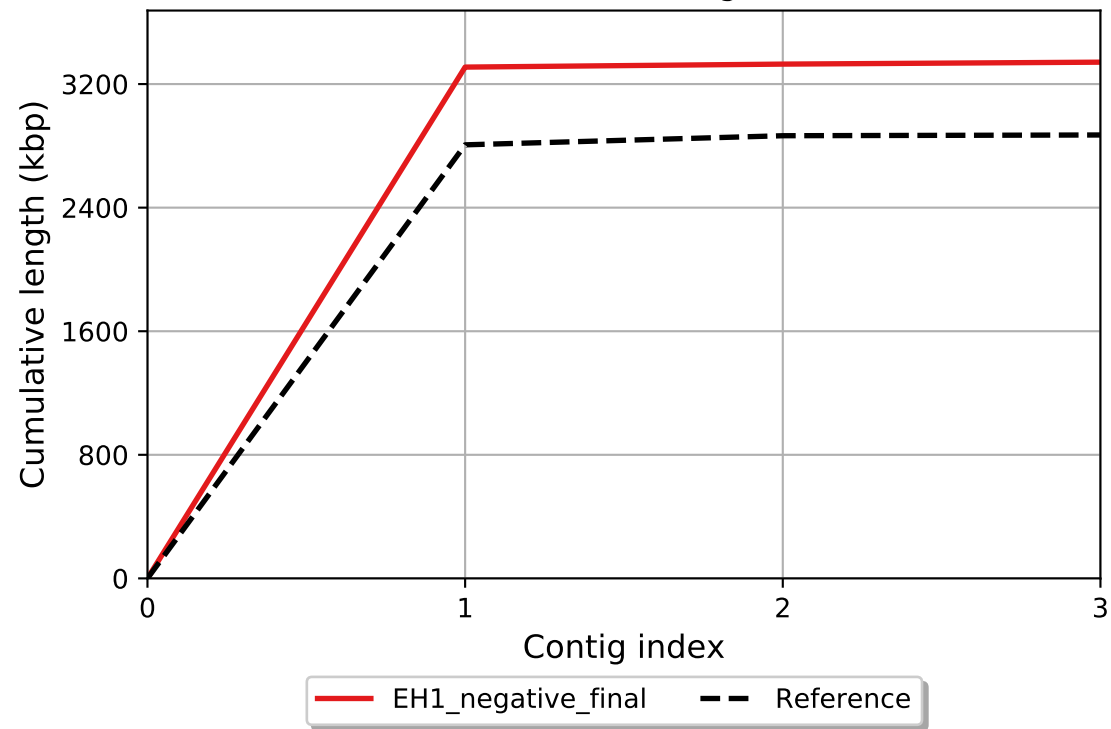

Supplement: Supplementary file 1 [file microorganisms-14-00921-s001.zip › Supplementary File S1/QUAST/EH1_negative/basic_stats/cumulative_plot.pdf]

EH1\_negative\_final GC content

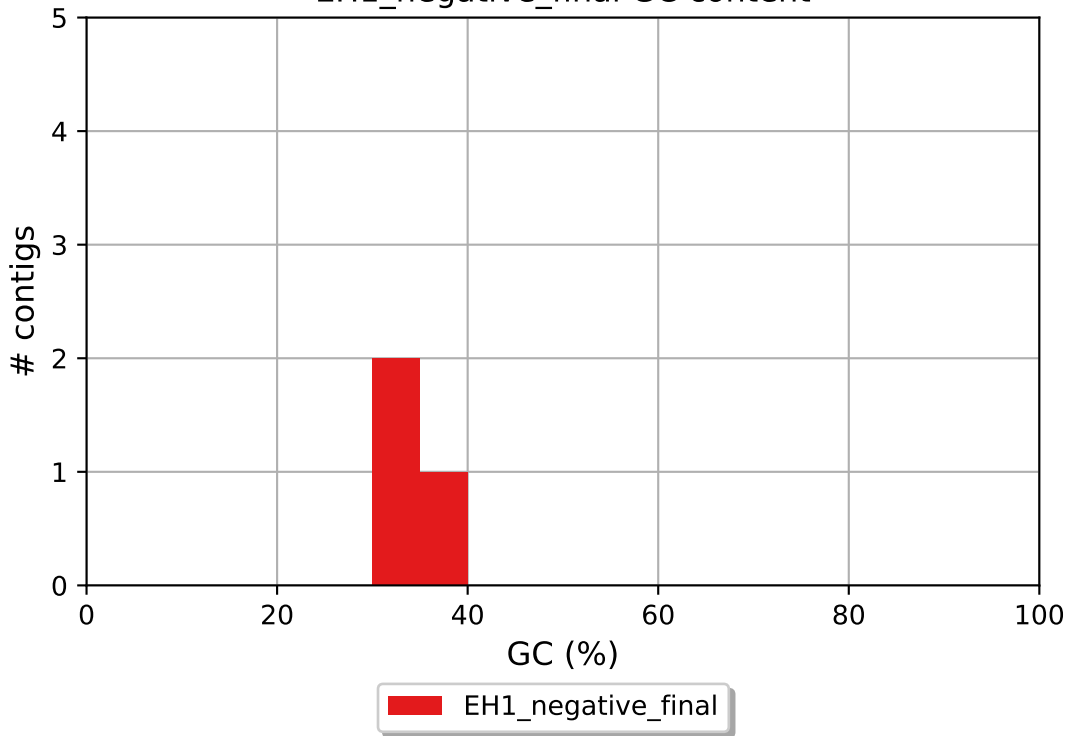

Supplement: Supplementary file 1 [file microorganisms-14-00921-s001.zip › Supplementary File S1/QUAST/EH1_negative/basic_stats/EH1_negative_final_GC_content_plot.pdf]

GC content

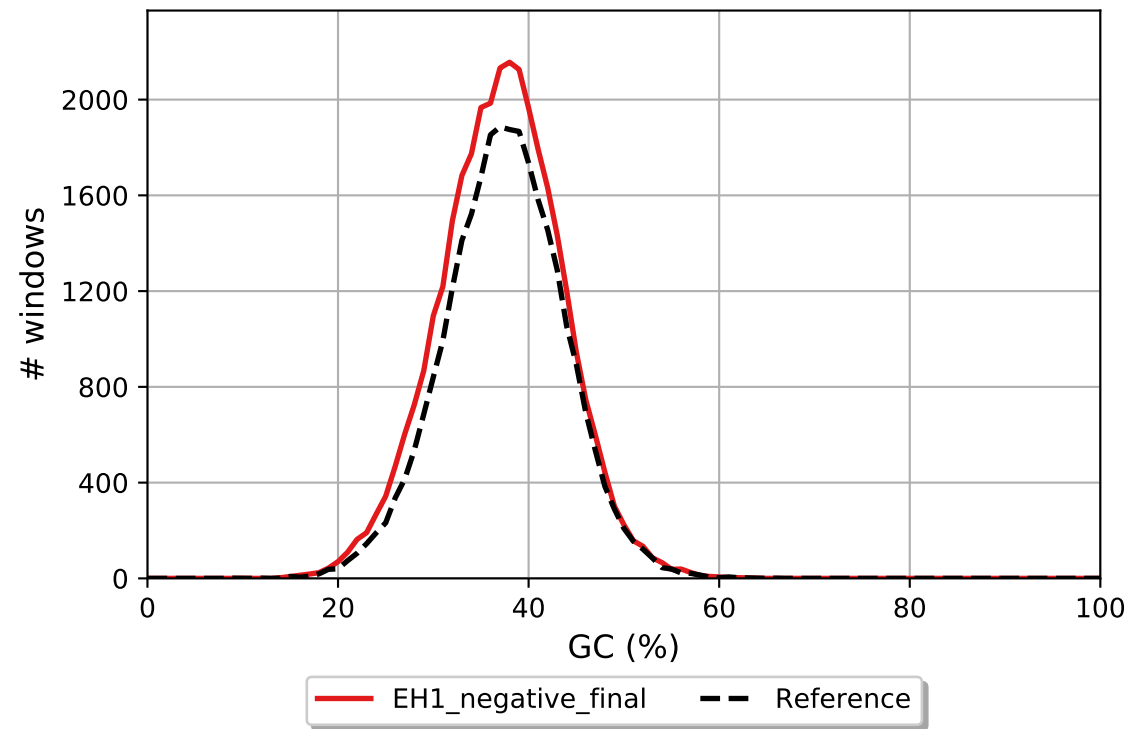

Supplement: Supplementary file 1 [file microorganisms-14-00921-s001.zip › Supplementary File S1/QUAST/EH1_negative/basic_stats/GC_content_plot.pdf]

NGx

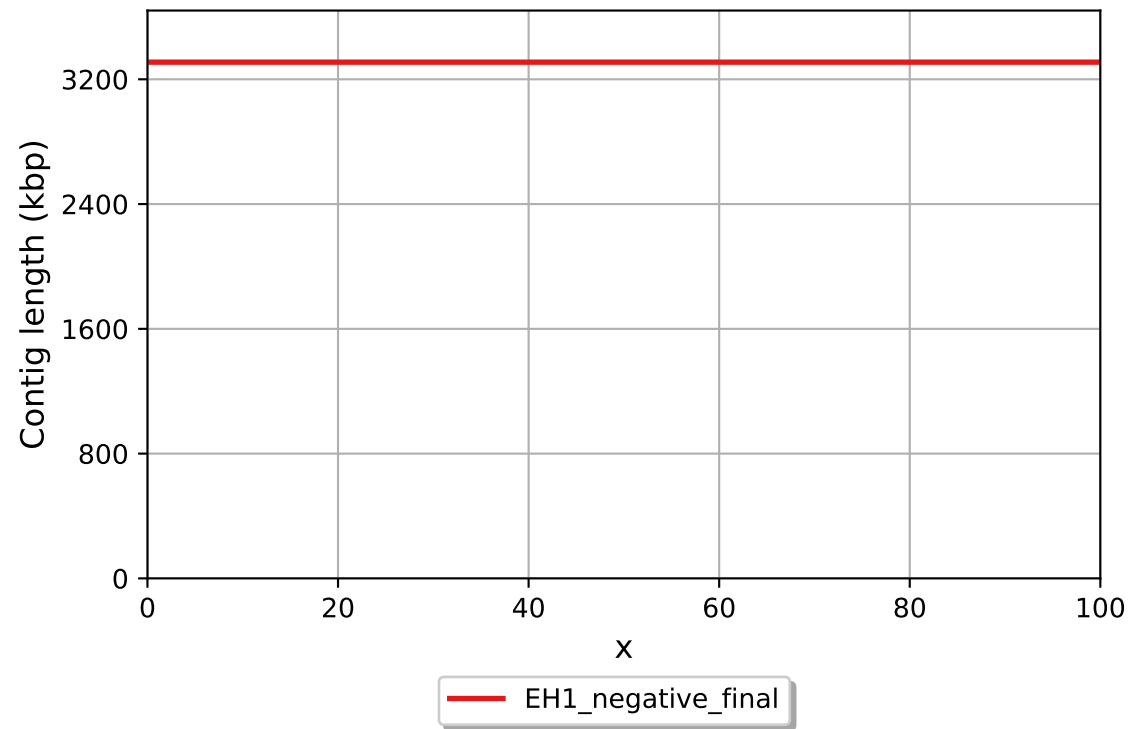

Supplement: Supplementary file 1 [file microorganisms-14-00921-s001.zip › Supplementary File S1/QUAST/EH1_negative/basic_stats/NGx_plot.pdf]

Nx

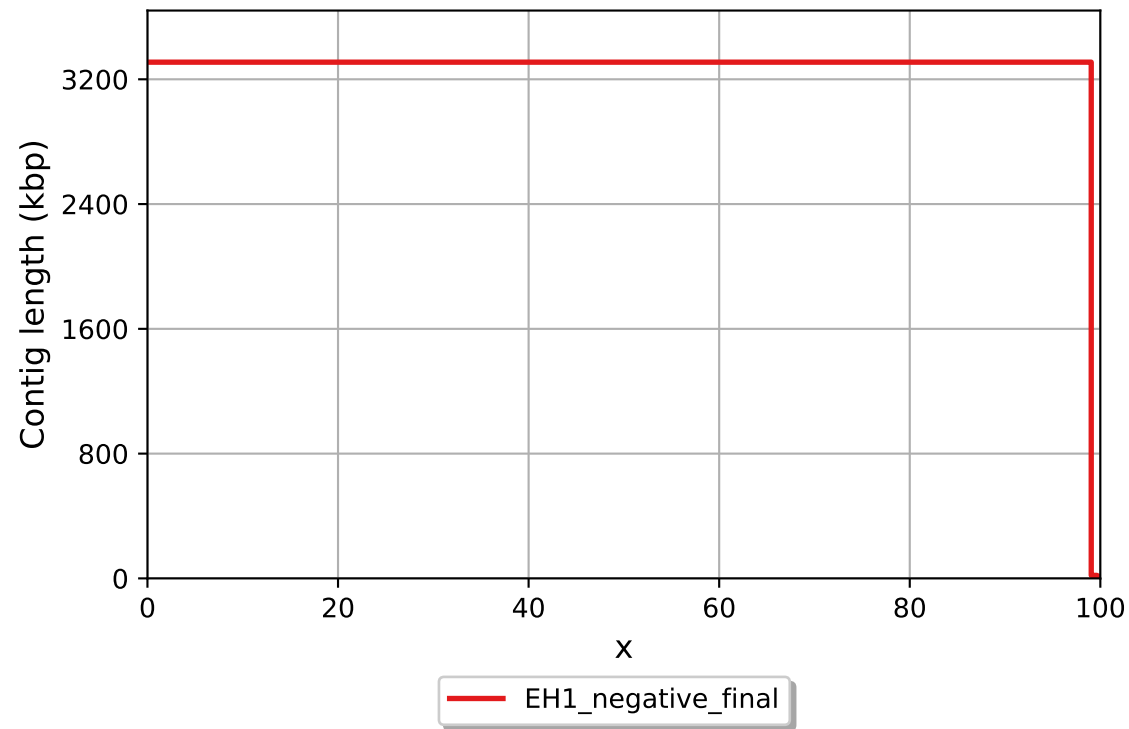

Supplement: Supplementary file 1 [file microorganisms-14-00921-s001.zip › Supplementary File S1/QUAST/EH1_negative/basic_stats/Nx_plot.pdf]

FRCurve (misassemblies)

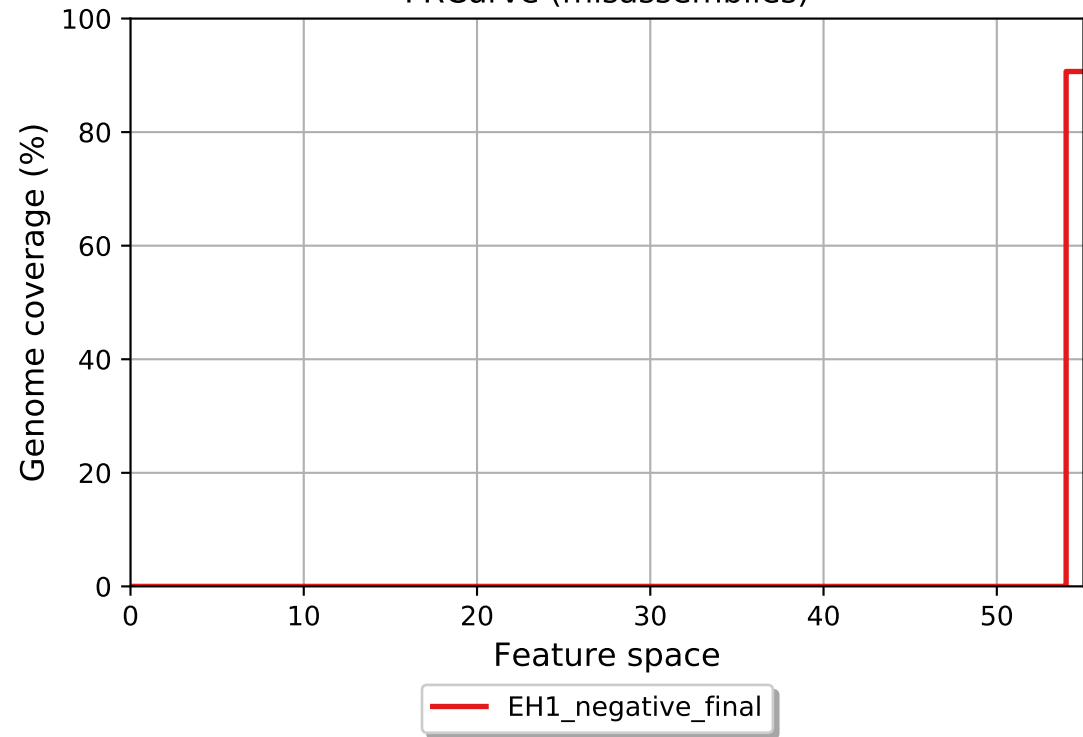

Supplement: Supplementary file 1 [file microorganisms-14-00921-s001.zip › Supplementary File S1/QUAST/EH1_negative/contigs_reports/misassemblies_frcurve_plot.pdf]

# Misassemblies

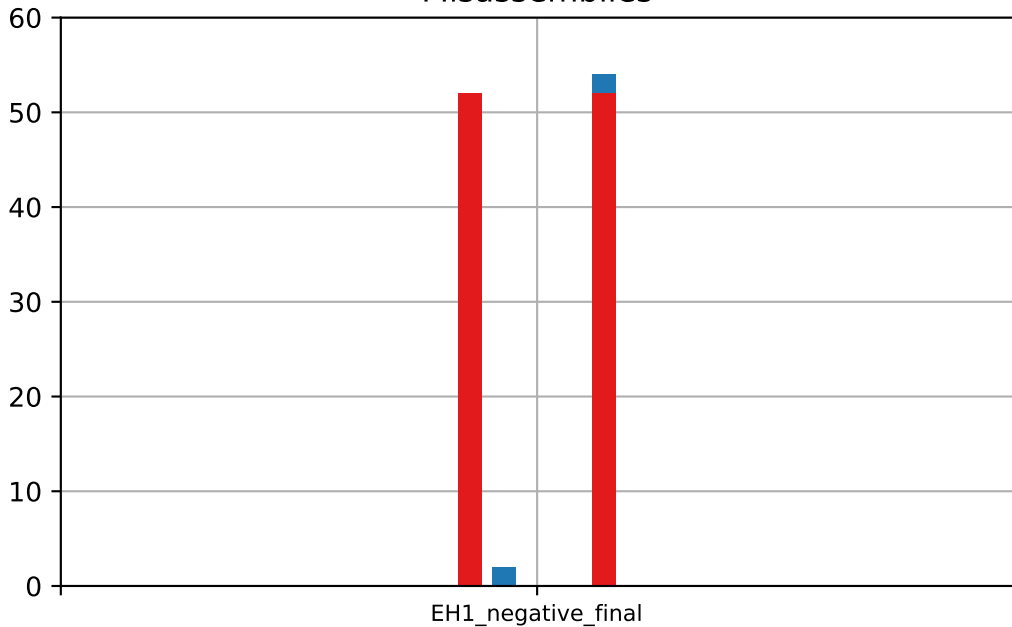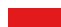

# relocations

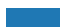

# translocations

Supplement: Supplementary file 1 [file microorganisms-14-00921-s001.zip › Supplementary File S1/QUAST/EH1_negative/contigs_reports/misassemblies_plot.pdf]

Cumulative length (aligned contigs)

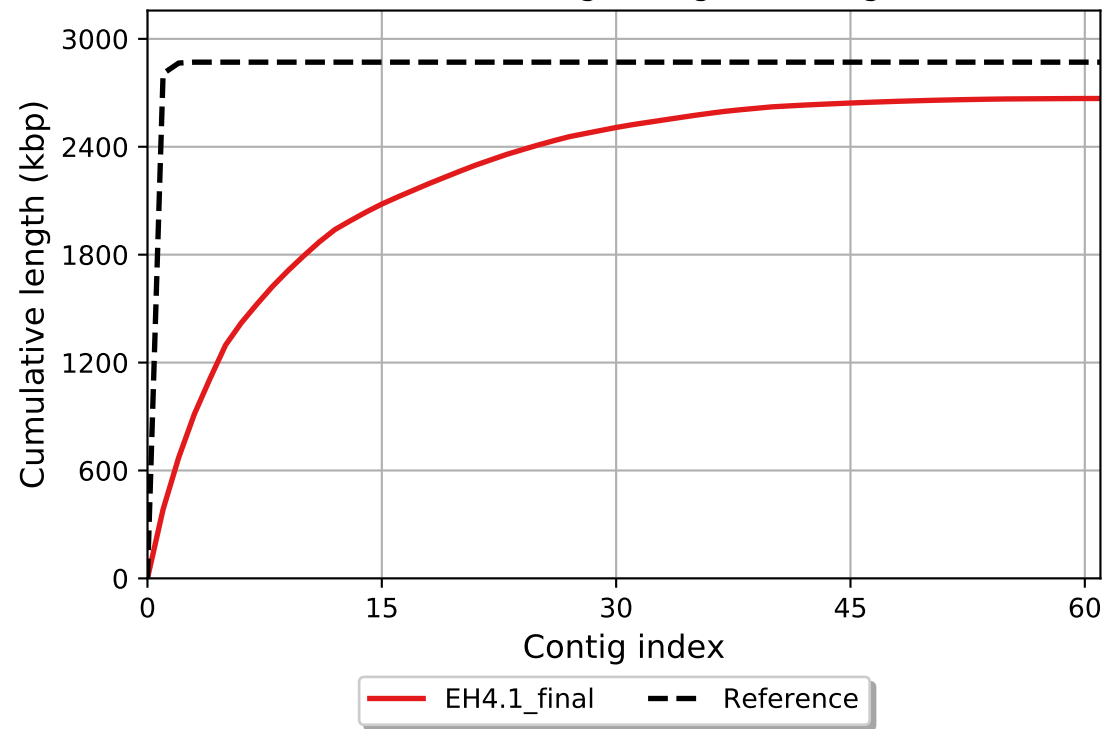

Supplement: Supplementary file 1 [file microorganisms-14-00921-s001.zip › Supplementary File S1/QUAST/EH4.1/aligned_stats/cumulative_plot.pdf]

NAx

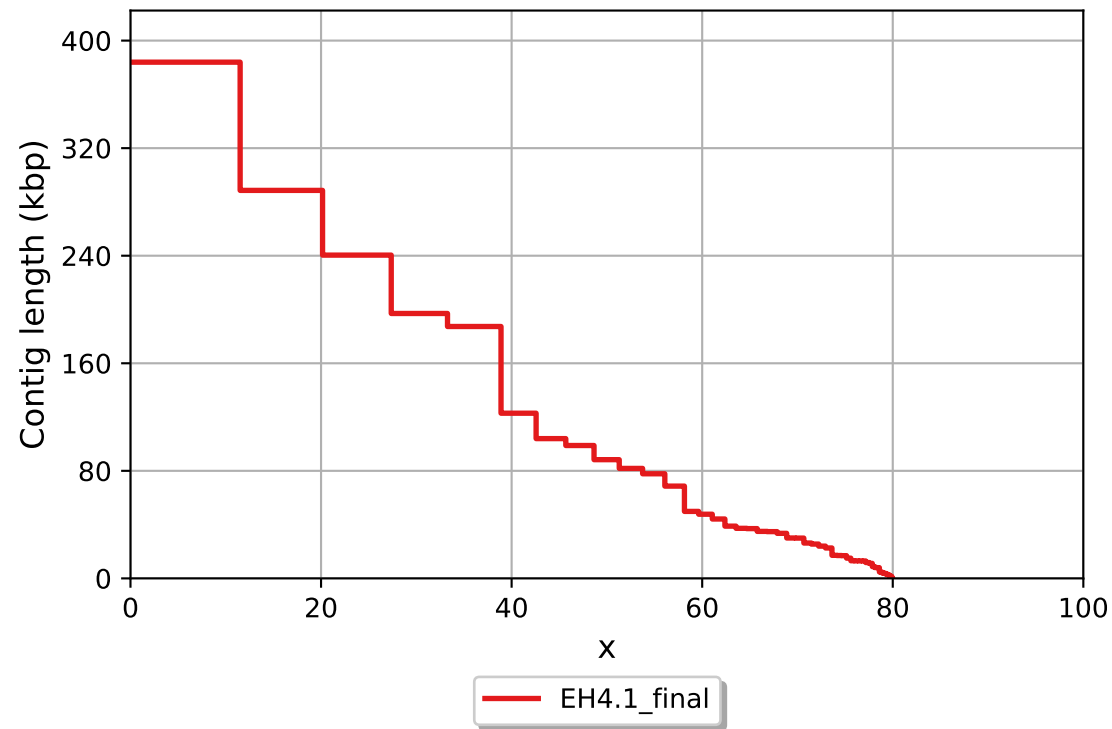

Supplement: Supplementary file 1 [file microorganisms-14-00921-s001.zip › Supplementary File S1/QUAST/EH4.1/aligned_stats/NAx_plot.pdf]

# NGAx

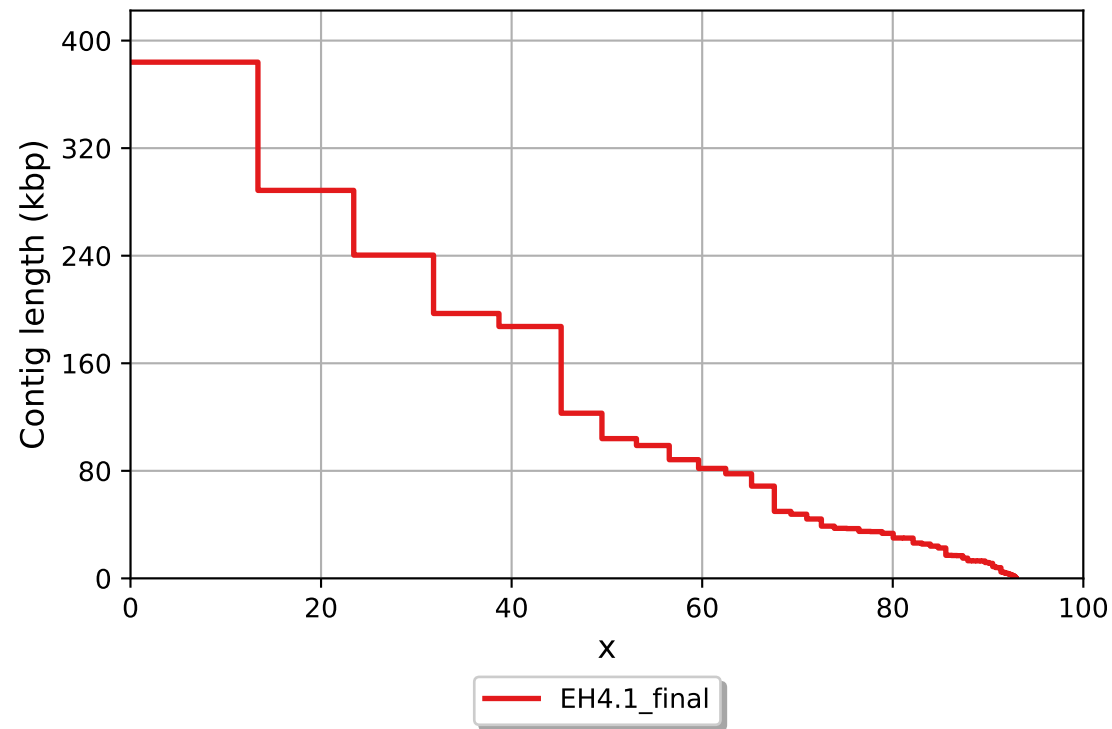

Supplement: Supplementary file 1 [file microorganisms-14-00921-s001.zip › Supplementary File S1/QUAST/EH4.1/aligned_stats/NGAx_plot.pdf]

Cumulative length

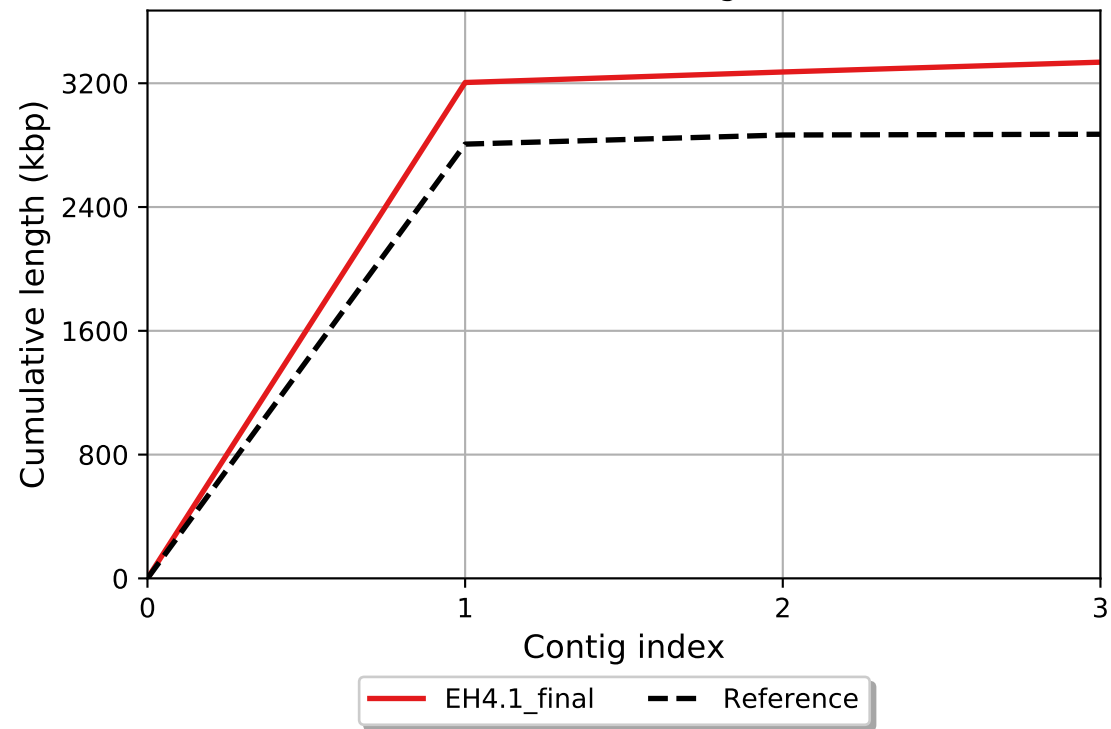

Supplement: Supplementary file 1 [file microorganisms-14-00921-s001.zip › Supplementary File S1/QUAST/EH4.1/basic_stats/cumulative_plot.pdf]

EH4.1\_final GC content

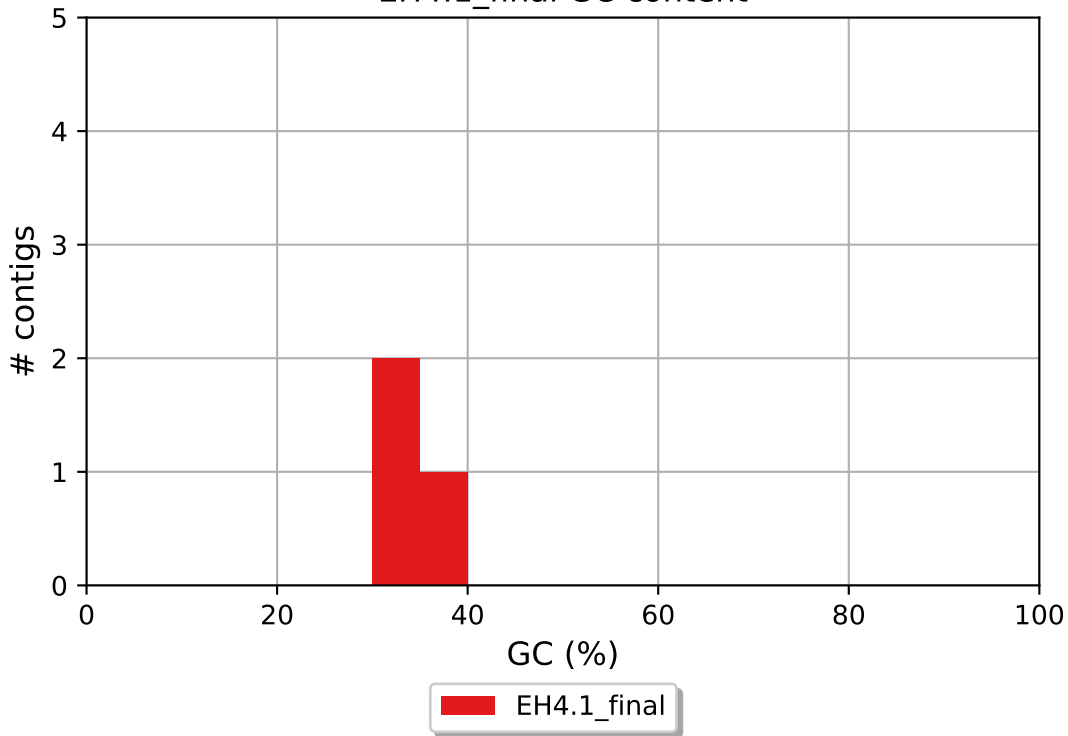

Supplement: Supplementary file 1 [file microorganisms-14-00921-s001.zip › Supplementary File S1/QUAST/EH4.1/basic_stats/EH4.1_final_GC_content_plot.pdf]

# GC content

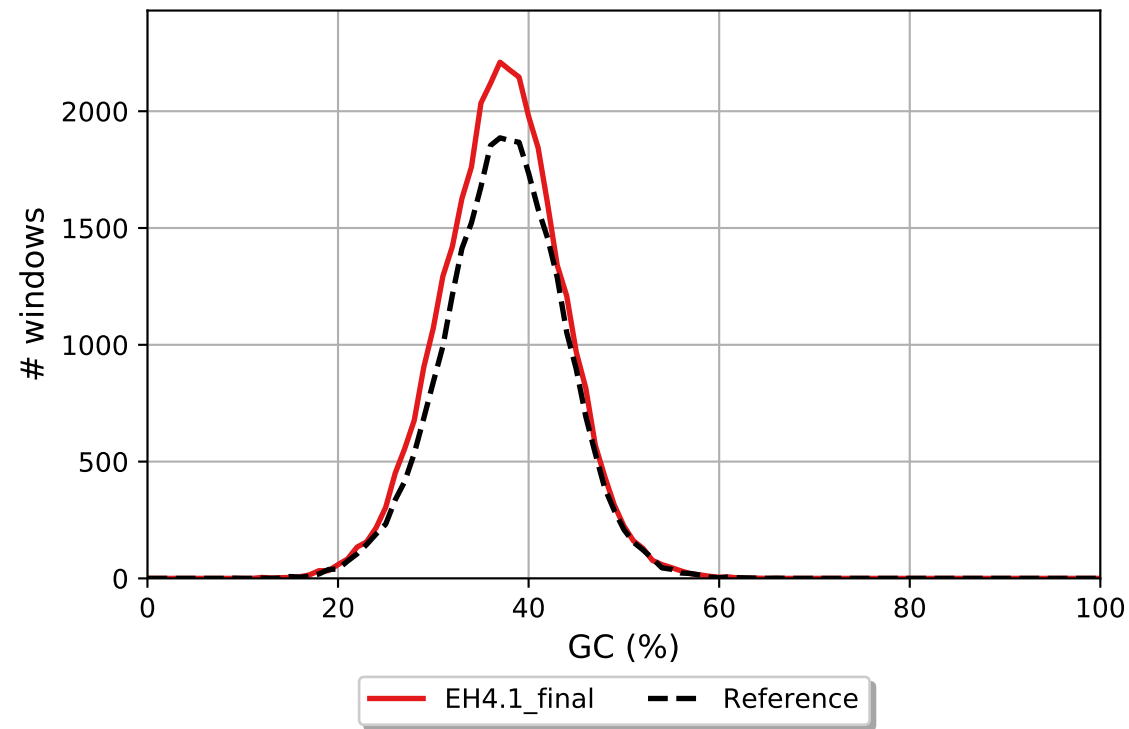

Supplement: Supplementary file 1 [file microorganisms-14-00921-s001.zip › Supplementary File S1/QUAST/EH4.1/basic_stats/GC_content_plot.pdf]

NGx

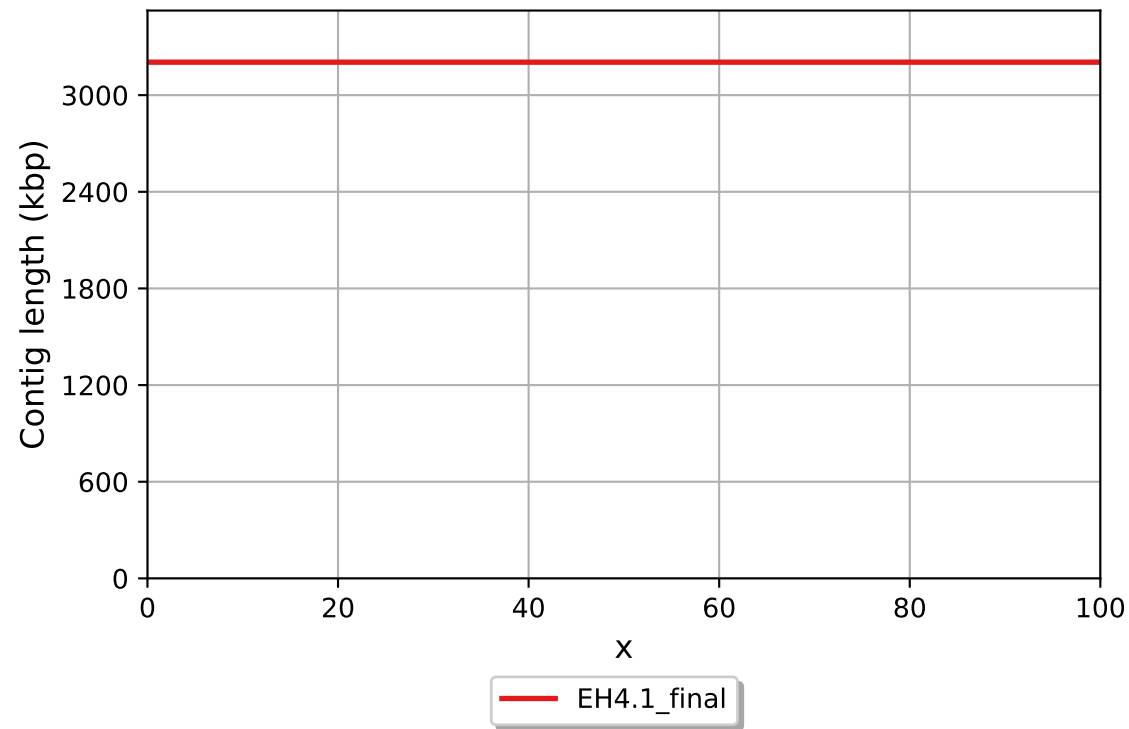

Supplement: Supplementary file 1 [file microorganisms-14-00921-s001.zip › Supplementary File S1/QUAST/EH4.1/basic_stats/NGx_plot.pdf]

Nx

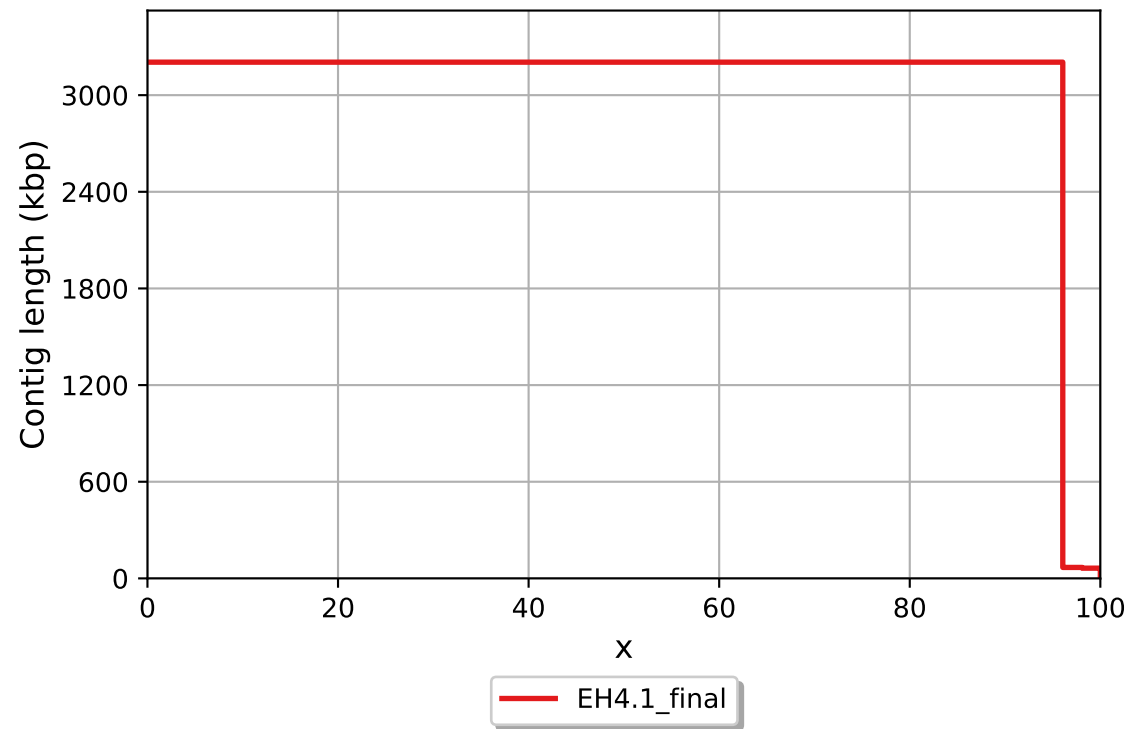

Supplement: Supplementary file 1 [file microorganisms-14-00921-s001.zip › Supplementary File S1/QUAST/EH4.1/basic_stats/Nx_plot.pdf]
