# Supplementary material for: Candidate Genomic Features Associated with Persistence in Enterococcus spp
Source: Microorganisms. 2026 Apr 19;14(4):921. doi: 10.3390/microorganisms14040921 (PMC13119490; doi:10.3390/microorganisms14040921)
Supplement: Supplementary file 1 [file microorganisms-14-00921-s001.zip › Supplementary File S1/QUAST/EH4.1/contigs_reports/misassemblies_frcurve_plot.pdf]

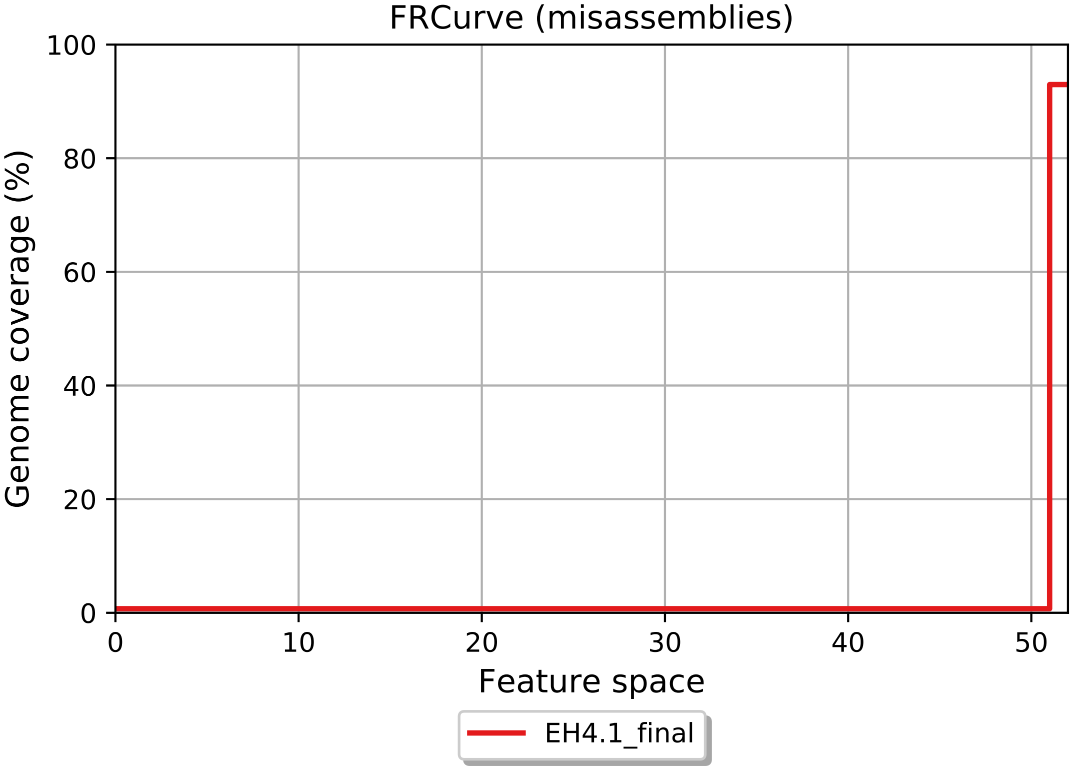
FRCurve (misassemblies)
Genome coverage (%)
100
80
60
40
20
0
0
10
20
30
40
50
Feature space
EH4.1_final
