# Supplementary material for: Candidate Genomic Features Associated with Persistence in Enterococcus spp
Source: Microorganisms. 2026 Apr 19;14(4):921. doi: 10.3390/microorganisms14040921 (PMC13119490; doi:10.3390/microorganisms14040921)
Supplement: Supplementary file 1 [file microorganisms-14-00921-s001.zip › Supplementary File S1/QUAST/EH4.2/basic_stats/Nx_plot.pdf]

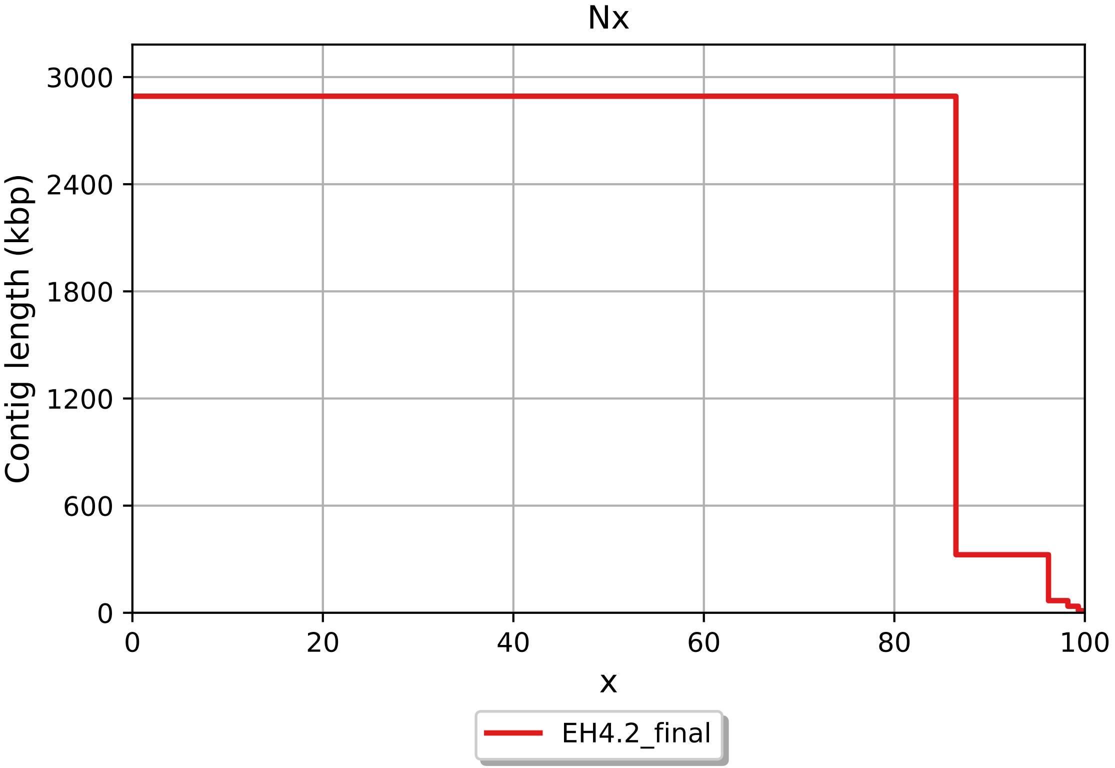

Nx
3000
2400
1800
1200
600
0
Contig length (kbp)
0
20
40
60
80
100
x
EH4.2_final
