# Supplementary material for: Candidate Genomic Features Associated with Persistence in Enterococcus spp
Source: Microorganisms. 2026 Apr 19;14(4):921. doi: 10.3390/microorganisms14040921 (PMC13119490; doi:10.3390/microorganisms14040921)
Supplement: Supplementary file 1 [file microorganisms-14-00921-s001.zip › Supplementary File S1/QUAST/EH4.2/report.pdf]

Report

	EH4.2_final
# contigs (>= 0 bp)	7
# contigs (>= 1000 bp)	6
# contigs (>= 5000 bp)	6
# contigs (>= 10000 bp)	6
# contigs (>= 25000 bp)	4
# contigs (>= 50000 bp)	3
Total length (>= 0 bp)	3345856
Total length (>= 1000 bp)	3345525
Total length (>= 5000 bp)	3345525
Total length (>= 10000 bp)	3345525
Total length (>= 25000 bp)	3322601
Total length (>= 50000 bp)	3285849
# contigs	6
Largest contig	2892684
Total length	3345525
Reference length	2870381
GC (%)	37.20
Reference GC (%)	37.47
N50	2892684
NG50	2892684
N90	325154
NG90	2892684
auN	2534605.3
auNG	2954167.2
L50	1
LG50	1
L90	2
LG90	1
# misassemblies	51
# misassembled contigs	3
Misassembled contigs length	3229799
# local misassemblies	49
# scaffold gap ext. mis.	0
# scaffold gap loc. mis.	0
# unaligned mis. contigs	1
# unaligned contigs	0 + 6 part
Unaligned length	680893
Genome fraction (%)	92.073
Duplication ratio	1.011
# N's per 100 kbp	0.00
# mismatches per 100 kbp	822.02
# indels per 100 kbp	25.03
Largest alignment	383955
Total aligned length	2664530
NA50	88301
NGA50	103963
NA90	-
NGA90	11803
auNA	133192.4
auNGA	155240.3
LA50	9
LGA50	7
LA90	-
LGA90	35

All statistics are based on contigs of size >= 500 bp, unless otherwise noted (e.g., "# contigs (>= 0 bp)" and "Total length (>= 0 bp)" include all contigs).

Misassemblies report

	EH4.2_final
# misassemblies	51
# contig misassemblies	51
# c. relocations	47
# c. translocations	4
# c. inversions	0
# scaffold misassemblies	0
# s. relocations	0
# s. translocations	0
# s. inversions	0
# misassembled contigs	3
Misassembled contigs length	3229799
# local misassemblies	49
# scaffold gap ext. mis.	0
# scaffold gap loc. mis.	0
# unaligned mis. contigs	1
# mismatches	21903
# indels	667
# indels (<= 5 bp)	571
# indels (> 5 bp)	96
Indels length	6440

All statistics are based on contigs of size >= 500 bp, unless otherwise noted (e.g., "# contigs (>= 0 bp)" and "Total length (>= 0 bp)" include all contigs).

Unaligned report

	EH4.2_final
# fully unaligned contigs	0
Fully unaligned length	0
# partially unaligned contigs	6
Partially unaligned length	680893
# N's	0

All statistics are based on contigs of size >= 500 bp, unless otherwise noted (e.g., "# contigs (>= 0 bp)" and "Total length (>= 0 bp)" include all contigs).

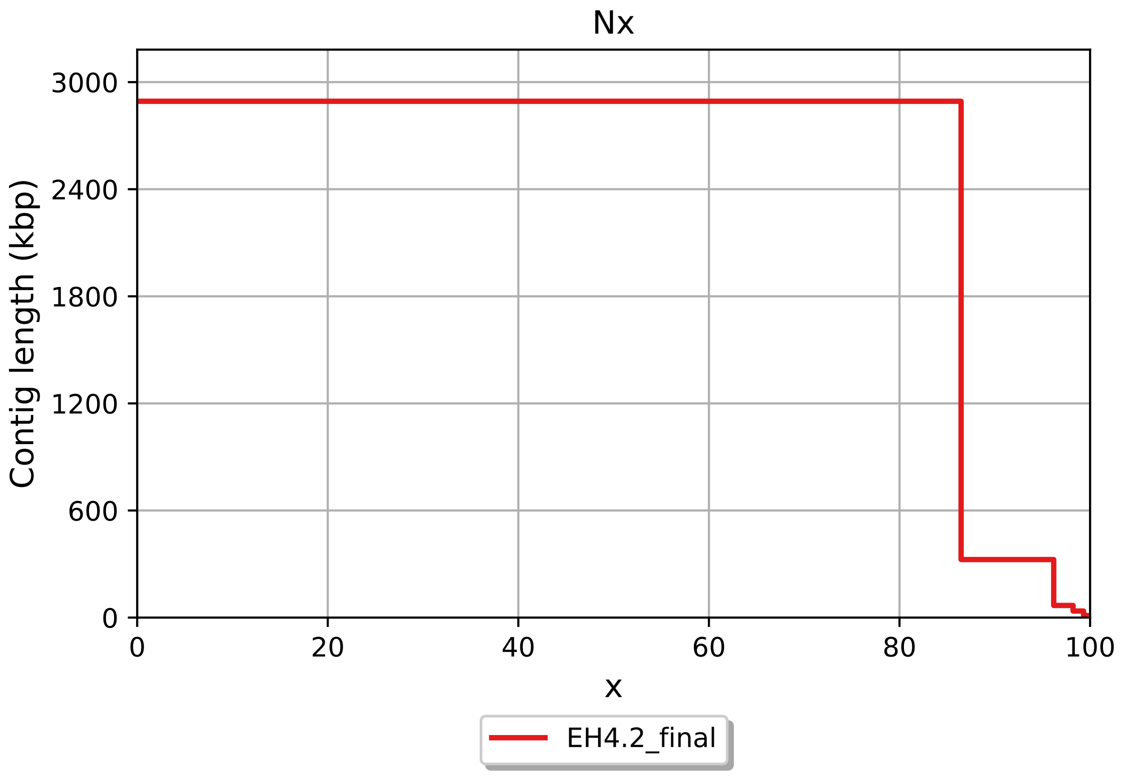
Nx
Contig length (kbp)
3000
2400
1800
1200
600
0
0
20
40
60
80
100
x
EH4.2_final

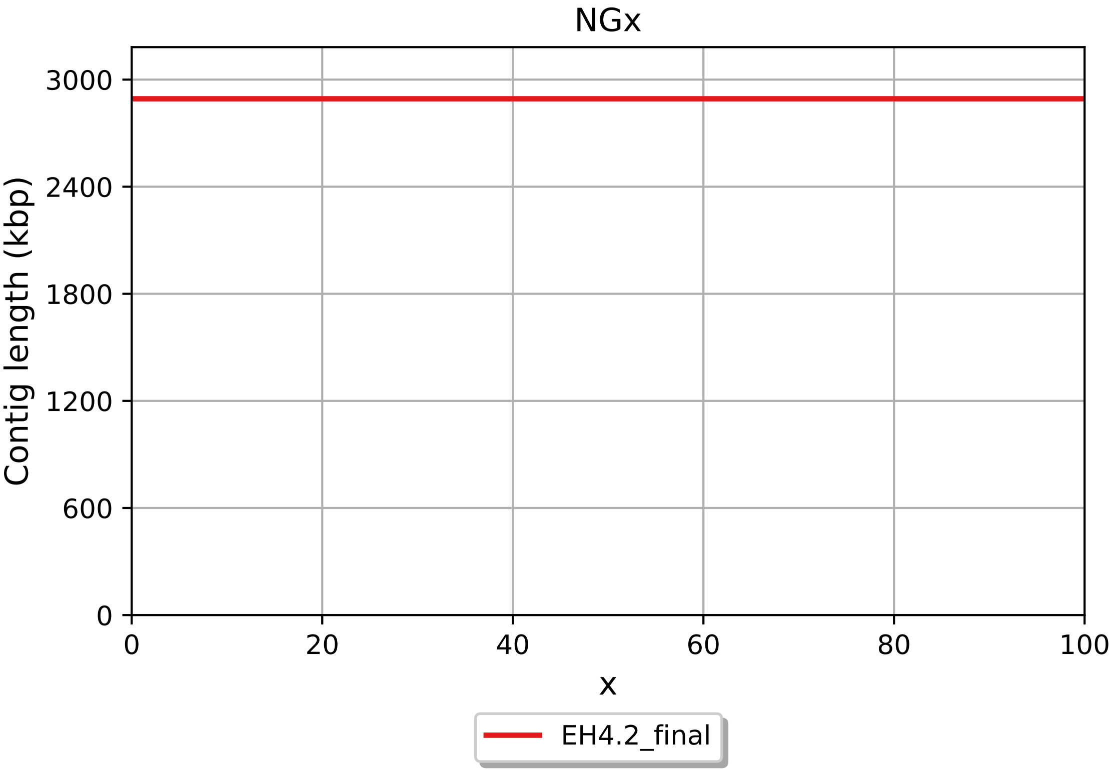

NGx
3000
2400
1800
1200
600
0
Contig length (kbp)
0
20
40
60
80
100
x
EH4.2_final

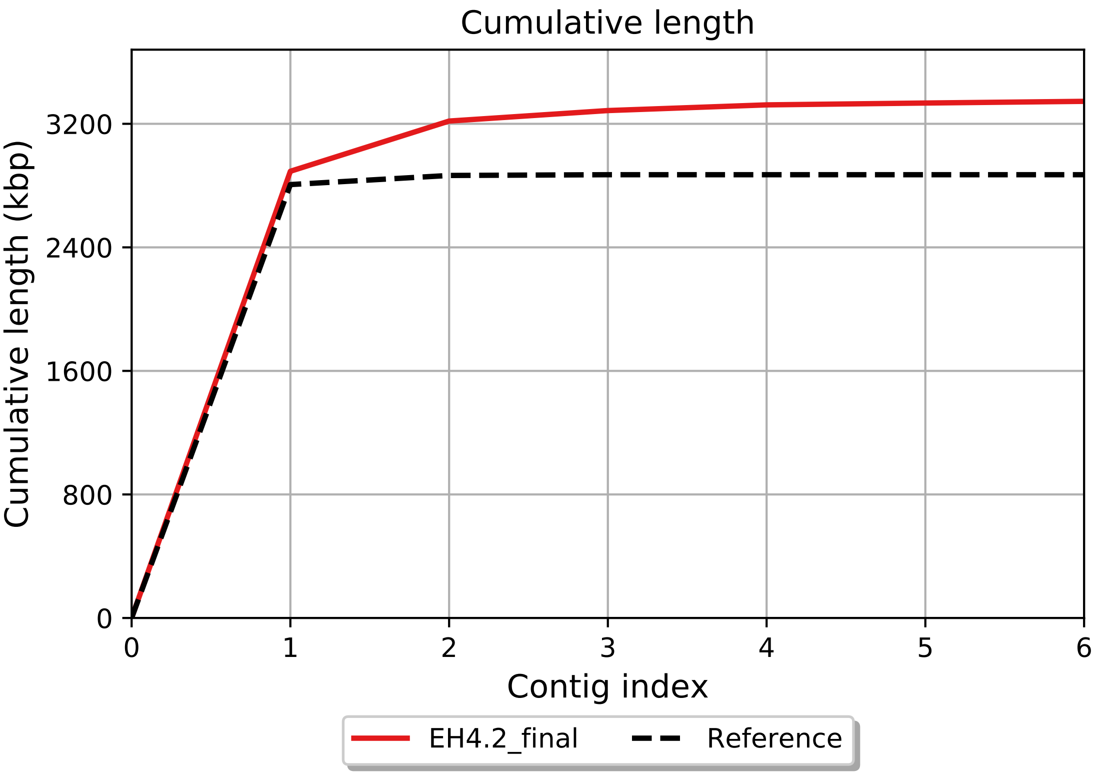

Cumulative length
Cumulative length (kbp)
0
800
1600
2400
3200
0
1
2
3
4
5
6
Contig index
EH4.2_final
Reference

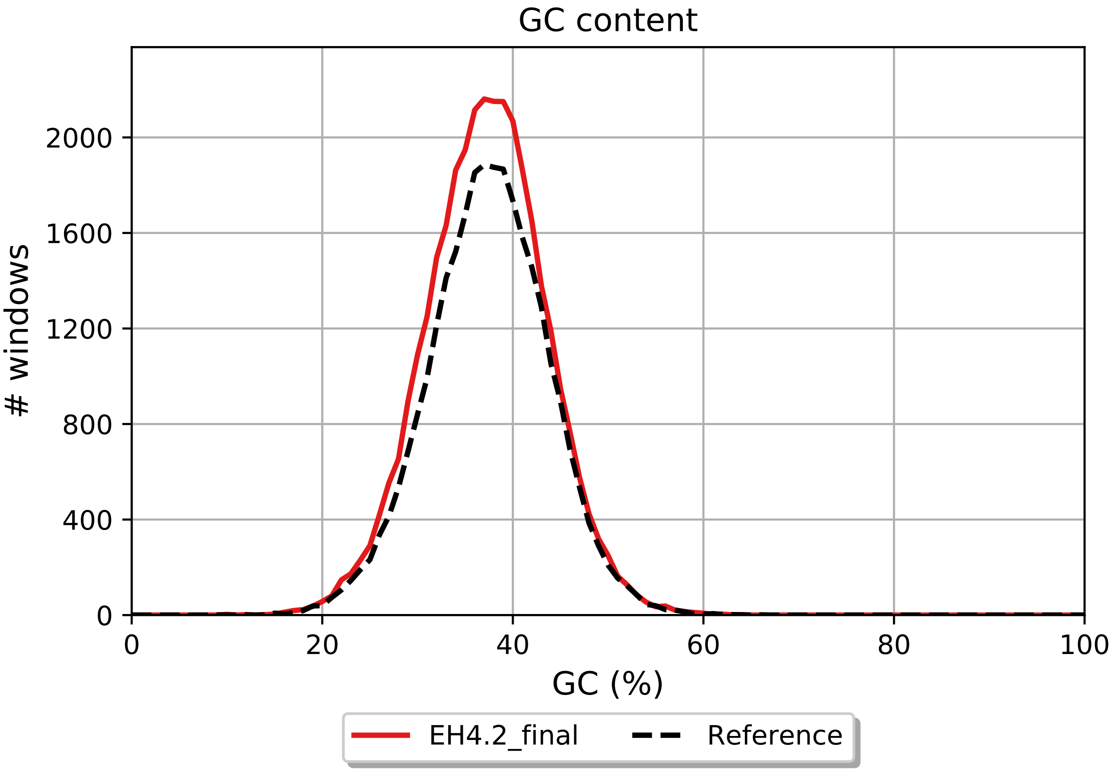
GC content
windows
0
400
800
1200
1600
2000
0
20
40
60
80
100
GC (%)
EH4.2_final
Reference

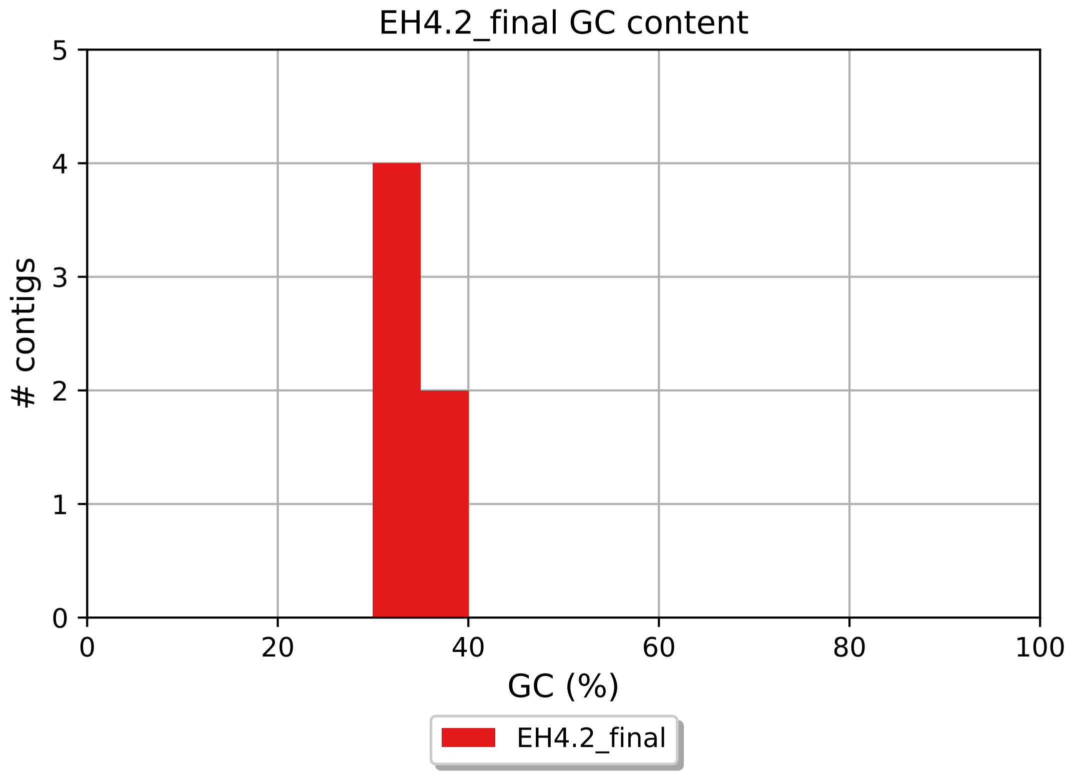

EH4.2_final GC content
5
4
3
2
1
0
contigs
0
20
40
60
80
100
GC (%)
EH4.2_final

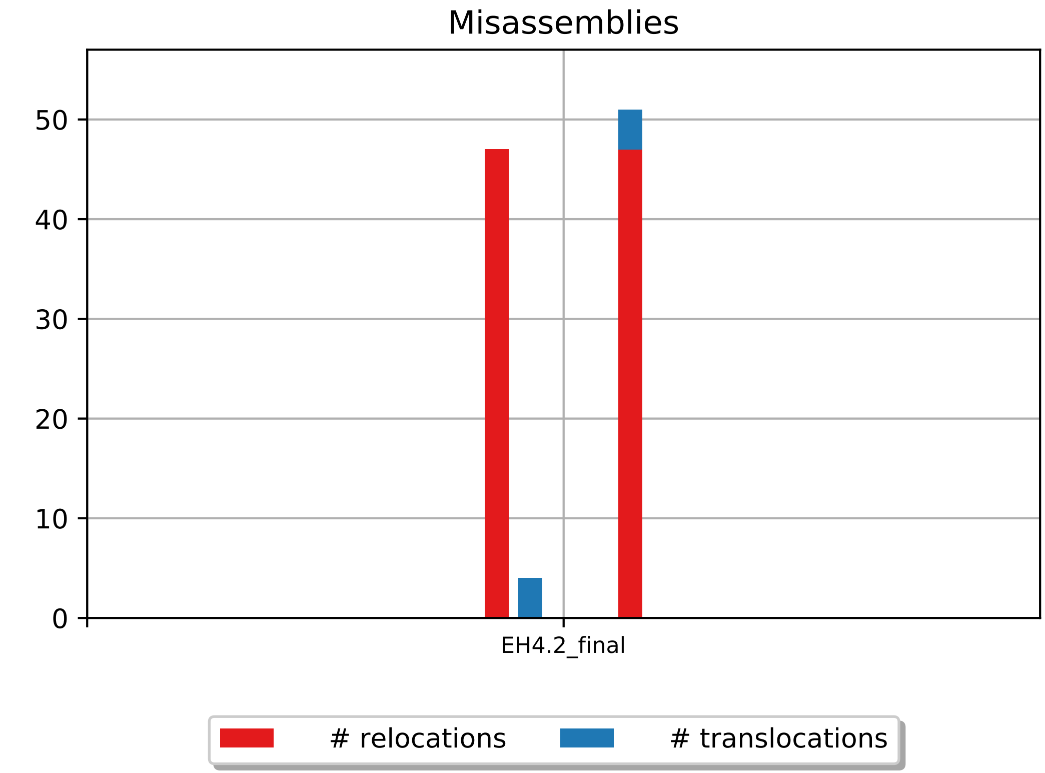
Misassemblies
50
40
30
20
10
0
EH4.2_final
relocations
translocations

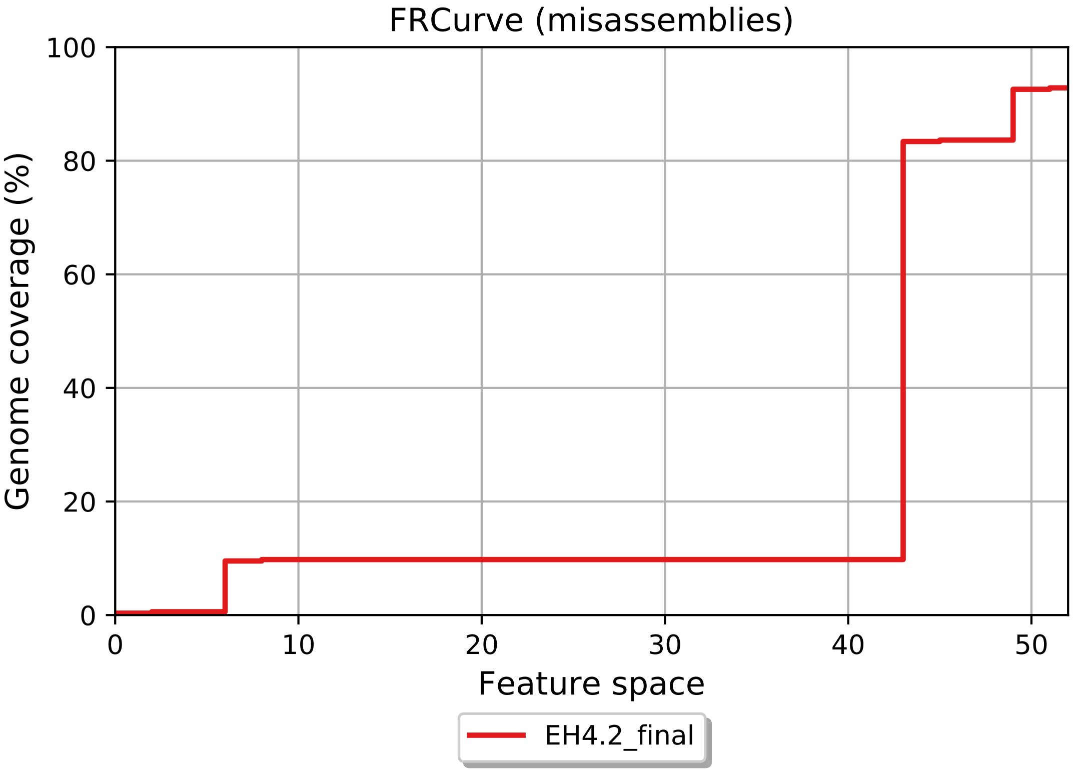

FRCurve (misassemblies)
Genome coverage (%)
0
20
40
60
80
100
0
10
20
30
40
50
Feature space
EH4.2_final

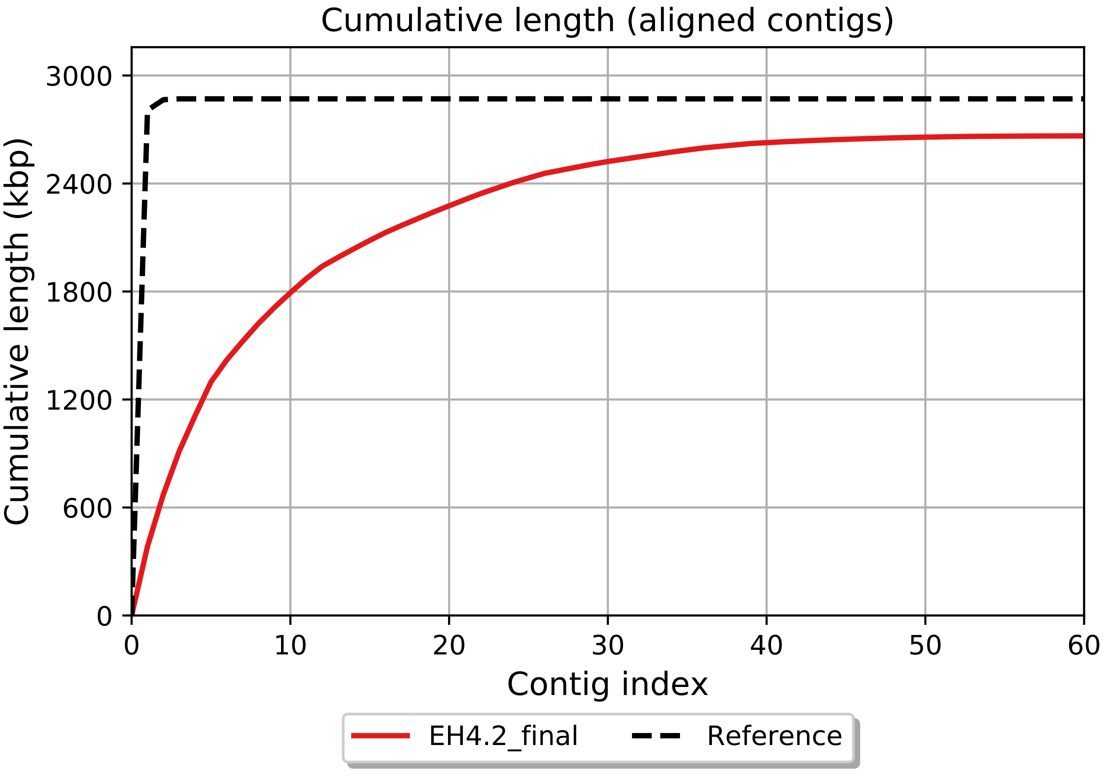

Cumulative length (aligned contigs)
Cumulative length (kbp)
0
600
1200
1800
2400
3000
0
10
20
30
40
50
60
Contig index
EH4.2_final
Reference

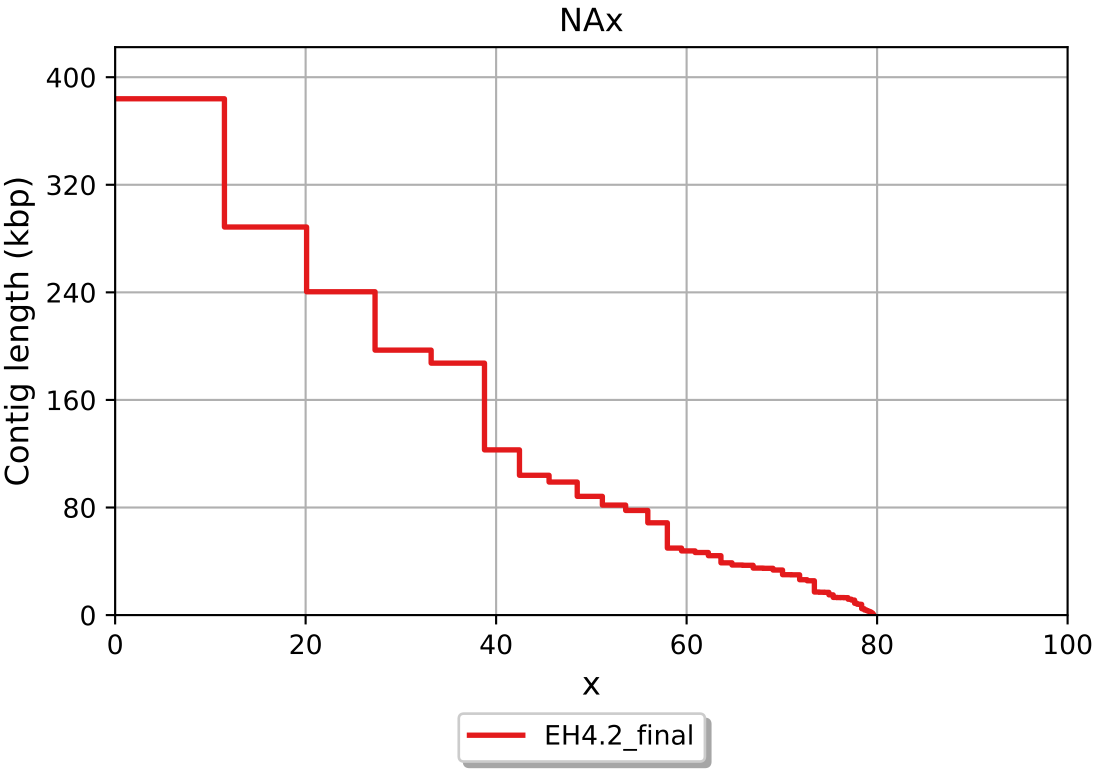

NAx
Contig length (kbp)
400
320
240
160
80
0
0
20
40
60
80
100
x
EH4.2_final

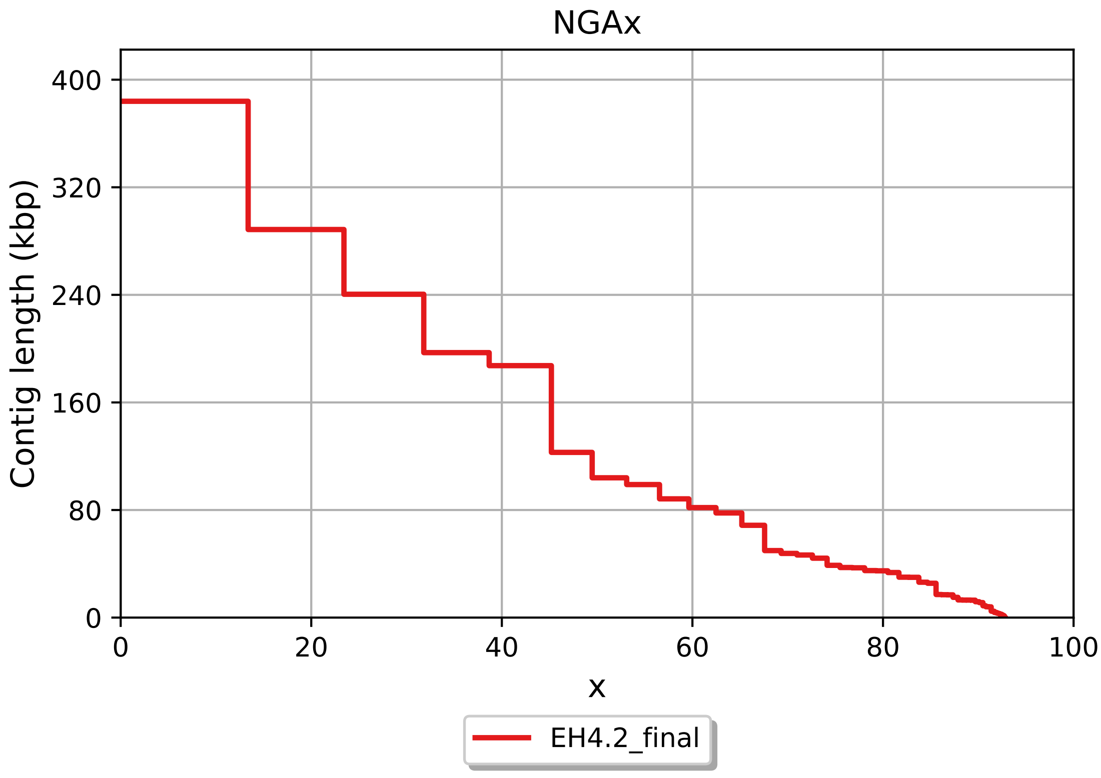

NGAx
400
320
240
160
80
0
Contig length (kbp)
0
20
40
60
80
100
x
EH4.2_final
