# Supplementary material for: Candidate Genomic Features Associated with Persistence in Enterococcus spp
Source: Microorganisms. 2026 Apr 19;14(4):921. doi: 10.3390/microorganisms14040921 (PMC13119490; doi:10.3390/microorganisms14040921)
Supplement: Supplementary file 1 [file microorganisms-14-00921-s001.zip › Supplementary File S1/QUAST/EH4.3/aligned_stats/NGAx_plot.pdf]

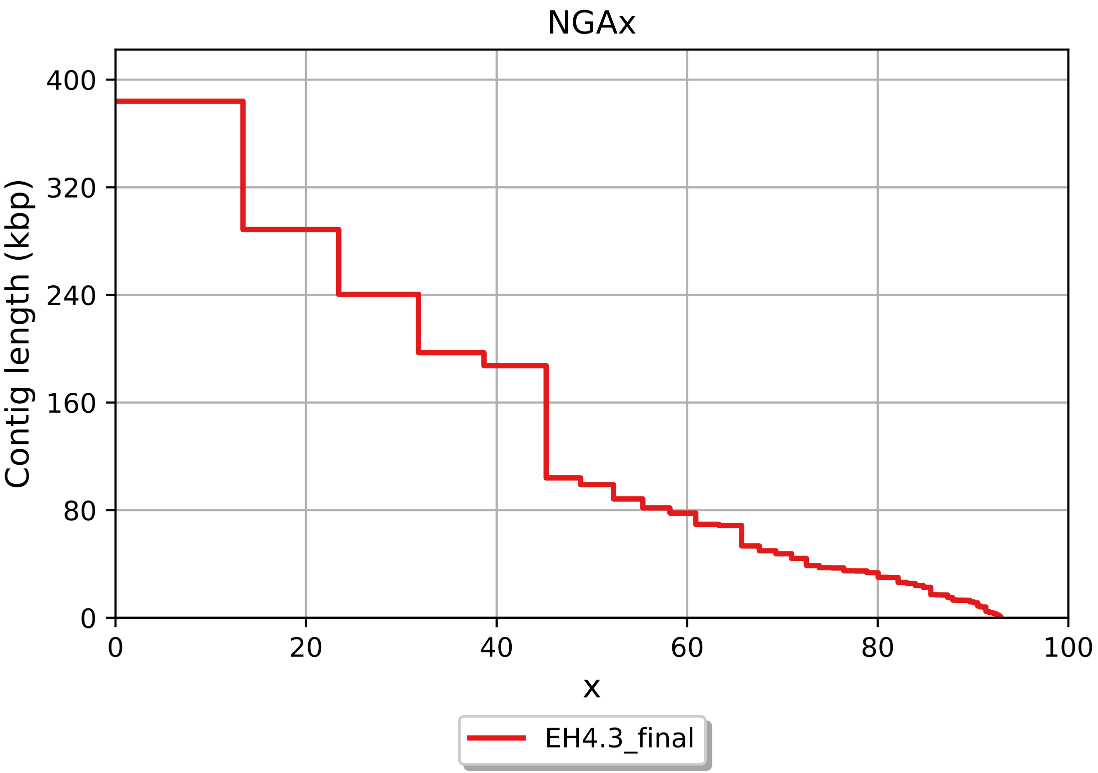
NGAx
Contig length (kbp)
400
320
240
160
80
0
0
20
40
60
80
100
x
EH4.3_final
