# Supplementary material for: Candidate Genomic Features Associated with Persistence in Enterococcus spp
Source: Microorganisms. 2026 Apr 19;14(4):921. doi: 10.3390/microorganisms14040921 (PMC13119490; doi:10.3390/microorganisms14040921)
Supplement: Supplementary file 1 [file microorganisms-14-00921-s001.zip › Supplementary File S1/QUAST/EH4.3/basic_stats/cumulative_plot.pdf]

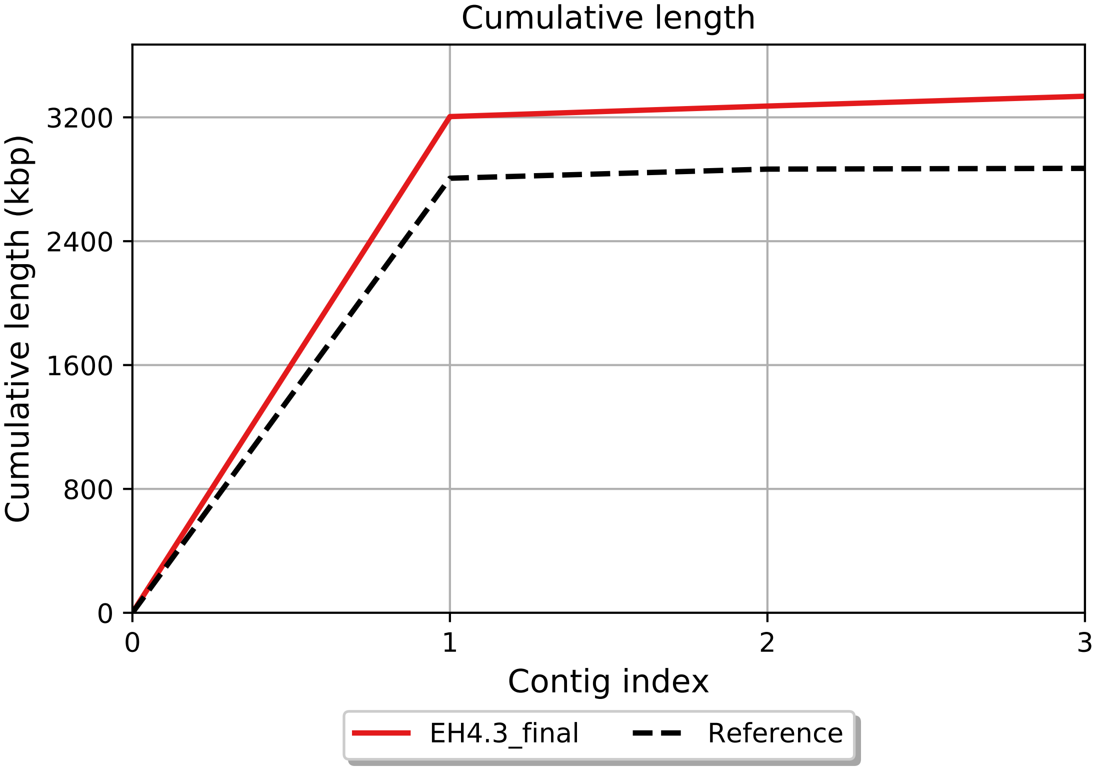

Cumulative length
Cumulative length (kbp)
3200
2400
1600
800
0
0
1
2
3
Contig index
EH4.3_final
Reference
