# Supplementary material for: Candidate Genomic Features Associated with Persistence in Enterococcus spp
Source: Microorganisms. 2026 Apr 19;14(4):921. doi: 10.3390/microorganisms14040921 (PMC13119490; doi:10.3390/microorganisms14040921)
Supplement: Supplementary file 1 [file microorganisms-14-00921-s001.zip › Supplementary File S1/QUAST/EH4.3/icarus_viewers/alignment_viewer.html]

|  |  |  |
| --- | --- | --- |
| Main menu  Icarus  **QUAST Contig Browser** | Move << < > >> zoom +5x +2x –2x –5x \| start end Show misassemblies: relocations (50) translocations (2) inversions (0) local (48) | Search contig or gene: |

**Contig alignment viewer**. Contigs aligned to genomic

Hide
Show annotation
Hide
Show read coverage and GC distribution
Show physical
Show physical
Hide GC %
Hide GC %

+
-
Reset

+
-
Reset
