# Supplementary material for: Candidate Genomic Features Associated with Persistence in Enterococcus spp
Source: Microorganisms. 2026 Apr 19;14(4):921. doi: 10.3390/microorganisms14040921 (PMC13119490; doi:10.3390/microorganisms14040921)
Supplement: Supplementary file 1 [file microorganisms-14-00921-s001.zip › Supplementary File S1/QUAST/EH4.3/report.pdf]

Report

	EH4.3_final
# contigs (>= 0 bp)	3
# contigs (>= 1000 bp)	3
# contigs (>= 5000 bp)	3
# contigs (>= 10000 bp)	3
# contigs (>= 25000 bp)	3
# contigs (>= 50000 bp)	3
Total length (>= 0 bp)	3336250
Total length (>= 1000 bp)	3336250
Total length (>= 5000 bp)	3336250
Total length (>= 10000 bp)	3336250
Total length (>= 25000 bp)	3336250
Total length (>= 50000 bp)	3336250
# contigs	3
Largest contig	3204789
Total length	3336250
Reference length	2870381
GC (%)	37.20
Reference GC (%)	37.47
N50	3204789
NG50	3204789
N90	3204789
NG90	3204789
auN	3081101.2
auNG	3581170.6
L50	1
LG50	1
L90	1
LG90	1
# misassemblies	52
# misassembled contigs	1
Misassembled contigs length	3204789
# local misassemblies	48
# scaffold gap ext. mis.	0
# scaffold gap loc. mis.	0
# unaligned mis. contigs	2
# unaligned contigs	0 + 3 part
Unaligned length	667799
Genome fraction (%)	92.076
Duplication ratio	1.012
# N's per 100 kbp	0.00
# mismatches per 100 kbp	824.58
# indels per 100 kbp	25.30
Largest alignment	383965
Total aligned length	2667662
NA50	81677
NGA50	98931
NA90	-
NGA90	11803
auNA	131019.4
auNGA	152284.1
LA50	9
LGA50	7
LA90	-
LGA90	37

All statistics are based on contigs of size >= 500 bp, unless otherwise noted (e.g., "# contigs (>= 0 bp)" and "Total length (>= 0 bp)" include all contigs).

Misassemblies report

	EH4.3_final
# misassemblies	52
# contig misassemblies	52
# c. relocations	50
# c. translocations	2
# c. inversions	0
# scaffold misassemblies	0
# s. relocations	0
# s. translocations	0
# s. inversions	0
# misassembled contigs	1
Misassembled contigs length	3204789
# local misassemblies	48
# scaffold gap ext. mis.	0
# scaffold gap loc. mis.	0
# unaligned mis. contigs	2
# mismatches	21997
# indels	675
# indels (<= 5 bp)	579
# indels (> 5 bp)	96
Indels length	6448

All statistics are based on contigs of size >= 500 bp, unless otherwise noted (e.g., "# contigs (>= 0 bp)" and "Total length (>= 0 bp)" include all contigs).

Unaligned report

	EH4.3_final
# fully unaligned contigs	0
Fully unaligned length	0
# partially unaligned contigs	3
Partially unaligned length	667799
# N's	0

All statistics are based on contigs of size >= 500 bp, unless otherwise noted (e.g., "# contigs (>= 0 bp)" and "Total length (>= 0 bp)" include all contigs).

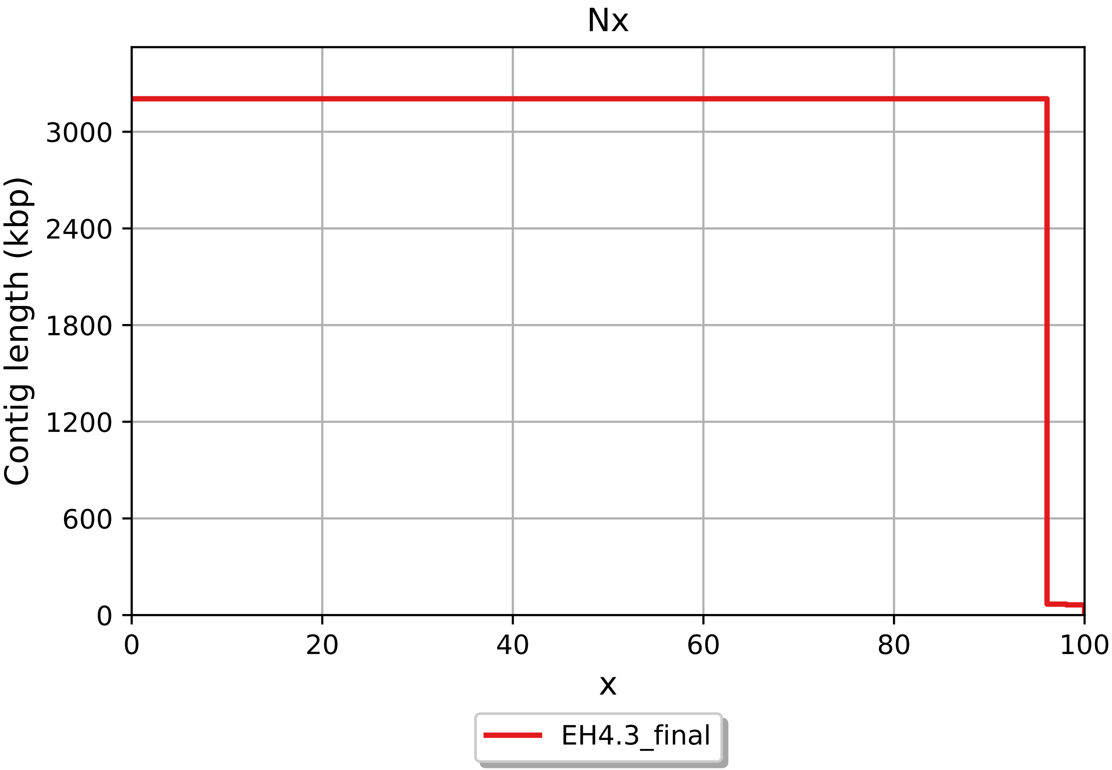
Nx
Contig length (kbp)
3000
2400
1800
1200
600
0
0
20
40
60
80
100
x
EH4.3_final

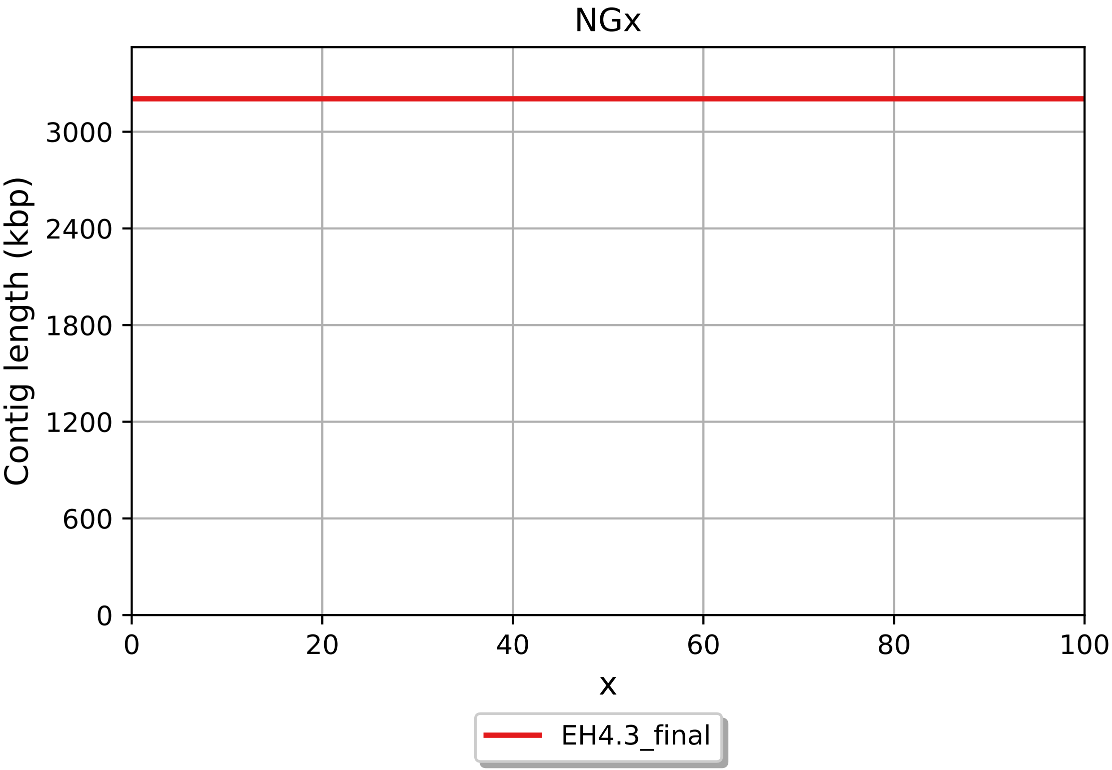
NGx
Contig length (kbp)
3000
2400
1800
1200
600
0
0
20
40
60
80
100
x
EH4.3_final

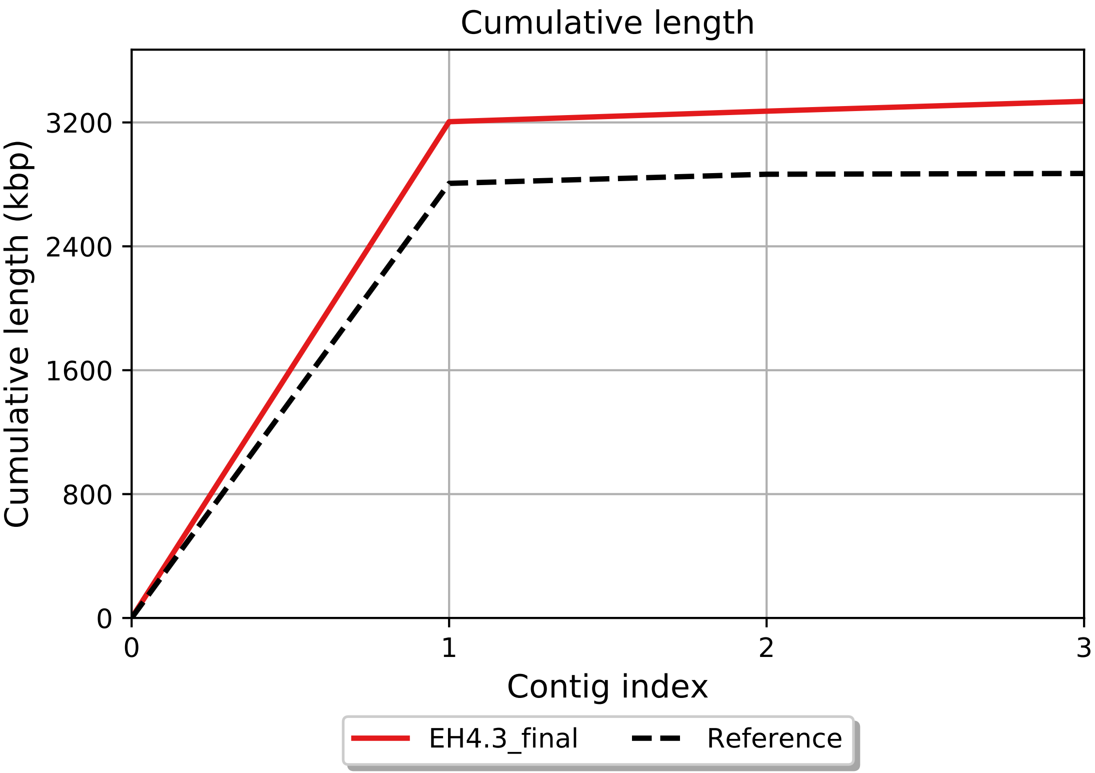
Cumulative length
Cumulative length (kbp)
3200
2400
1600
800
0
0
1
2
3
Contig index
EH4.3_final
Reference

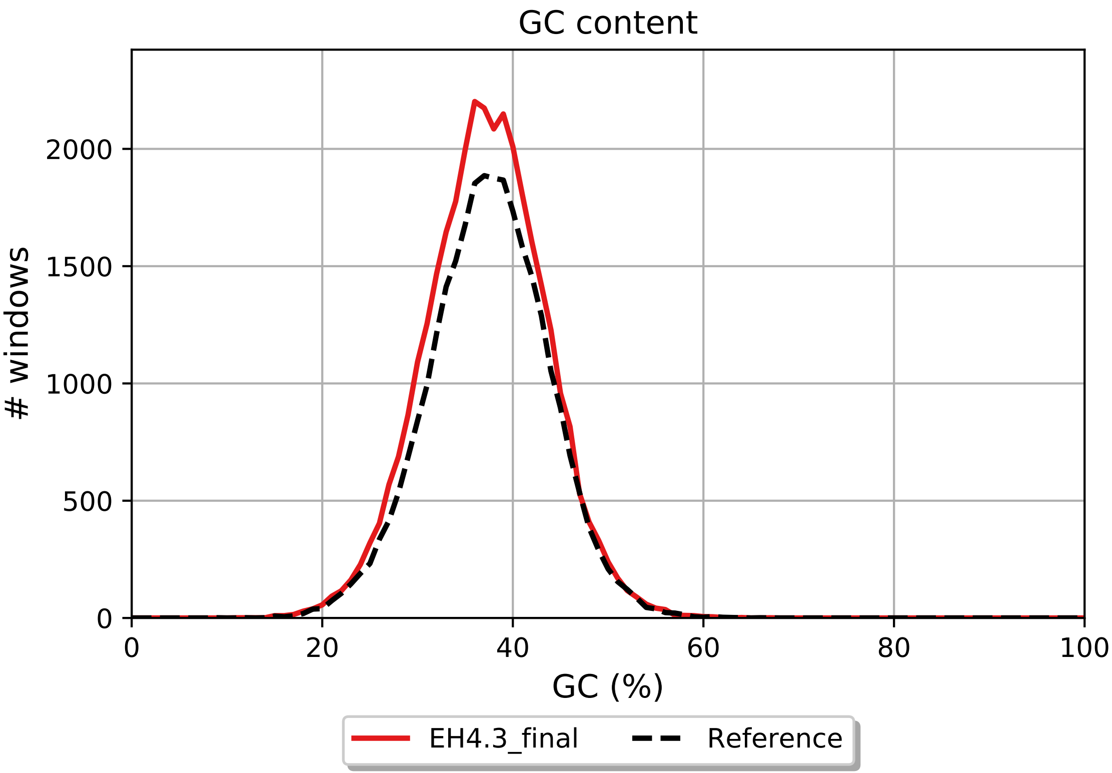
GC content
windows
0
500
1000
1500
2000
0
20
40
60
80
100
GC (%)
EH4.3_final
Reference

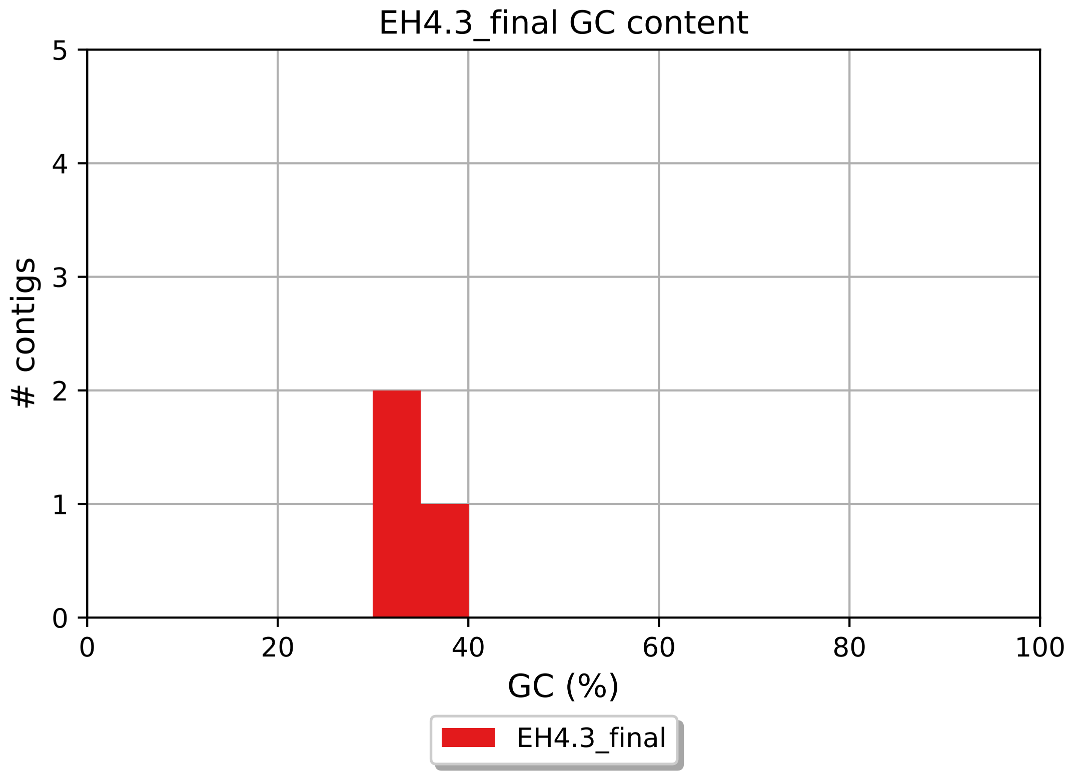
EH4.3_final GC content
contigs
GC (%)
0
1
2
3
4
5
0
20
40
60
80
100
EH4.3_final

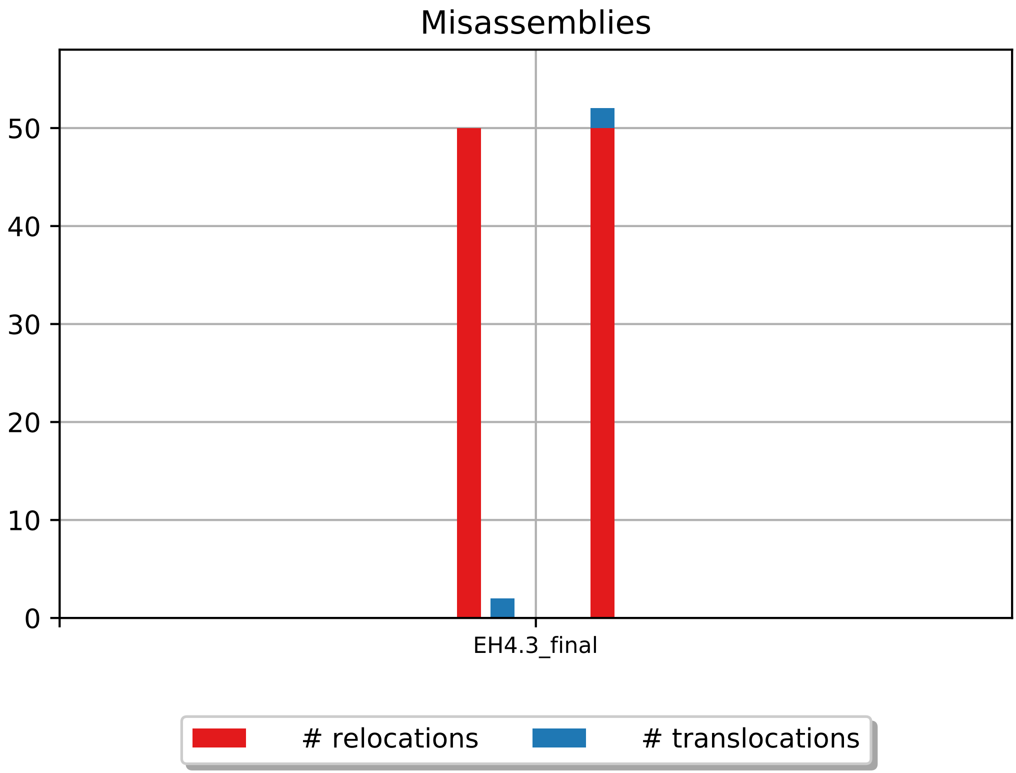

Misassemblies
50
40
30
20
10
0
EH4.3_final
relocations
translocations

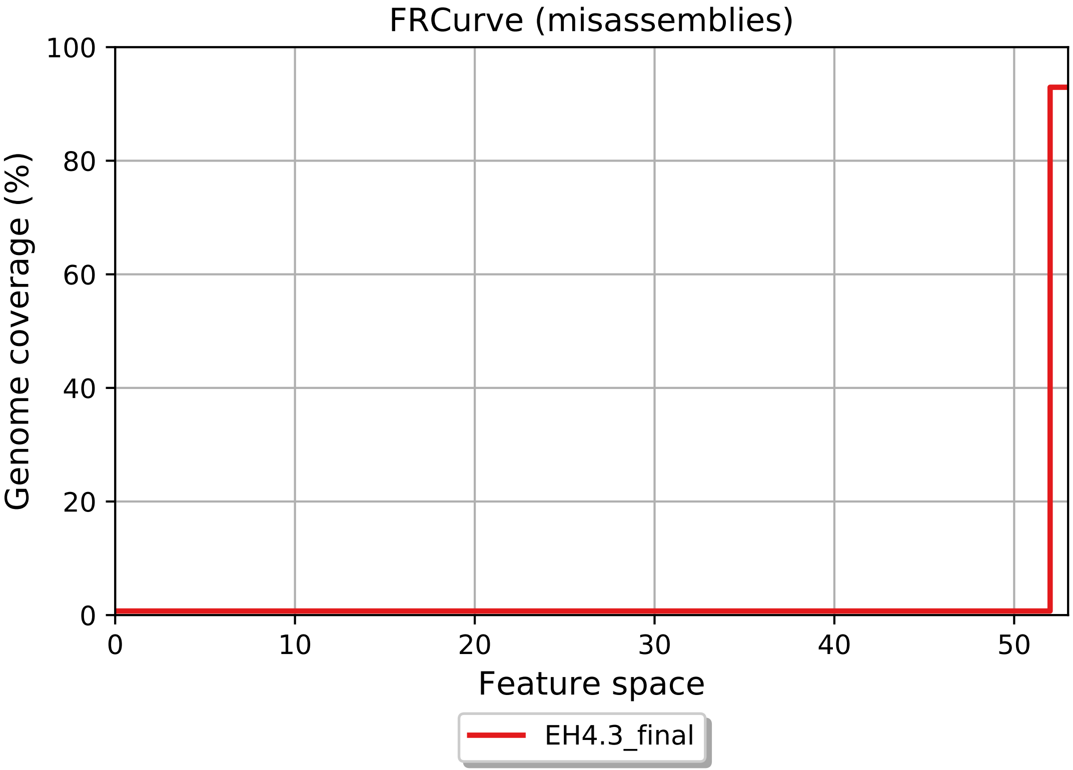
FRCurve (misassemblies)
Genome coverage (%)
100
80
60
40
20
0
0
10
20
30
40
50
Feature space
EH4.3_final

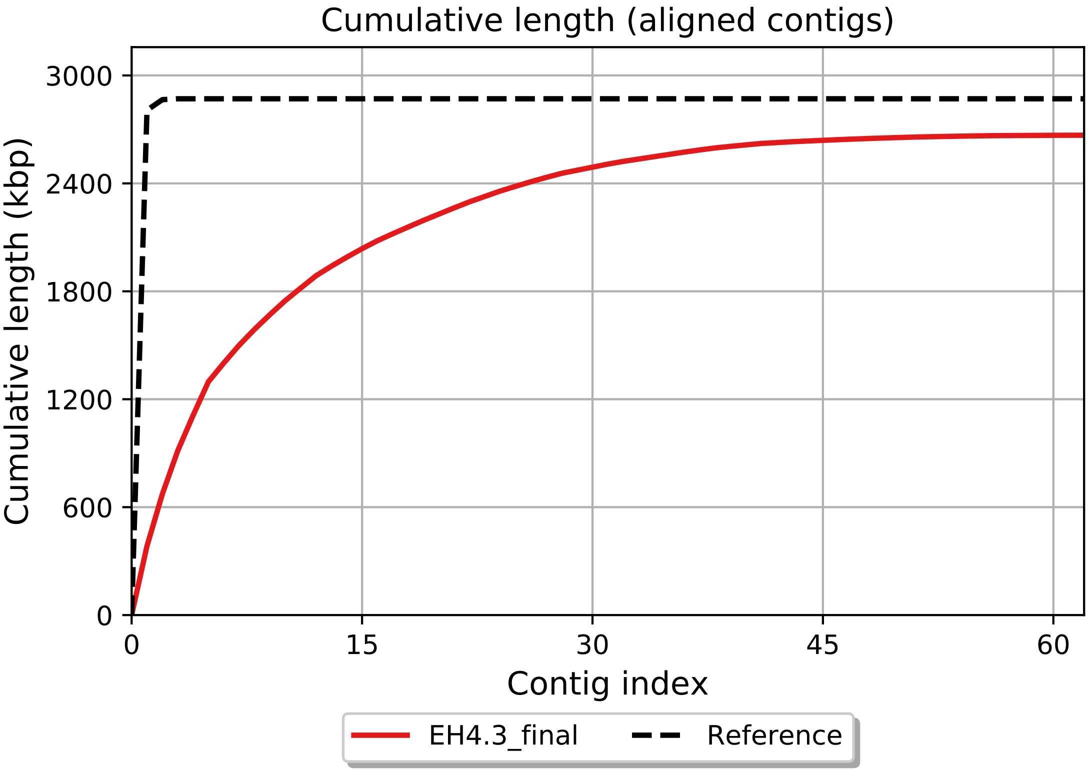
Cumulative length (aligned contigs)
Cumulative length (kbp)
3000
2400
1800
1200
600
0
0
15
30
45
60
Contig index
EH4.3_final
Reference

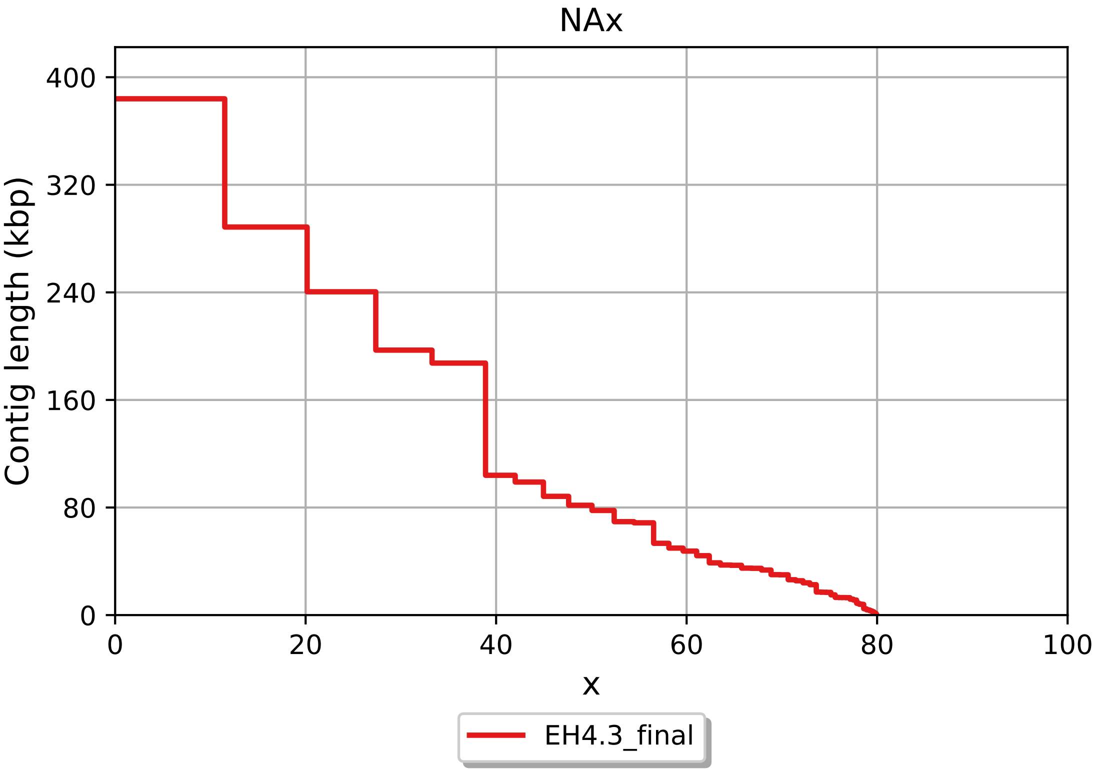
NAx
Contig length (kbp)
400
320
240
160
80
0
0
20
40
60
80
100
x
EH4.3_final

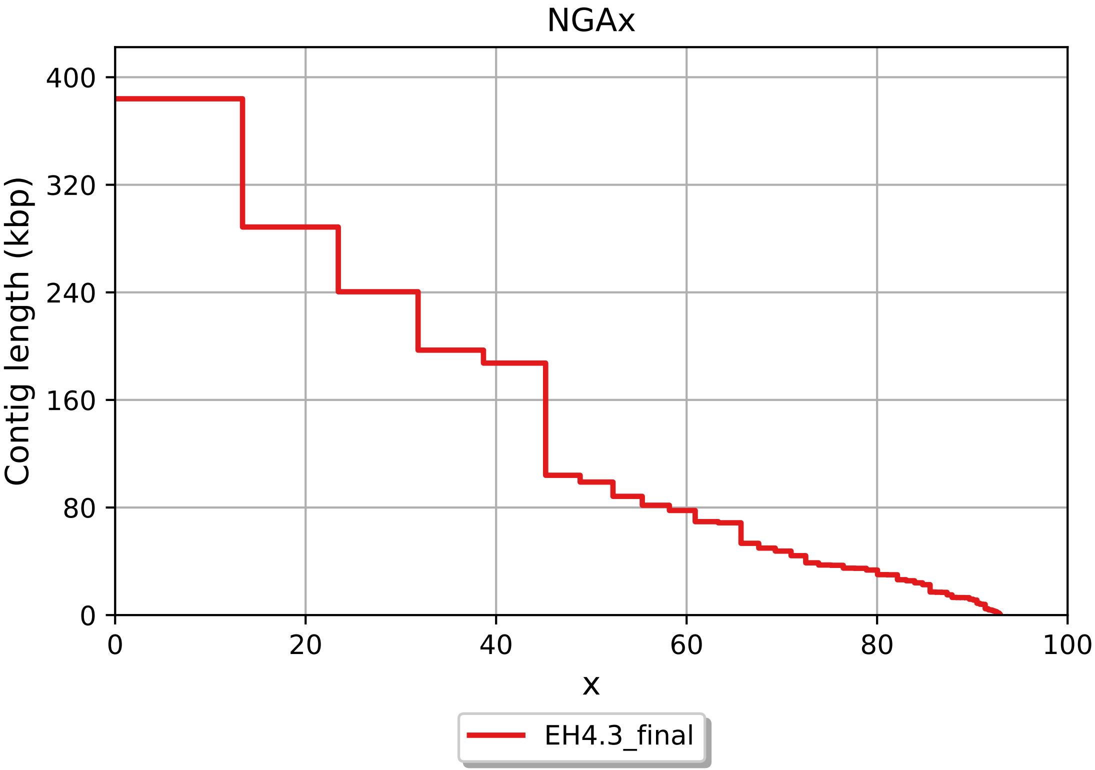
NGAx
400
320
240
160
80
0
0
20
40
60
80
100
x
Contig length (kbp)
EH4.3_final
