# Supplementary material for: Candidate Genomic Features Associated with Persistence in Enterococcus spp
Source: Microorganisms. 2026 Apr 19;14(4):921. doi: 10.3390/microorganisms14040921 (PMC13119490; doi:10.3390/microorganisms14040921)
Supplement: Supplementary file 1 [file microorganisms-14-00921-s001.zip › Supplementary File S1/QUAST/EH4_negative/basic_stats/EH4_negative_final_GC_content_plot.pdf]

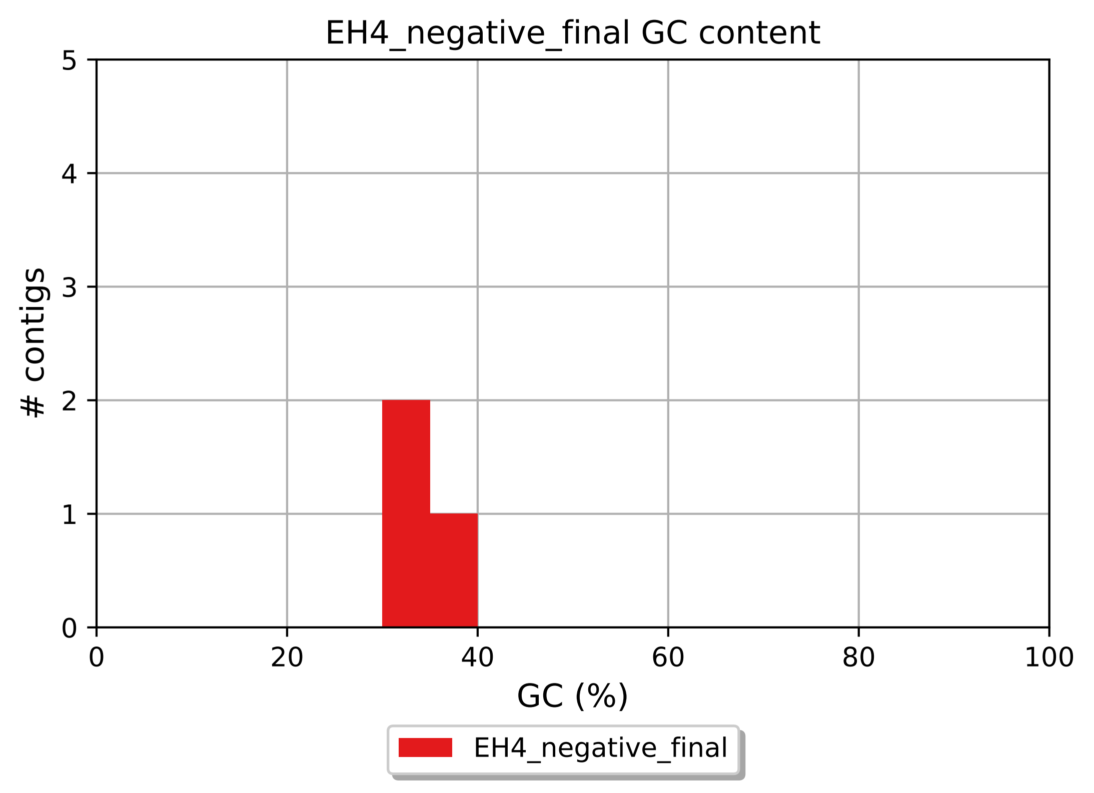

EH4_negative_final GC content
contigs
0
1
2
3
4
5
0
20
40
60
80
100
GC (%)
EH4_negative_final
