# Supplementary material for: Candidate Genomic Features Associated with Persistence in Enterococcus spp
Source: Microorganisms. 2026 Apr 19;14(4):921. doi: 10.3390/microorganisms14040921 (PMC13119490; doi:10.3390/microorganisms14040921)
Supplement: Supplementary file 1 [file microorganisms-14-00921-s001.zip › Supplementary File S1/QUAST/EH4_negative/icarus.html]

# Icarus

**QUAST Contig Browser** by CAB

**Assemblies:** EH4\_negative\_final
| Contig size viewer |
| QUAST report |

  

Contig alignment viewer

Aligned to sequences from genomic.fna

Fragments: 3, length: 2 870 381 bp, mean genome fraction: 91.828%,
misassembled blocks: 112
